# Combining geometric constraint and redox non-innocence within an ambiphilic PBiP pincer ligand†

**DOI:** 10.1039/d4sc00197d

**Published:** 2024-03-18

**Authors:** Peter Coburger, Ana Guilherme Buzanich, Franziska Emmerling, Josh Abbenseth

**Affiliations:** a Department of Inorganic Chemistry, Technische Universität München Lichtenbergstr. 4 85747 Garching Germany; b Department of Materials Chemistry, Federal Institute for Materials Research and Testing Richard-Willstätter-Str. 11 12489 Berlin Germany; c Institut für Chemie, Humboldt-Universität zu Berlin Brook-Taylor-Str. 2 12489 Berlin Germany josh.abbenseth@hu-berlin.de

## Abstract

The synthesis of the first pincer ligand featuring a strictly T-shaped group 15 element and its coordination behaviour towards transition metals is described. The platform is itself derived from a trianionic redox non-innocent NNN scaffold. In addition to providing a rigid coordination environment to constrain a Bi centre in a T-shaped geometry to manipulate its frontier molecular orbital constitution, the NNN chelate displays highly covalent bonding towards the geometrically constrained Bi centre. The formation of intriguing ambiphilic Bi–M bonding interactions is demonstrated upon formation of a pincer complex as well as a multimetallic cluster. All compounds are comprehensively characterised by spectroscopic methods including X-ray Absorption Near Edge Structure (XANES) spectroscopy and complemented by DFT calculations.

## Abstract 2

The first pincer ligand featuring a strictly T-shaped pnictogen donor moiety was synthesised. The PBiP ligand's redox activity facilitates unprecedented ambiphilic bonding of the Bi centre with transition metals through the Bi(6p) orbital.

## Graphical abstract

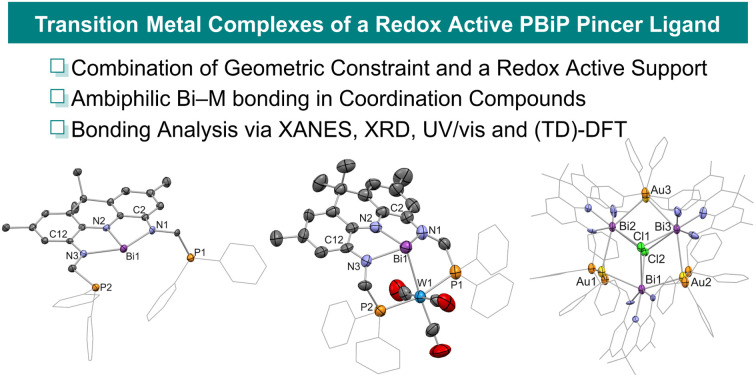

## Introduction

**Fig. 1:**
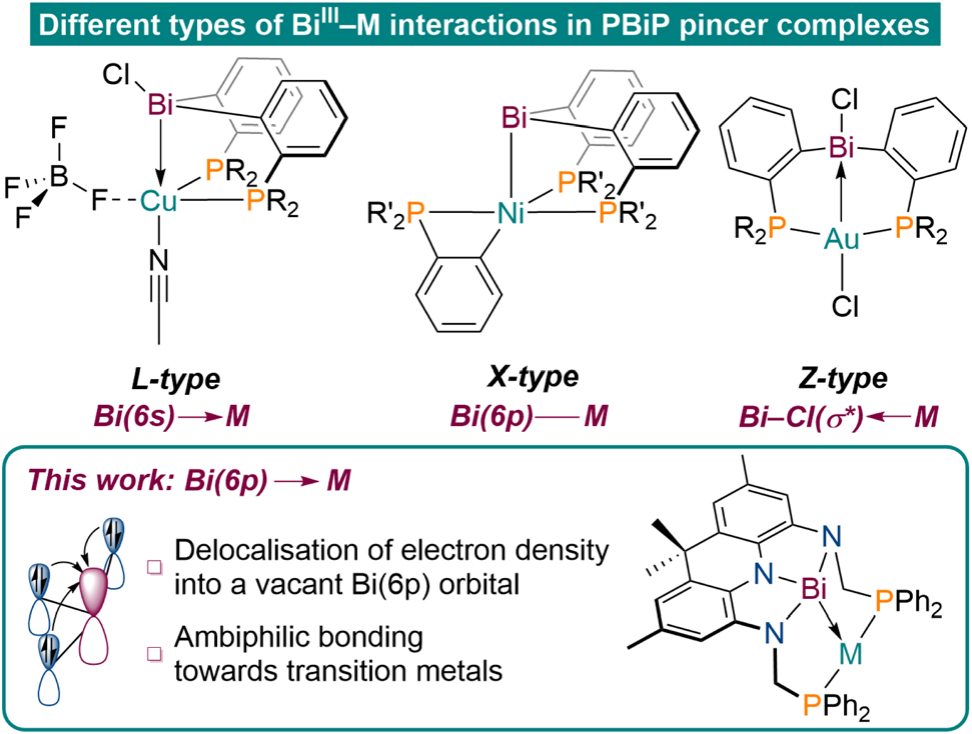
Representative examples of established bonding modes of Bi(iii)R_3_ derived transition metal pincer complexes (top, R = Ph, R′ = ^*i*^Pr);^7–9,12^ our results on realizing ambiphilic Bi(6p)–M bonding (bottom).

Tridentate pincer ligands have emerged as highly versatile tools to tune the reactivity of transition metal centres in the context of small molecule activation reactions and catalysis.^1–5^ While central donor functionalities based on 2nd and 3rd row elements are well investigated and utilised in various applications, the chemistry of ligand systems involving heavier elements is comparably underdeveloped. In particular, studies on pincer ligands featuring elements of the 6th period are scarce and their chemistry remains largely unexplored.^6–13^ In this context, Bi stands out as a particularly fascinating element because of its high natural abundance, low toxicity, multiple accessible oxidation states, and metallic character. These attributes have been effectively leveraged in catalytic applications in recent years.^14–20^ PBiP pincer ligands featuring a neutral Bi(iii)R_3_ moiety have mainly been reported to act as L-type or X-type ligands to transition metal centres utilising Bi(6s) or Bi(6p) orbitals, respectively (Fig. 1).^21,22^ Late transition metal complexes of an ambiphilic PBi(Cl)P ligand, however, were reported to exhibit dominant Z-type bonding caused by metal donation into a Bi–Cl(σ*)-antibonding orbital, highlighting the unique bonding properties of Bi derived ligands.^7,8^ Besides varying the substituents to manipulate the donor and acceptor properties of Bi, the donor capabilities of group 15 elements can further be tuned upon induction of geometric constraint.

Non-VSEPR geometries of PnR_3_ (Pn = Pnictogen) species have proven to unlock unique transformations such as (reversible) E–H bond scission, two-electron redox cycles and various small molecule activation reactions, even leading to efficient metal-free catalytic applications.^23–28^ Upon geometric perturbation of PnR_3_ species from tetrahedral *C*_3v_ symmetric structures towards bent or fully T-shaped (*C*_2v_) geometries, frontier molecular orbital rearrangement occurs, furnishing Lewis-acidic group 15 centres that feature vacant p-orbitals. The latter geometry necessitates rigid ligand platforms to enforce planarity rendering such species challenging to access, especially in the case of the lighter pnictogens. While various examples of geometrically constrained pnictogens have been reported over the last decade, studies on their properties as ligands remain scarce. Geometrically distorted phosphorus donor moieties were shown to display electrophilic behaviour, engage in P–M cooperative substrate activation reactions, P-centred catalysis as well as insertion into M–E bonds.^24,28–36^ To date, polydentate ligand platforms that feature strictly T-shaped group 15 centres remain elusive but hold large promise for innovative ligand design due to the unique orbital constitution present within these exotic species. In this context, Bi derived ligand platforms would be particularly suited to realise intriguing heavy metal–metal bonding interactions upon geometric distortion. Chitnis and co-workers showed that planarised trisamidobismuthanes exhibit Lewis-acidic reactivity, however, coupling with redox active supports was proposed to enable Bi(6p)→M L-type bonding due to electron delocalisation into the vacant Bi(6p) orbital originating from planarisation.^37–40^ These unique electronic features might allow to access Bi donor species that exhibit ambiphilic bonding properties towards transition metals centres which are both mediated *via* the partially occupied Bi(6p) orbital. While spectroscopic evidence and theoretical studies of such Bi complexes have been reported, structural authentication of this bonding mode, reminiscent of Bi(i) coordination chemistry, as well as its implementation into a pincer ligand, remains elusive.^41–47^ We aimed to construct an ambiphilic PBiP pincer ligand that features a T-shaped Bi donor functionality which itself is embedded within a redox non-innocent scaffold to realise such a bonding interaction. Recently, we reported the synthesis of rigid acridane-derived NNN pincer ligands that display pronounced redox non-innocence.^48,49^ Our synthetic approach extends this ligand platform by additional phosphine donor moieties to access a bespoke PBiP pincer ligand featuring a central T-shaped Bi trisamide. Instead of typical L-type bonding utilising the relativistically contracted Bi(6s) orbital, as commonly observed for neutral ligands featuring Bi–C bonds, Bi(6p)→M bonding should consequently be enforced *via* coupling planarised Bi with the redox non-innocent acridane scaffold.^21,50–54^

## Results and discussion

### Synthesis and characterisation of the ambiphilic PBiP pincer ligand

**Scheme 1:**
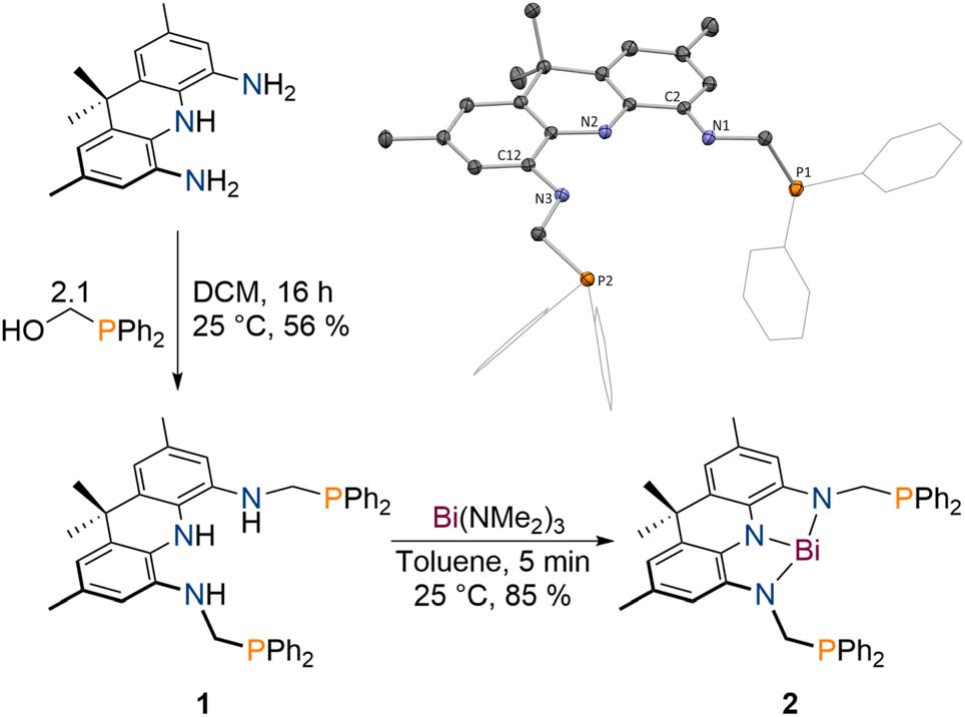
Synthesis of 1 upon reaction of 2,7,9,9-tetramethyl-9,10-dihydroacridine-4,5-diamine with diphenylphosphinomethanol and subsequent formation of 2 upon addition of Bi(NMe_2_)_3_ to produce 2; inset: molecular structure of 1 in the solid state obtained *via* SCXRD, ellipsoids at 50% probability level; H atoms omitted for clarity.

The protioligand 1 can be accessed upon addition of 2,7,9,9-tetramethyl-9,10-dihydroacridine-4,5-diamine^48^ to diphenyl-phosphinomethanol in dichloromethane (DCM) in moderate yields (Scheme 1). We developed a reliable procedure for the large-scale synthesis of PPh_2_(CH_2_OH) that affords a brittle white solid in excellent yield (91%, see ESI†). 1 displays overall *C*_2v_ symmetry on the NMR timescale indicated by the equivalency of the phosphorus atoms, the flanking amine groups as well as the methyl groups of the acridane unit (^31^P{^1^H} NMR: *δ* = −18.6 ppm). The molecular structure of 1 in the solid state obtained *via* single crystal X-ray diffraction (SCXRD)^55^ confirms the expected symmetric structure which features a planar acridane unit and displays almost identical bond metrics when compared to our recently reported acridane derived NNN ligands (Scheme 1).^48,49^

**Fig. 2:**
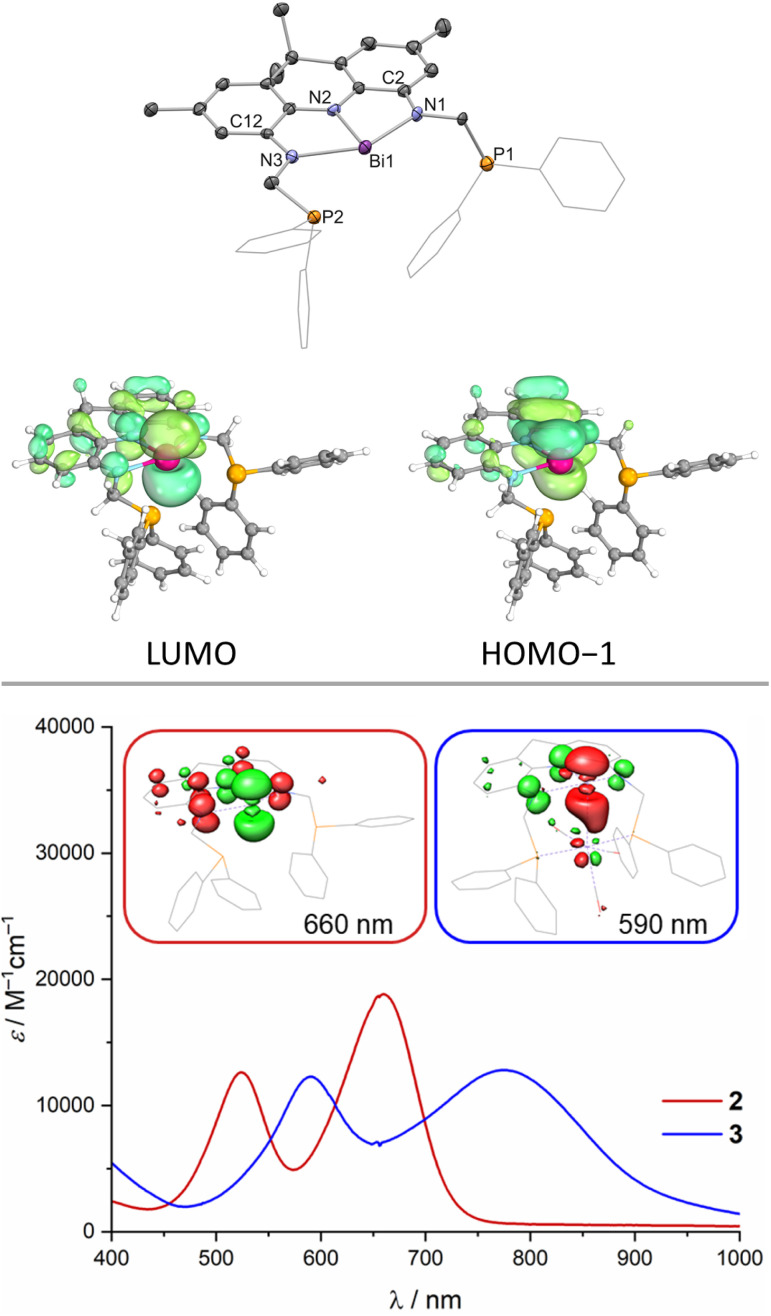
Molecular structure of 2 in the solid state obtained *via* SCXRD, ellipsoids at 50% probability level; H atoms are omitted for clarity, one molecule of the asymmetric unit shown and selected computed frontier molecular orbitals (top, Me groups of the acridane ligand truncated to H); UV/vis spectra of 2 and 3 (DCM, 25 °C, inset: calculated differences densities of selected transitions, depletion of electron density shown in red and accumulation in green).

1 was further analysed by IR spectroscopy and combustion analysis, both fully in line with the expected structure and composition of the protioligand. When 1 is reacted with Bi(NMe_2_)_3_ in toluene at room temperature, an instant colour change from colourless to deep purple is observed. The Bi trisamide 2 can be obtained in high isolated yields in form of a dark blue powder (Scheme 1). As observed for 1, 2 displays overall *C*_2v_ symmetry on the NMR timescale. Upon incorporation of Bi, the ^31^P{^1^H} NMR signal undergoes a minor low-field shift suggesting no significant interaction between the Bi centre and the phosphine donors (^31^P{^1^H} NMR: *δ* = −22.2 ppm). The resonances of the pincer arms' methylene protons further exhibit a significant downfield shift caused by Bi complexation (Δ*δ* = 2.27 ppm). SCXRD reveals formation of a strictly planar Bi trisamide in which the Bi centre adopts a slightly contracted T-shaped geometry (Fig. 2, N1–Bi1–N2: 71.29(13)°; N2–Bi1–N3: 72.03(13)°). Contraction of the C_2/12_–N_1/3_ bonds from 1.4133(14) and 1.4182(14) Å in 1 towards 1.353(5), 1.359(5), 1.365(5) and 1.355(5) Å upon Bi incorporation suggests an increased quinone character of the NNN ligand and by that depletion of electron density from the acridane ligand scaffold upon formation of 2 due to electron delocalisation into the vacant Bi(6p) orbital. This compares well with previous studies on tantalum(v) complexes of an acridane derived NNN pincer ligand that showed similar bond metrics upon ligand oxidation.^48^ The accurate description of the Bi redox state in geometrically constrained trisamides featuring redox active substituents has been debated and ligand mediated reduction towards Bi(i) has been proposed, while a recent report by Andrada, Salvador and co-workers points towards a more accurate description as Bi(iii) featuring delocalisation of electron density from the redox active substituents into the vacant Bi(6p) orbital to allow for Lewis basic reactivity to occur.^38,41^ The computed molecular orbitals of 2 indicate highly covalent bonding between the Bi centre and the NNN scaffold.

While the HOMO is mainly based on the NNN backbone, the HOMO−1 features significant Bi(6p) orbital contribution with in-phase bonding towards the occupied NNN scaffold's central N(2p) orbital (Fig. 2). The LUMO is represented by the antibonding combination. A high degree of covalency in the bonding between the Bi centre and the NNN chelate is further evidenced by Complete Active Space Self Consistent Field (CASSCF) calculations that reveal the presence of a 2c–2e π bond between Bi and the central N of the NNN pincer with very minor polarisation towards nitrogen and an occupation of the Bi(6p) orbital with 0.91 e^−^ thus preventing the unequivocal assignment of a Bi(iii) or Bi(i) redox state for 2. Time-dependent DFT calculations reveal that the intense purple colour of 2 can be described as HOMO−1 → LUMO and HOMO → LUMO excitations associated with bands at *λ* = 524 nm and 660 nm, respectively, observed in the UV/vis spectrum in DCM at room temperature, in line with prior reports on T-shaped pnictogen species (Fig. 2, see ESI†).^38,56^ Tan, Ruan, Wang an co-workers recently reported the successful isolation of T-shaped P, As and Sb radical anions upon one-electron reduction of the respective T-shaped trisamides.^56^ The cyclic voltammogram of 2 features a quasi-reversible reductive event at *E*_1/2_ = −1.98 V *vs.* Fc^0/+^ that becomes increasingly irreversible at lower scan rates (see ESI†). This points towards the possible isolation of T-shaped Bi radical anions when sterically more demanding ligand scaffolds are employed. Furthermore, ill-defined oxidative events at *E* ⪆ 0 V *vs.* Fc^0/+^ accompanied by immediate fouling of the glassy carbon working electrode and deposition of insoluble black material on the electrode surface indicate disintegration of the molecular structure upon oxidation (see ESI†). The Lewis-acidity of 2 was assessed *via* the Gutmann–Beckett method upon comparing ^31^P NMR shift differences of free Et_3_PO with an equimolar mixture of 2 and Et_3_PO.^57,58^ An acceptor number (AN) of ≤20 was obtained in dichloromethane which represents an upper limit due to the intrinsic Lewis-acidity of the employed solvent (AN_DCM_ = 20.4). Lichtenberg and co-workers introduced an alternative scale for soft Bi Lewis-acids upon comparing the ^31^P NMR shift of free Me_3_PS against 1 : 1 adducts with the respective Lewis-acid in dichloromethane.^59^2 features a low acceptor number of 8, comparable with BiPh_3_ and in line with results obtained for similar systems reported by Chitnis and co-workers.^37^ The calculated Fluoride Ion Affinity (FIA) of 61 kcal mol^−1^ supports the notion of 2 being a very weak Lewis-acid.^60^

**Fig. 3:**
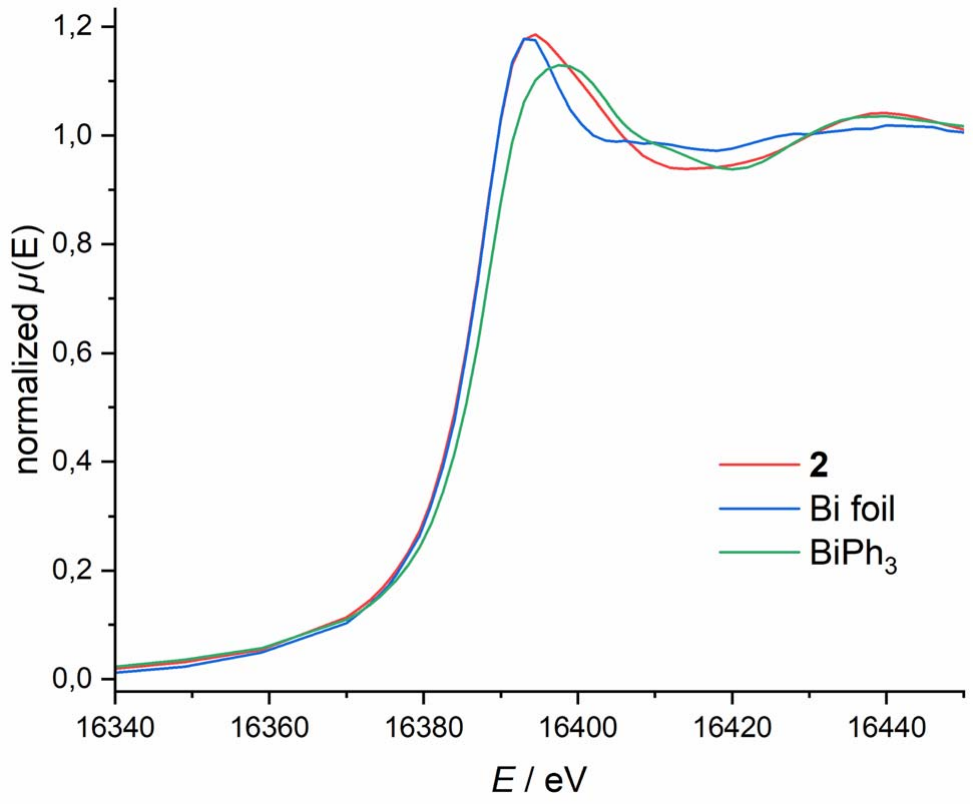
Bi L_1_-edge XANES spectra of 2, Bi foil and BiPh_3_.

To further elucidate the bonding situation, we probed the Bi oxidation state *via* X-ray Absorption Near Edge Structure (XANES) spectroscopy. Bi L_1_-edge X-ray absorption spectra of 2, BiPh_3_ and Bi foil are depicted in Fig. 3. 2 features a low energy edge position (16 388.6 eV), lower than the classical Bi(iii) compound BiPh_3_ (16 387.9 eV), and closer to metallic Bi (16 388.0 eV). This becomes even more pronounced when comparing the whiteline shape and energy position. For 2 this is at 16 394.0 eV, considerably lower than the one observed for BiPh_3_ (16 397.9 eV) and closer to metallic Bi foil (16 393.5 eV). The same trend can be observed for the Bi L_3_-edge (see ESI†). These spectroscopic results, in combination with our theoretical studies, demonstrate the highly covalent bonding between the Bi centre and the NNN scaffold that results in partial occupation of the Bi(6p) orbital, quenching its expected Lewis-acidity stemming from planarisation and complicating unequivocal assignment of a Bi redox state. Consequently, subsequent reactivity studies are required to obtain a more comprehensive understanding of 2's donor/acceptor properties stemming from this unusual electronic structure, in particular its capability to form transition metal complexes displaying ambiphilic bonding interactions.

### Complexation of transition metal centres by 2

**Fig. 4:**
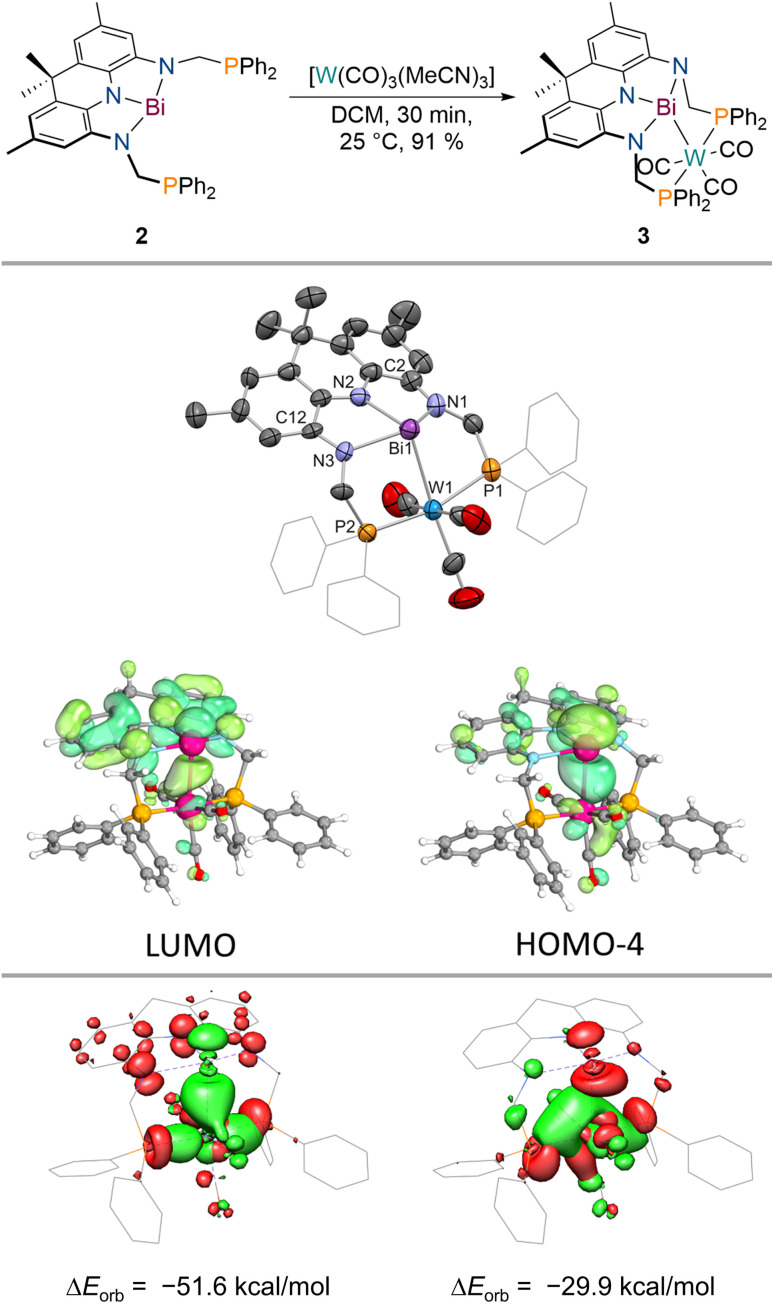
Synthesis of 3 upon addition of [W(CO)_3_(MeCN)_3_] to 2 (top); molecular structure of 3 in the solid state obtained *via* SCXRD, ellipsoids at 50% probability level; H atoms are omitted for clarity, one molecule of the asymmetric unit shown; and selected computed frontier molecular orbitals (middle, Me groups of the acridane ligand truncated to H); selected donor (red)/acceptor (green) orbital pairs derived *via* energy decomposition analysis showcasing the ligand's redox activity as well as the ambiphilic bonding between tungsten and bismuth (bottom).

When 2 is reacted with [W(CO)_3_(MeCN)_3_] in DCM, an instant colour change from dark purple towards dark blue is observed. A single new signal is detected in the ^31^P{^1^H} NMR spectrum at *δ* = 42.3 ppm which features ^183^W satellites (^1^*J*_P–W_ = 288 Hz) suggesting incorporation of tungsten into the PBiP pincer. The ^1^H NMR spectrum features significantly broadened resonances except for the equatorial benzylic and the aromatic protons of the acridane moiety. Cooling the sample to lower temperatures (−60 °C), however, results in sharpening of all signals and an overall *C*_s_ symmetry on the NMR timescale is revealed which indicates that the tungsten centre binds to the ligand *via* the Bi(6p) orbital (see ESI†). The structure of the formed complex could be clarified *via* SCXRD (Fig. 4), IR spectroscopy and combustion analysis confirming the successful incorporation of a W(CO)_3_ fragment into the PBiP pincer ligand in near quantitative yield (3, Fig. 4). Comparing the carbonyl stretching frequencies of 3 with values reported for structurally related tungsten tricarbonyl PNP pincer complexes that feature a meridional coordination mode and flanking PPh_2_ donor moieties reveals that the Bi centre's donor capabilities are comparable to pyridine.^61,62^3 slowly degrades in solution and in the solid state at room temperature but can be stored for weeks at −30 °C in the dark. The Bi centre slightly bends out of the NNN ligand's plane accompanied by a further shortening of the C_2/12_–N_1/3_ bonds (*ca.* 0.02 Å) within the ligand when compared to 2, thus demonstrating further depletion of electron density from the acridane support to facilitate metal binding. The Bi–W bond length (2.8059(9) and 2.8049(5) Å) is slightly shorter compared to a recently reported Bi(i)–W(CO)_5_ complex (2.933(7) and 2.944(6) Å) or Ph3Bi–W(CO)5 (2.829(3) Å), likely owing due to the chelating nature of the employed ligand scaffold.^46,63^ The observed dynamic behaviour in solution is attributed to a rocking motion of the phosphine ligated W centre around the Bi(NNN) plane. This is supported by theoretical calculations which predict a low barrier for this process (Δ*G*^‡^_DFT_ = 12 kcal mol^−1^) in line with observed broadened NMR signals at room temperature that sharpen upon cooling (see ESI†). The acceptor numbers of 3*versus* Et_3_PO and Me_3_PS are identical to 2 suggesting only a minor influence of metal incorporation on the Bi Lewis-acidity as further supported by a calculated FIA of 56 kcal mol^−1^. In contrast to 2, 3 features well-defined redox events in the cyclic voltammogram (see ESI†). An irreversible oxidative event (*v* = 100–800 mV s^−1^) is observed in dichloromethane at *E* ≈ −0.2 V *vs.* Fc^0/+^ likely due to metal decarbonylation upon one-electron oxidation. A quasi-reversible reduction is detected at *E*_1/2_ = −1.39 V *vs.* Fc^0/+^. The reaction of 3 with decamethylcobaltocene in dichloromethane, however, resulted in unselective decomposition. To elucidate the bonding between W and Bi a number of theoretical techniques were utilised. Distinct L-type binding of the W centre by the central Bi donor is suggested by the calculated frontier molecular orbitals. In line with the contraction observed in the C_2/12_–N_1/3_ bonds with regards to 2, the HOMO−4 of 3 clearly displays Bi(6p)→W donation while the LUMO is mainly located on the NNN backbone in contrast to 2 (Fig. 4). 3 features two intense transitions in the UV/vis spectrum at *λ* = 590 nm and 774 nm (Fig. 3).

Calculation of the difference densities shows that these transitions are drastically different in nature when compared to 2. While the low-energy transition stems from an acridane π–π* transition, the other band can be interpreted as a Bi(6p) → acridane(π*) electron transfer, in full agreement with the proposed population of the formally vacant Bi(6p) orbital. The calculated Electron Localization Function (ELF; see ESI†)^64,65^ showcases significant accumulation of electron density along the Bi–W bond complemented by a calculated Mayer bond order of 0.59 which compares well with values obtained for Bi(i)–W(CO)_5_ complexes showcasing the successful mimicry of typical Bi(i) coordination behaviour enabled by coupling of Bi planarisation and the redox non-innocence of the NNN scaffold.^46^ The characteristic energy and electron densities at the bond critical point derived *via* Quantum Theory of Atoms in Molecules (QTAIM)^66^ calculations are in full agreement with a dative metal–metal interaction (*ρ*(*r*) = 0.044; ∇^2^*ρ*(*r*) = 0.052; *H*(*r*) = −0.011; see ESI†).^67^ Energy Decomposition Analysis (EDA)^68^ between the PBiP and W(CO)_3_ fragments further reveals the unique ambiphilic nature of the PBiP pincer ligand system (Fig. 4). Bi(6p)→W as well as W→Bi(6p) donor/acceptor interactions can be unequivocally deduced while Bi(6s)→W L-type bonding could not be identified (see ESI†). Consequently, the implementation of Bi within a rigid, redox non-innocent supports allows for the realisation of ambiphilic Bi–W donor/acceptor interactions in which both interactions are mediated by the Bi(6p) orbital due the highly covalent bonding within the Bi(NNN) unit. This contrasts with prior reports on related systems which facilitate ambiphilic Bi–M bonding *via* two different orbitals, *e.g.*, Bi(6s) and Bi–Cl(σ*) orbitals in PBiP pincer ligands that are not redox non-innocent.^7,8^

**Fig. 5:**
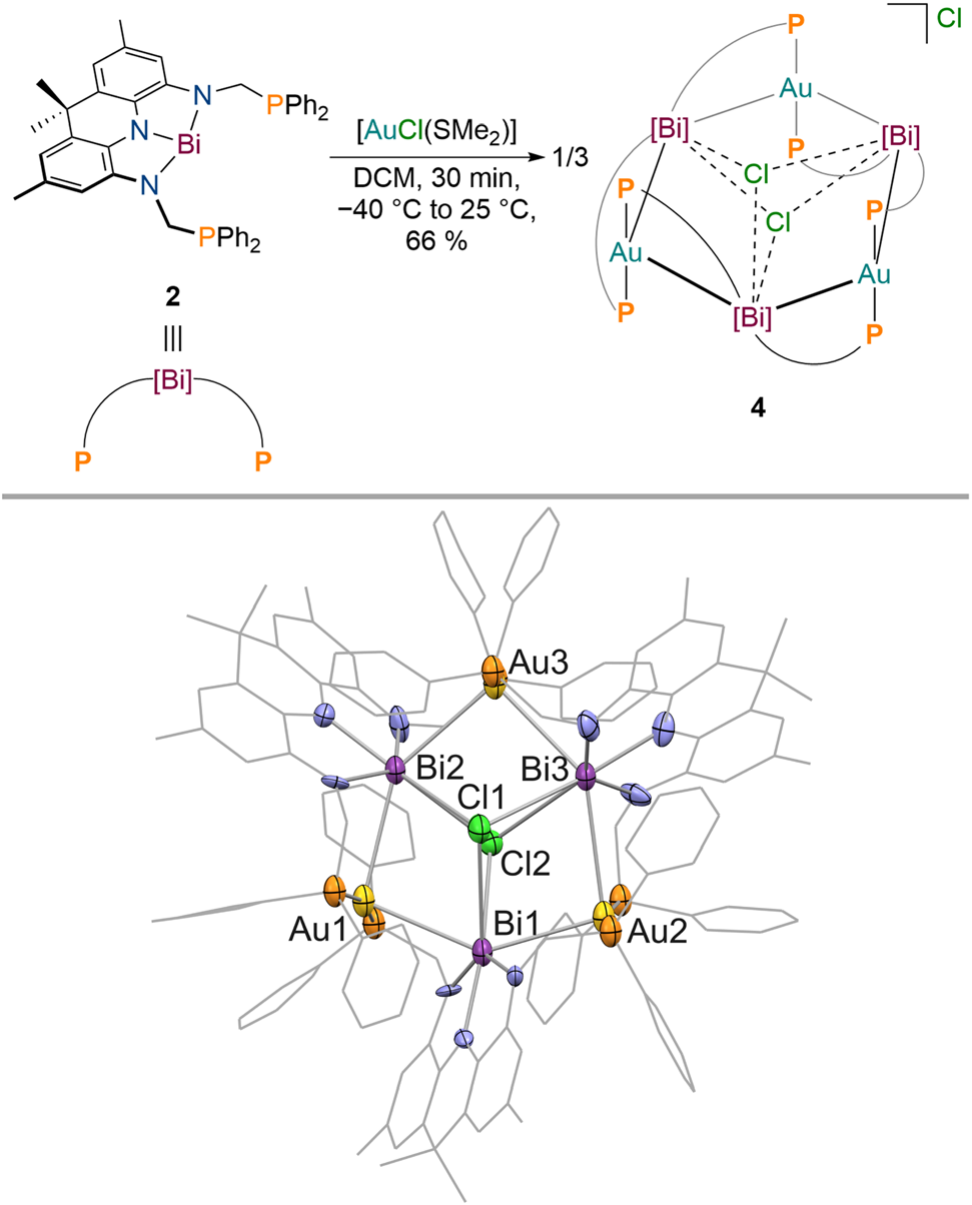
Synthesis of 4 upon addition of [AuCl(SMe_2_)] to 2 (top); molecular structure of 4 in the solid state obtained *via* SCXRD, ellipsoids at 50% probability level; H atoms, the chloride anion and solvent molecules are omitted for clarity (bottom).

Next, we aimed to investigate the bonding of 2 towards an electron-rich d^10^ metal centre to clarify if the donor properties of 2 are dependent on the Lewis-acidity and electron configuration of the utilised metal. Gold complexes of Bi(iii) derived ligands are still extremely scarce since transmetallation instead of coordination can easily occur, necessitating tailored substituents at Bi to prevent undesired side reactions (Fig. 1).^6–8,69,70^ When 2 is reacted with [AuCl(SMe_2_)] in DCM at −30 °C, a colour change towards turquoise (intense absorption at *λ* = 744 nm) is observed accompanied by the quantitative formation of a new signal in the ^31^P{^1^H} NMR spectrum at *δ* = 59.3 ppm. The isolated product features significantly reduced signal broadening in contrast to 3 in the ^1^H NMR spectrum at room temperature. While the methyl groups of the acridane pincer scaffold are magnetically equivalent on the NMR timescale, the methylene protons of the phosphine donor arms display geminal coupling (^2^*J*_H–H_ = 12 Hz, Δ*δ* = 1.53 ppm) pointing towards an overall *C*_2v_ symmetry of the reaction product in solution in which complexation of two gold centres by 2 occurs. This is further corroborated by diffusion ordered NMR spectroscopy (DOSY-NMR) which reveals that the formed metal complex is significantly larger in solution than 1–3 (1.7 times the size of 3, see Table S1†). SCXRD revealed that a multi-metallic cluster has been formed (Fig. 5).

**Fig. 6:**
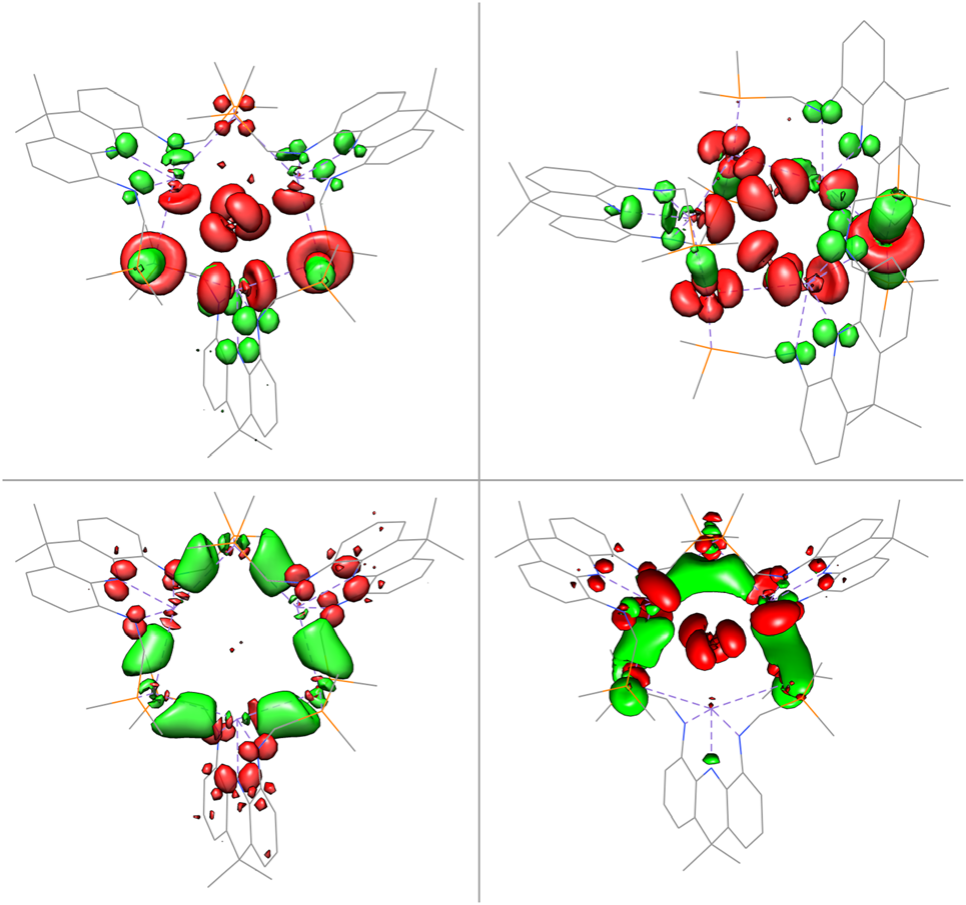
Selected donor and acceptor orbital pairs derived by EDA of 4 (PPh_2_ truncated to PMe_2_ and equatorial acridane methyl groups to H; donor orbitals in red and acceptor orbitals in green).

The gold centres are linearly coordinated by two phosphine donors stemming from different PBiP ligands and additionally feature Bi–Au interactions resulting in the formation of a six-membered Au_3_Bi_3_ ring that is capped by two chlorides to give the monocationic complex 4 (Fig. 5). 4 is stable for hours at room temperature in DCM but immediately decomposes when exposed to strongly donating or aromatic solvents such as tetrahydrofuran, toluene, benzene or chlorobenzene. The molecular structure of 4 shows that all Bi–Cl and Bi–Au bond distances are below the sum of their respective van der Waals radii suggesting the presence of Bi–Au orbital interactions. This is corroborated by the calculated Mayer bond orders that indicate Bi–Au (0.24) and Bi–Cl (0.14) bonding to be present, while Bi–Bi, Au–Au and Au–Cl bonding is negligible (all below 0.1). The bonding within the cluster was further analysed *via* EDA calculations, since inspection of the frontier molecular orbitals revealed little information due to the size and delocalisation of the studied system. As for 3, the phosphine–gold interactions are dominant. In addition, electron flow between the redox non-innocent ligand towards the Bi–Au bonds as well as gold stabilisation *via* the ambiphilic Bi(6p) orbital is evident, albeit less pronounced when compared to 3 (Fig. 6). Bi–Cl bonding interactions indicate an essential role of the capping chlorides to stabilise the cluster (see ESI†).

This showcases that 2 is a competent ligand for early as well as late transition metals and allows not only to form discrete pincer complexes with unique bonding characteristics but also large multi-metallic clusters due to its flexible phosphine side-arms. In both of these structure types ambiphilic Bi–M binding is realised originating from a coupling of Bi planarisation and employment of a redox-active support.

## Conclusions

In conclusion, we report the first synthesis of a PBiP pincer ligand in which transition metals engage in rare ambiphilic Bi(6p)–M bonding enabled by a combination of geometric constraint on the Bi centre and a redox active NNN scaffold. The highly covalent bonding between these two fragments allows Bi to act as an electron relay for the NNN chelate when bound to transition metals resulting in the formation of pincer complexes as well as multi-metallic clusters. Future study will target to exploit these unique bonding characteristics in the context of small molecule activation reactions *via* heavy atom – transition metal cooperativity and the controlled construction of larger cluster compounds.

## Data availability

Synthetic procedures, spectroscopic results and details as well as computational details can be found in the ESI.† Crystallographic datasets have been deposited at the CCDC under 2320292, 2320297 and 2320304.

## Author contributions

P. C. performed the theoretical analysis of all presented compounds. A. G. B. performed the XANES measurements and interpreted the data under the supervision of F. E. J. A. conceptualised the project, performed the synthetic work, interpreted the spectroscopic results, performed the SCXRD studies and wrote the original draft of the manuscript. The final version was edited and approved by all co-authors.

## Conflicts of interest

There are no conflicts to declare.

## Supplementary Material

SC-015-D4SC00197D-s001

SC-015-D4SC00197D-s002
