# Supplementary material for: Combining geometric constraint and redox non-innocence within an ambiphilic PBiP pincer ligand
Source: Chem Sci. 2024 Mar 18;15(16):6036–43. doi: 10.1039/d4sc00197d (PMC11040644; doi:10.1039/d4sc00197d)
Supplement: SC-015-D4SC00197D-s001 [file SC-015-D4SC00197D-s001.pdf]

Electronic Supplementary Material (ESI) for Chemical Science.
This journal is © The Royal Society of Chemistry 2024

Table of Contents

Materials and Methods S2

Synthetic Procedures S3

Spectroscopic Data S5

Cyclic voltammetry S23

Single Crystal X-ray Diffraction S26

Crystallographic Details of 1 .... S26

Crystallographic Details of 2 .... S30

Crystallographic Details of 3 .... S36

Crystallographic Details of 4 .... S43

X-ray Absorption Near Edge Structure (XANES) spectroscopy S51

Computational Details S53

XYZ-coordinates S54

Computational Analysis of 2 .... S66

Computational Analysis of 3 .... S69

Computational Analysis of 4 .... S74

References S76

Materials and Methods

All experiments with air-sensitive compounds were carried out in a glovebox or in a fume hood employing Schlenk techniques under a dry Ar atmosphere. Traces of water and oxygen were removed via heating of glassware under vacuum prior to use.

All solvents except THF were dried and degassed by a MBraun solvent purification system. THF was dried over sodium, distilled and stored over molecular sieves. Deuterated solvents were degassed via three freeze-pump-thaw cycles and stored over molecular sieves.

NMR spectra were recorded on AVANCE II 300 MHz, Bruker Avance 400 MHz and Bruker Avance III 500 MHz NMR spectrometers except for DOSY NMR experiments which were recorded on a Bruker AVANCE 600 MHz NMR. Chemical shifts are referenced to the signal of residual protonated solvent.

IR spectra were recorded on a Bruker ALPHA spectrometer with an ATR sampling unit.

Elemental analyses were performed with a HEKA Euro 3000EA elemental analyzer.

UV-vis-spectra were measured in a 1 cm quartz cuvette using an Agilent 8453 UV-vis-spectrophotometer with an Unisoku USP-203-A cryostat. Fluorescence spectroscopy was performed on a Cary Eclipse in 1 cm quartz cuvettes.

Cyclic voltammetry was performed in a glovebox with a PalmSens Emstat3$^+$ Blue potentiostat using a glassy carbon working electrode, a Pt wire as counter electrode and an Ag wire as pseudo-reference electrode.

All commercially available chemicals were used without purification unless otherwise noted. $BiCl_3$ which was sublimed at 260 °C in the dark. Ferrocene was sublimed twice and $[NBu_4]PF_6$ was dried at 120 °C for 2 days before use. 2,7,9,9-tetramethyl-4,5-dinitro-9,10-dihydroacridine[48] and $Bi(NMe_2)_3$[S1] were synthesized according to literature procedures.

Synthetic Procedures

Synthesis of diphenylphosphinomethanol: Paraformaldehyde (1.00 g, 33.0 mmol, 1.00 eq.) and $HPPh_2$ (6.03 mL, 34.6 mmol, 1.05 eq.) are placed in a J-Young ampoule and heated to 100 °C for 3 h. After cooling to room temperature, hexane (10 mL) is added and the reaction mixture is sonicated for 5 minutes. The organic layer is decanted and the procedure is repeated once. The oily residue is dissolved in benzene (5 mL) and filtered. Lyophilization out of benzene yields diphenylphosphinomethanol (6.50 g, 30.1 mmol, 91 %) as a brittle white solid.

NMR: (C_6D_6, 25 °C): ^1H (300 MHz): δ (ppm) = 7.49 – 7.43 (m, 4H, $C_{ortho}H$), 7.10 – 7.01 (m, 6H, $C_{meta/para}H$), 4.17 (dd, $^2J_{HP}$ = 8.2 Hz, $^3J_{HH}$ = 5.2 Hz, CH_2), 1.60 – 1.55 (br, 1H, O*H*); $^{13}C\{^1H\}$ (75.5 MHz): δ (ppm) = 138.4 (d, $^1J_{CP}$ = 13.0 Hz, 2C, C_{ipso}), 135.2 (d, $^2J_{CP}$ = 17.7 Hz, 4C, C_{ortho}), 130.6 (s, 2C, C_{para}), 130.5 (d, $^3J_{CP}$ = 6.4 Hz, 4C, C_{meta}); $^{31}P\{^1H\}$ (121 MHz): δ (ppm) = –11.1 (s, 1P).

IR: (ATR, solid, 25 °C): ν (cm^{-1}) = 3374 (br), 1431 (m), 1007 (s), 736 (s), 689 (s).

Elem. Anal. found (calcd) for $C_{13}H_{13}OP$: C, 72.2 (72.2); H, 6.20 (6.06).

New large-scale synthesis of 2,7,9,9-tetramethyl-9,10-dihydroacridine-4,5-diamine: 2,7,9,9-tetramethyl-4,5-dinitro-9,10-dihydroacridine (1.82 g, 5.56 mmol, 1.00 eq.) and Pd/C (400 mg, 10 w%, 0.38 mmol, 0.07 eq.) were suspended in EtOAc (10 mL) and the mixture was degassed via three freeze-pump-thaw cycles. The flask was backfilled with H_2 (1 atm) and stirred at 50 °C for 48 h. After filtration, the solvent was removed and the yellow residue was dissolved in DCM (5 mL) and layered with hexane (30 mL). Storing the flask at -30 °C for 3 days resulted in the formation of white crystalline needles that were isolated via filtration and washed twice with hexane (5 mL). 2,7,9,9-tetramethyl-9,10-dihydroacridine-4,5-diamine (1.23 mg, 4.60 mmol, 82%) was isolated as a white powder. The NMR data matched the prior reported one.[48]

Elem. Anal. found (calcd) for $C_{17}H_{21}N_3$ (267.4): C, 75.8 (76.4); H, 7.99 (7.92); N, 15.4 (15.7).

Synthesis of 1: 2,7,9,9-tetramethyl-9,10-dihydroacridine-4,5-diamine (500 mg, 1.87 mmol, 1.00 eq.) and diphenylphosphinomethanol (849 mg, 3.92 mmol, 2.10 eq.) are dissolved in DCM (10 mL) and stirred at 25 °C for 16 h. The solvent is removed, the residue is washed with cold Et_2O (2 x 5 mL) and dried overnight. **I** (721 mg, 1.09 mmol, 58%) is obtained as a white powder.

NMR: (C_6D_6, 25 °C): ^1H (500 MHz): δ (ppm) = 7.84 – 7.44 (m, 8H, PPh_2), 7.09 – 7.02 (m, 12H, PPh_2), 6.94 (d, $^4J_{HH}$ = 1.7 Hz, 2H, $C_{aryl}H$), 6.53 (d, $^4J_{HH}$ = 1.7 Hz, 2H, $C_{aryl}H$), 6.13 (s, 2H, N*H*), 3.71 (dd, $^3J_{HH}$ = 6.2 Hz. $^3J_{HP}$ = 3.0 Hz, NCH_2P), 3.16 (bt, $^3J_{HH}$ = 6.2 Hz, 1H N*H*), 2.30 (s, 6H, $C_{aryl}Me$), 1.67 (s, 6H, $C_{aryl}Me$); $^{13}C\{^1H\}$ (125.7 MHz) δ (ppm) = 137.9 – 137.8 (4C), 135.7 – 135.6 (2C), 133.4 – 132.3 (8C), 129.9 – 129.8 (4C), 129.0 – 128.9 (12C), 128.4 (2C), 118.9 (2C, $C_{aryl}H$), 114.4 (2C, $C_{aryl}H$), 46.3 (d, $^1J_{CP}$ = 7.6 Hz, 2C, NCH_2P), 36.6 (s, 1C, CMe_2), 32.3 (s, 2C, $C_{aryl}Me$), 21.8 (s, 2C, CMe_2); $^{31}P\{^1H\}$ (121 MHz) δ (ppm) = –18.6 (s, 2P, PPh_2).

IR: (ATR, solid, 25 °C): ν (cm^{-1}) = 3322 (br), 1500 (s), 1433 (s), 738 (s), 693 (s).

Elem. Anal. found (calcd) for $C_{43}H_{43}N_3P_2$ (216.2): C, 77.2 (77.8); H, 6.66 (6.53); N, 6.23 (6.33).

Synthesis of 2: **1** (317 mg, 447 µmol, 1.00 eq.) is dissolved in toluene (5 mL) and $Bi(NMe_2)_3$ (163 mg, 447 µmol, 1.00 eq.) is added under exclusion of light. The solution is stirred at 25 °C for 30 min and the solvent is removed. The residue is washed with hexane (2 x 10 mL) and extracted with benzene (2 x 4 mL). Lyophilization out of benzene yields **2** (330 mg, 379 µmol, 85%) as a dark blue powder.

NMR: (C_6D_6, 25 °C): ^1H (500 MHz) δ = 7.42 – 7.38 (m, 8H, PPh_2), 7.10 (br, 2H, $C_{3/6}H$), 7.04 – 6.98 (m, 12H, PPh_2), 6.73 (d, $^4J_{H–H}$ = 1.5 Hz, 2H, $C_{1/8}H$), 5.98 (d, $^2J_{H–P}$ = 2.5 Hz, 4H, NCH_2P), 2.85 (s, 6H, $C_{2/7}Me$), 1.80 ppm (s. 6H, C_9Me_2); $^{13}C\{^1H\}$ (125.8 MHz) δ = 155.2 (d, $^3J_{C–P}$ = 8.6 Hz, 2C, $C_{4/5}$), 139.8 (s, 2C, $C_{2/7}$), 139.0 (d, $^1J_{C–P}$ = 13.6 Hz, 4C, C_{Ph}), 138.1 (s, 2C), 134.7 (s, 2C), 133.3 (d, $^2J_{C–P}$ = 17.4 Hz, 8C, C_{Ph}), 129.1 (s, 4C, C_{Ph}), 128.9 (d, $^3J_{C–P}$ = 6.5 Hz, 8C, C_{Ph}), 115.6 (s, 2C, $C_{3/6}$), 111.4 (s, 2C, $C_{1/8}$), 53.6 (d, $^1J_{C–P}$ = 4.2 Hz, 2C, NCH_2P), 37.9 (s, 1C, C_9), 36.6 (s, 2C, C_9Me_2), 21.2 ppm (s, 2C, $C_{2/7}Me$); $^{31}P\{^1H\}$ (202.5 MHz) δ = –22.2 ppm (br, 2P, PPh_2).

IR: (ATR, solid, 25 °C): ν (cm^{-1}) = 1595 (m), 1430 (m), 1292 (s), 738 (m), 693 (m).

UV/vis: (DCM; $5*10^{-5}$ M, 25 °C): λ_{max} (nm) = 524, 660.

Elem. Anal. found (calcd) for $C_{43}H_{40}BiN_3P_2$ (869.7): C, 60.0 (59.4); H, 4.83 (4.64); N, 4.73 (4.83).

Synthesis of 3: **2** (145 mg, 166 µmol, 1.00 eq.) is dissolved in DCM (5 mL) and $W(CO)_3(MeCN)_3$ (65.0 mg, 166 µmol, 1.00 eq) is added in one portion. After stirring the reaction mixture for 30 minutes at room temperature in the dark, the solvent is removed and the residue is washed with hexane (2 x 5 mL). Lyophilization out of benzene yields **3** (172 mg, 151 µmol, 91%) as a dark blue powder.

NMR: (DCM, 25 °C): ^{1}H (400 MHz) δ = 6.71 (s br, 2H, $C_{3/6}$*H*), 6.52 (s br, $C_{1/8}$*H*), 5.80 (br, 2H, NC*H*$_2$P), 5.55 (br, 2H, NC*H*$_2$P), 2.50 (s, 6H, $C_{2/7}$*Me*); Acquisition of ^{13}C{^{1}H} NMR data was hindered by significant broadening of the resonances likely caused by slow molecular motion of the cluster in solution; ^{31}P{^{1}H} (161.9 MHz) δ = 42.3 ppm (s, $^1J_{P–W}$ = 288 Hz, 2P, *P*Ph$_2$); NMR (DCM, –60 °C): ^{1}H (400 MHz) δ = 7.77 – 7.73 (m, 4H, P*Ph$_2$*), 7.53 – 7.41 (m, 6H, P*Ph$_2$*), 7.16 – 7.09 (m, 6H, P*Ph$_2$*), 7.03 – 6.99 (m, 4H, P*Ph$_2$*), 6.73 (s br, 2H, $C_{3/6}$*H*), 6.48 (s br, $C_{1/8}$*H*), 6.02 (br, $^2J_{H–H}$ = 14.6 Hz, 2H, NC*H*$_2$P), 5.55 (br, $^2J_{H–H}$ = 14.6 Hz), 2.52 (s, 6H, $C_{2/7}$*Me*), 1.79 (s, 3H, C_9*Me*), 1.20 (s, 3H, C_9*Me*).

IR: (ATR, solid, 25 °C): ν (cm^{-1}) = 1846 (s, CO), 1826 (s, CO), 1431 (w), 1281 (m), 694 (m).

UV/vis: (DCM; 5*10$^{–5}$ M, 25 °C): λ_{max} (nm) = 590, 774.

Elem. Anal. found (calcd) for $C_{46}H_{40}BiN_3O_3P_2W$ (1137.6): C, 49.1 (48.6); H, 3.54 (3.51); 3.85 (3.69).

Synthesis of 4: **2** (51.5 mg, 59.2 µmol, 1.00 eq.) is dissolved in DCM (2 mL) and cooled to -35 °C. AuCl(SMe$_2$) (17.4 mg, 59.2 µmol, 1.00 eq.) is added in one portion under the exclusion of light and the solution is stirred for 30 minutes while warming to room temperature. The reaction mixture is filtered, layered with Et_2O (8 mL) and stored at-30 °C for 3 d. The dark blueish green precipitate is decanted and the residue is washed with Et_2O (2 x 2 mL). After drying, **4** (43.0 mg, 39.0 µmol, 66%) is obtained as a dark blue microcrystalline powder.

NMR: (C_6D_6, 25 °C): ^{1}H (500 MHz) δ = 7.61 (d, $^2J_{H–H}$ = 12.0 Hz, 2H, NC*H*$_2$P), 7.18 – 6.89 (m, 6H, P*Ph$_2$*), 7.03 – 6.89 (m, 14H, P*Ph$_2$*), 6.28 (s br, 2H, $C_{3/6}$*H*), 6.08 (d, $^2J_{H–H}$ = 12.0 Hz, 2H, NC*H*$_2$P), 5.56 (s br, 2H, $C_{1/8}$*H*), 2.39 (s, 6H, $C_{2/7}$*Me*), 1.69 (s, 6H, C_9*Me$_2$*). Acquisition of ^{13}C{^{1}H} NMR data was hindered by significant broadening of the resonances likely caused by slow molecular motion of the cluster in solution. ^{31}P{^{1}H} (202.5 MHz) δ = 59.3 ppm (br, 2P, *P*Ph$_2$).

IR: (ATR, solid, 25 °C): ν (cm^{-1}) = 2916 (m), 1598 (m), 1433 (m), 1284 (s), 689 (m).

UV/vis: (DCM; 5*10$^{–5}$ M, 25 °C): λ_{max} (nm) = 744.

Elem. Anal. found (calcd) for $C_{129}H_{120}Au_3Bi_3Cl_3N_9P_6$ (3306.5): C, 46.8 (46.9); H, 3.60 (3.66); N, 3.79 (3.81).

Spectroscopic Data

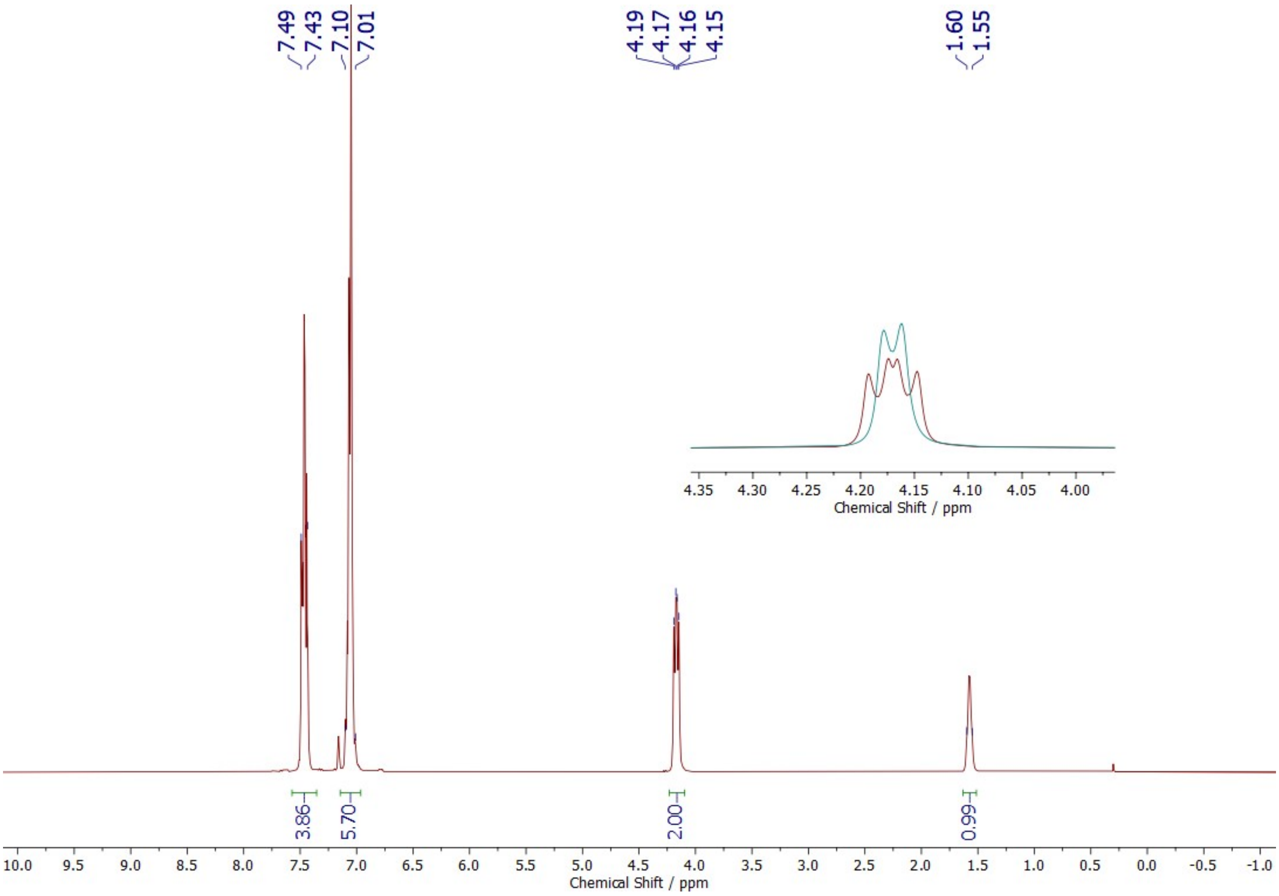

Figure S1. ^{1}H NMR spectrum of diphenylphosphinomethanol, C_6D_6, 25 °C; Inset: ^{1}H (red) and $^{1}H\{^{31}P\}$ (teal) NMR spectrum.

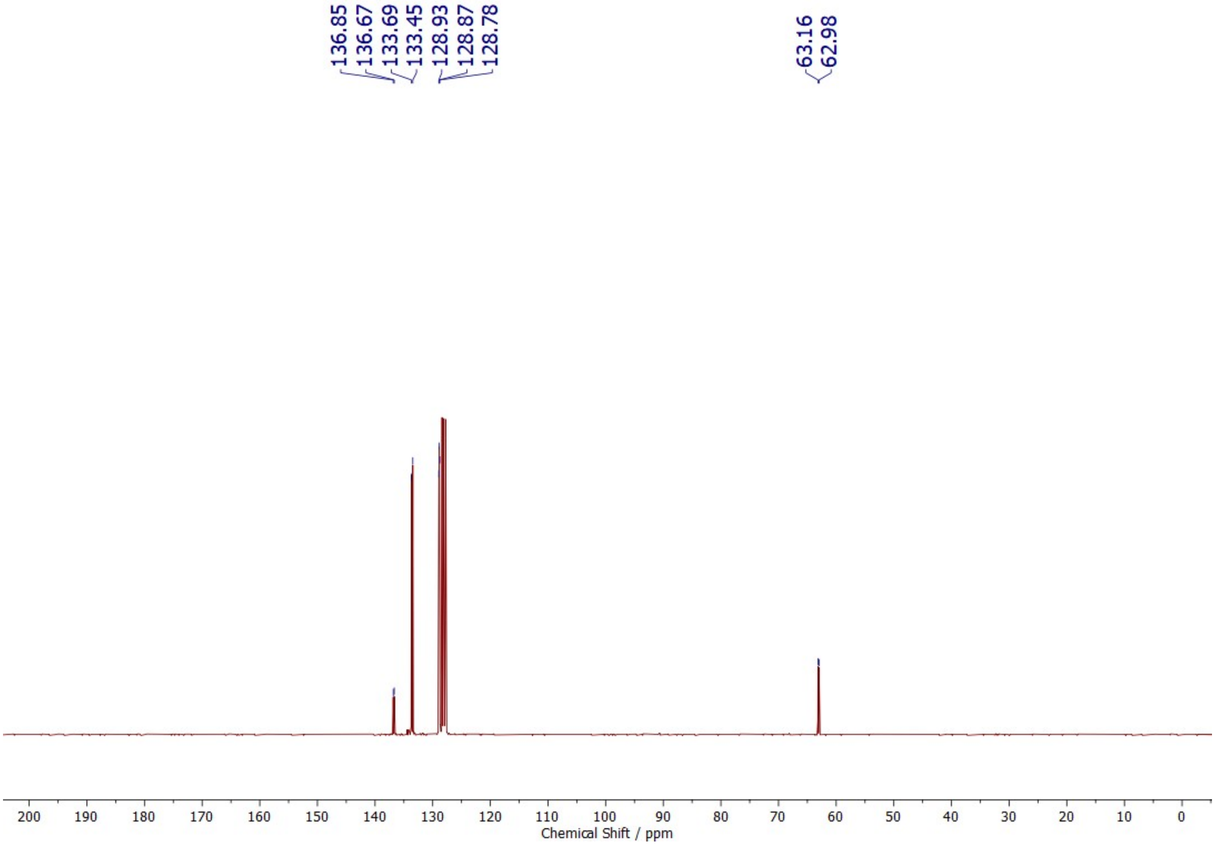

Figure S2. $^{13}C\{^{1}H\}$ NMR spectrum of diphenylphosphinomethanol, C_6D_6, 25 °C.

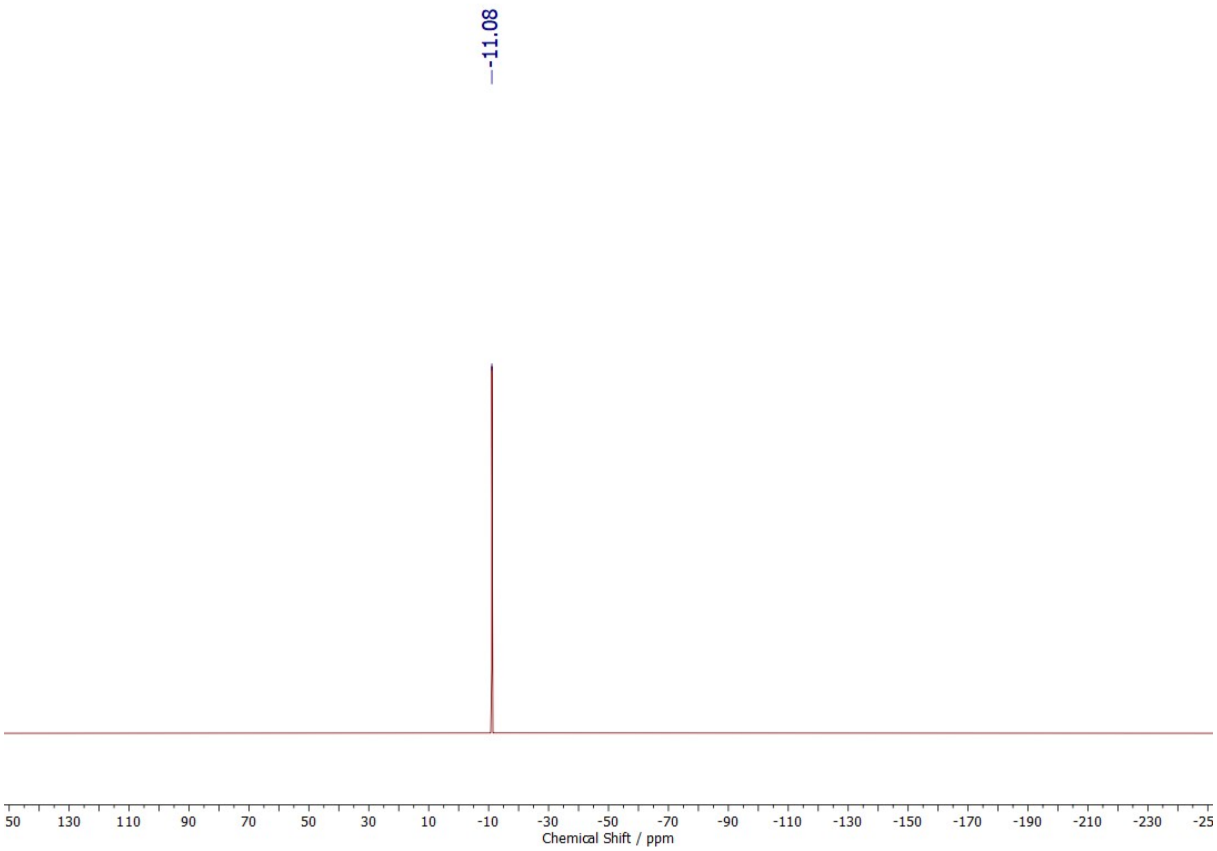

Figure S3. $^{31}P\{^{1}H\}$ NMR spectrum of diphenylphosphinomethanol, C_6D_6, 25 °C

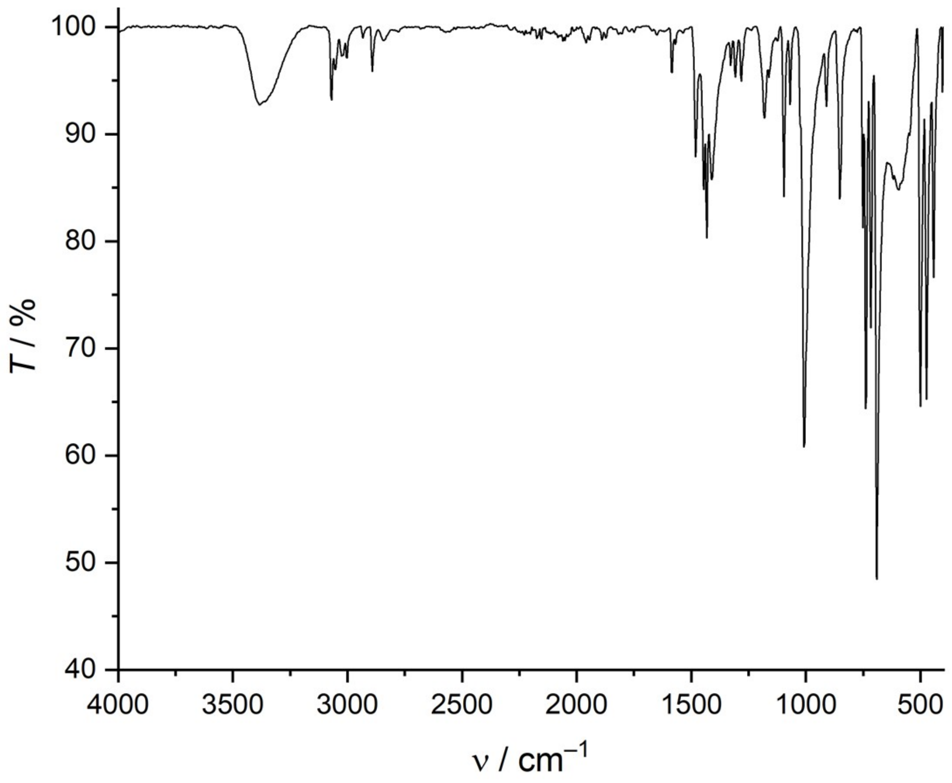

Figure S4. ATR-IR spectrum of diphenylphosphinomethanol, solid, 25 °C.

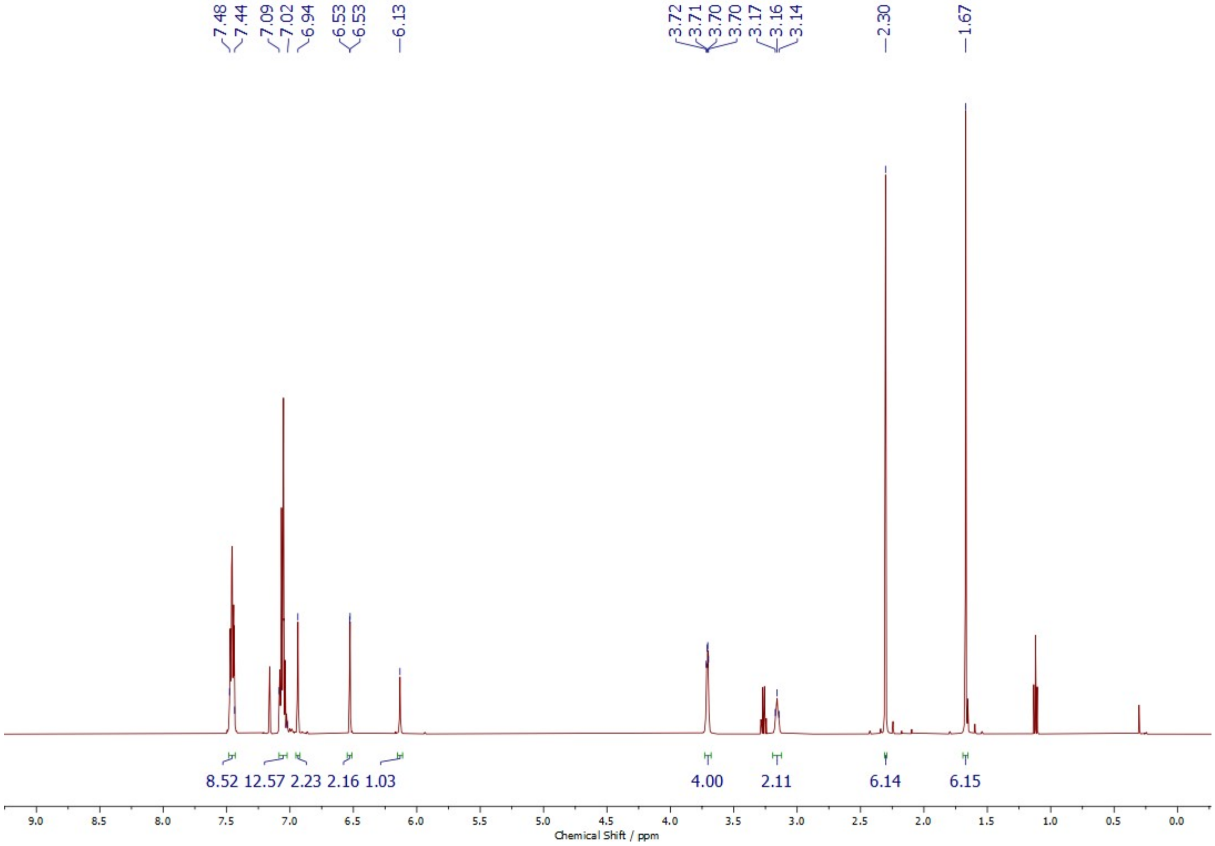

Figure S5. ^{1}H NMR spectrum of **1**, C_6D_6, 25 °C; unlabeled peaks belong to residual Et_2O that can be removed via extensive drying and grease present in the deuterated solvent.

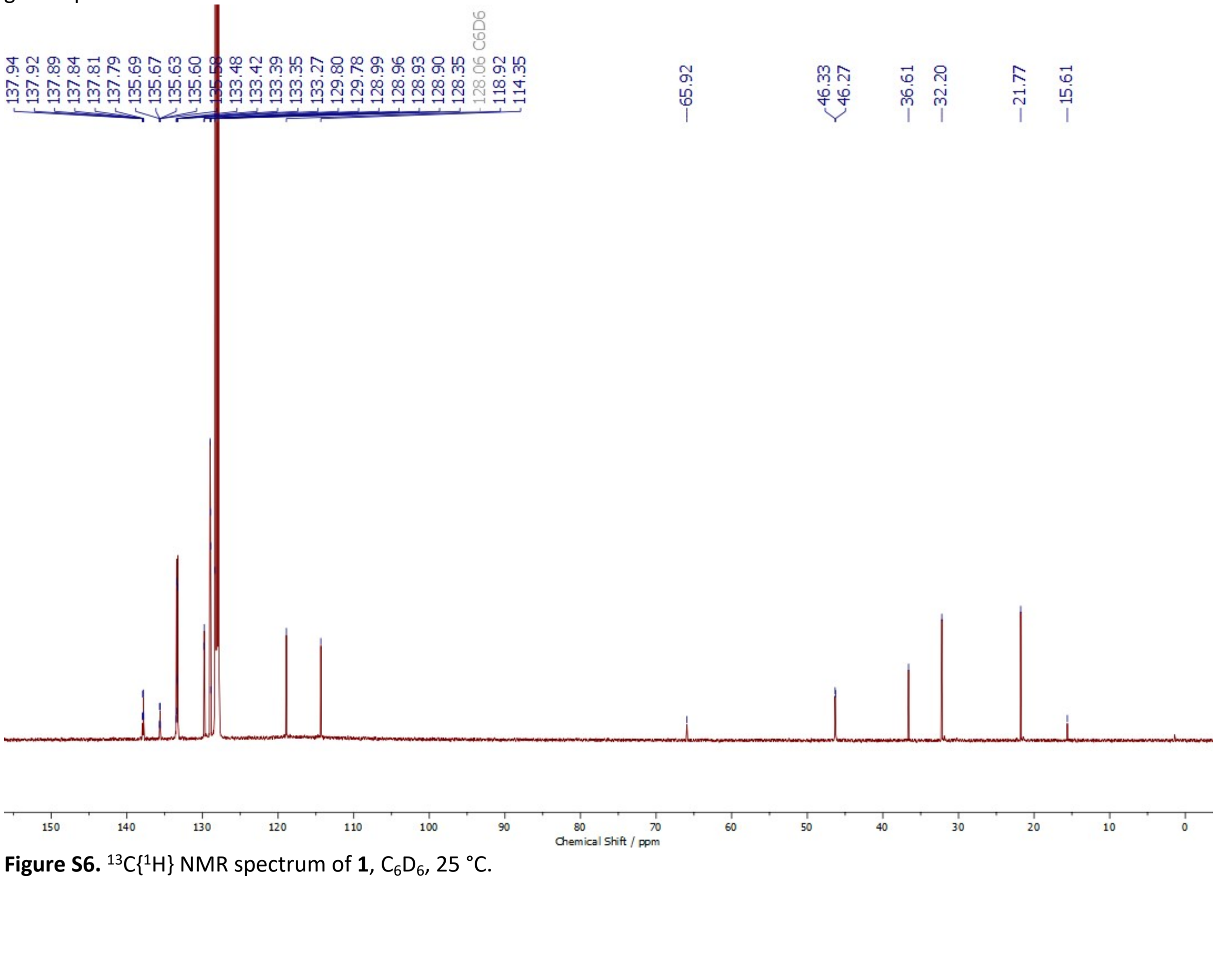

Figure S6. $^{13}C\{^{1}H\}$ NMR spectrum of **1**, C_6D_6, 25 °C.

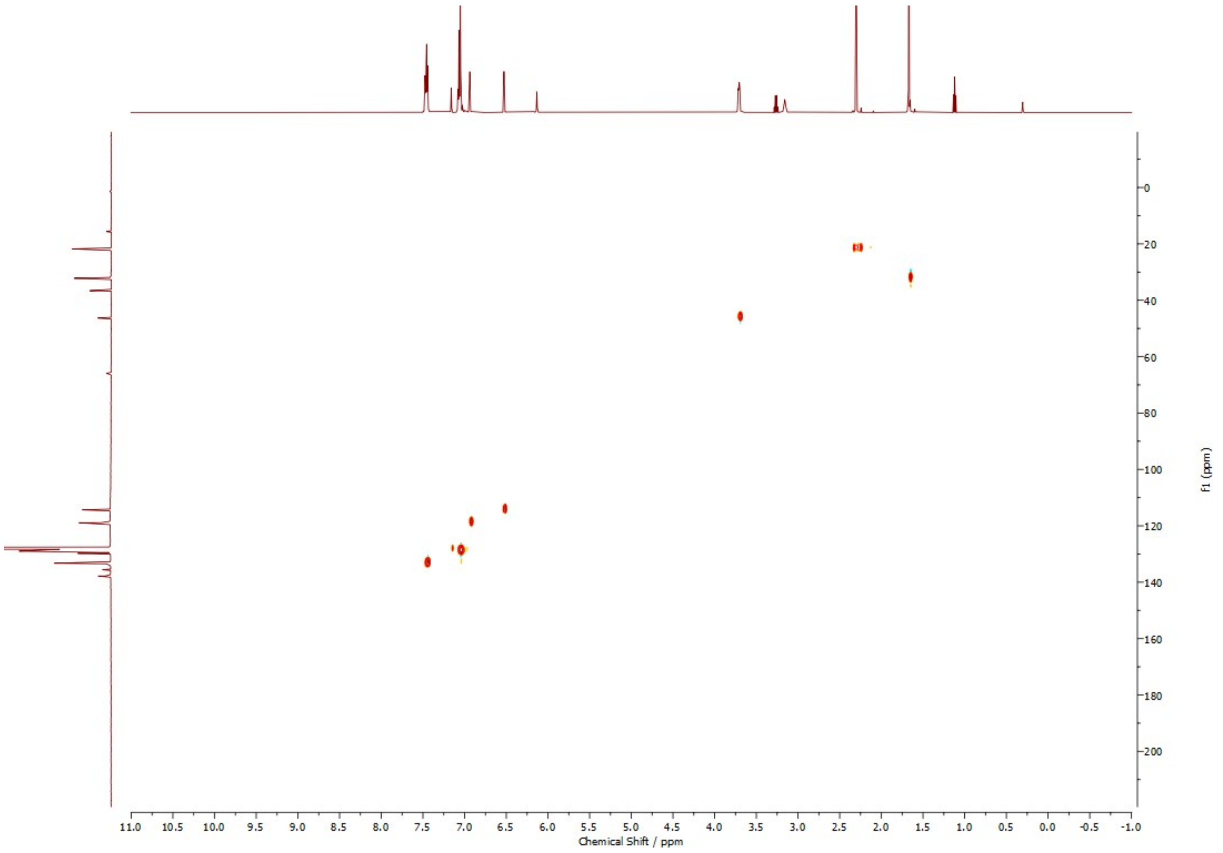

Figure S7. HSQC NMR spectrum of **1**, C_6D_6, 25 °C.

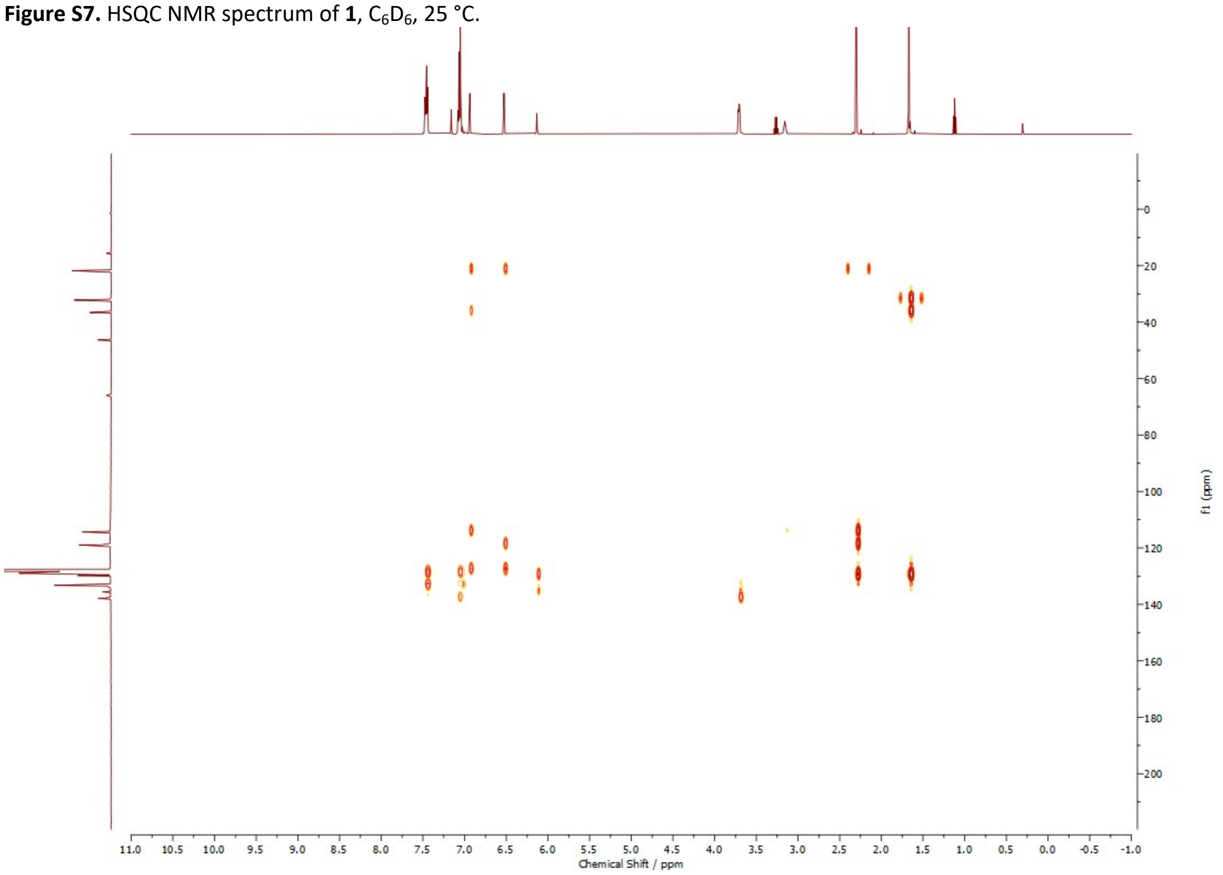

Figure S8. HMBC NMR spectrum of **1**, C_6D_6, 25 °C.

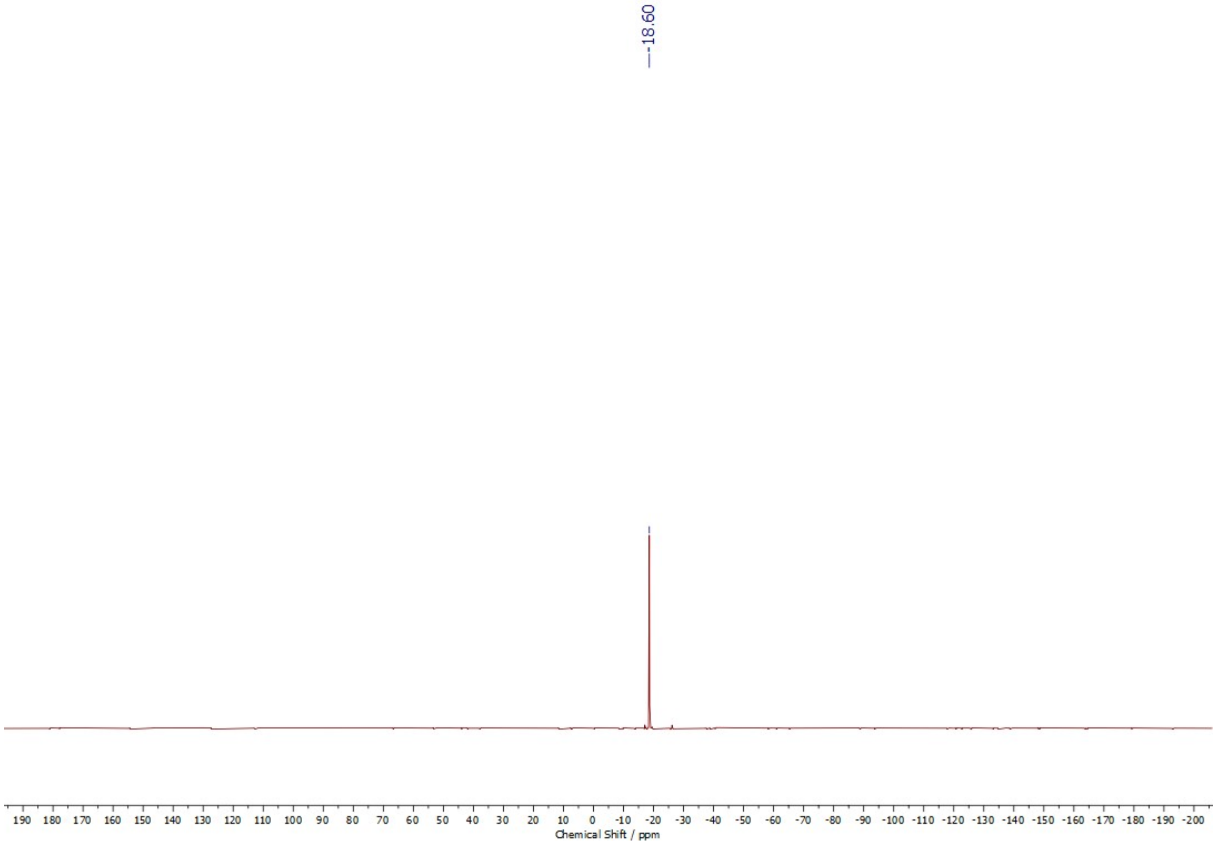

Figure S9. $^{31}P\{^{1}H\}$ NMR spectrum of **1**, C_6D_6, 25 °C.

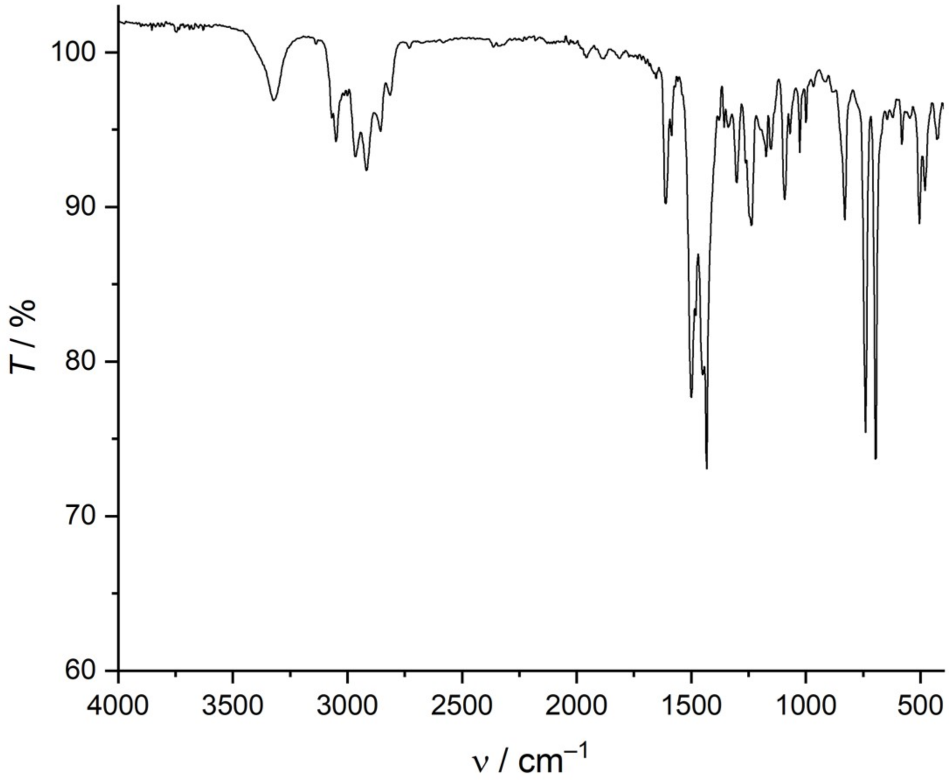

Figure S10. ATR-IR spectrum of **1**, solid, 25 °C.

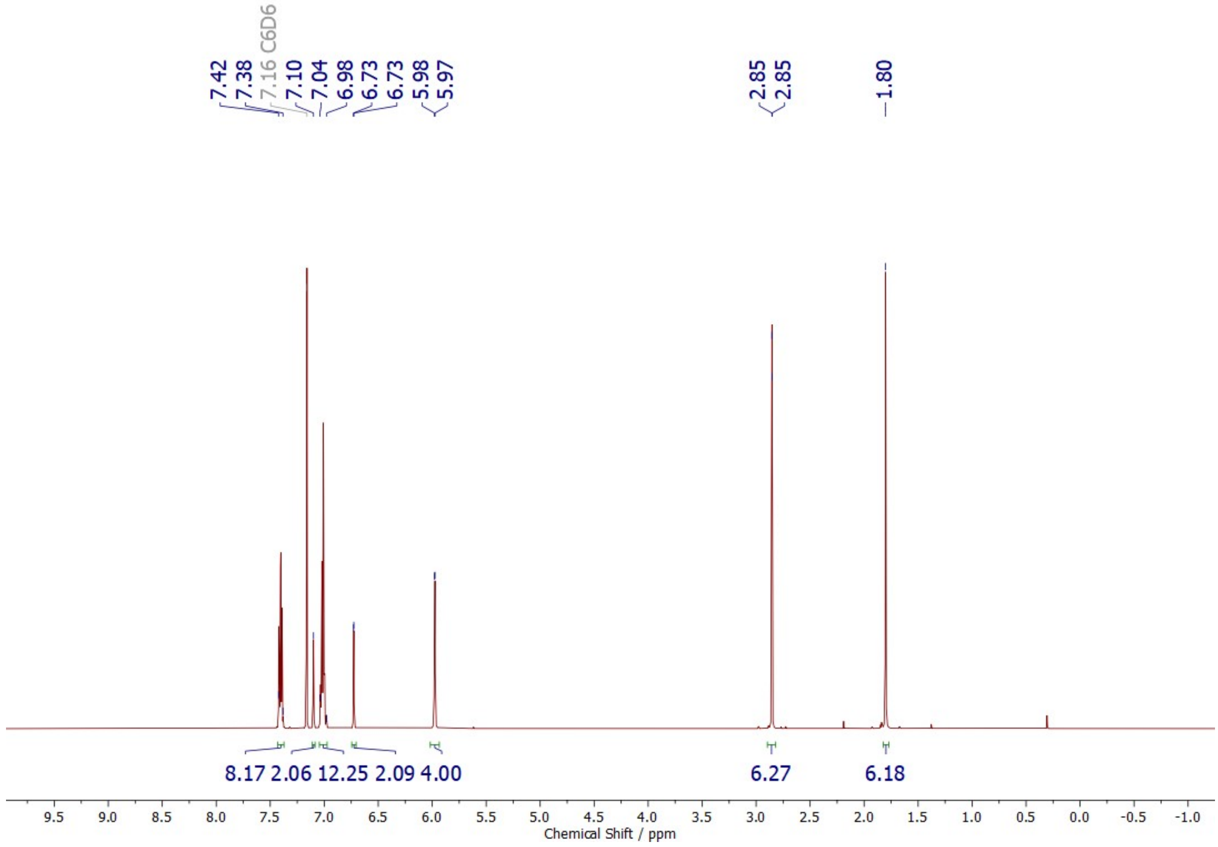

Figure S11. ^{1}H NMR spectrum of **2**, C_6D_6, 25 °C.

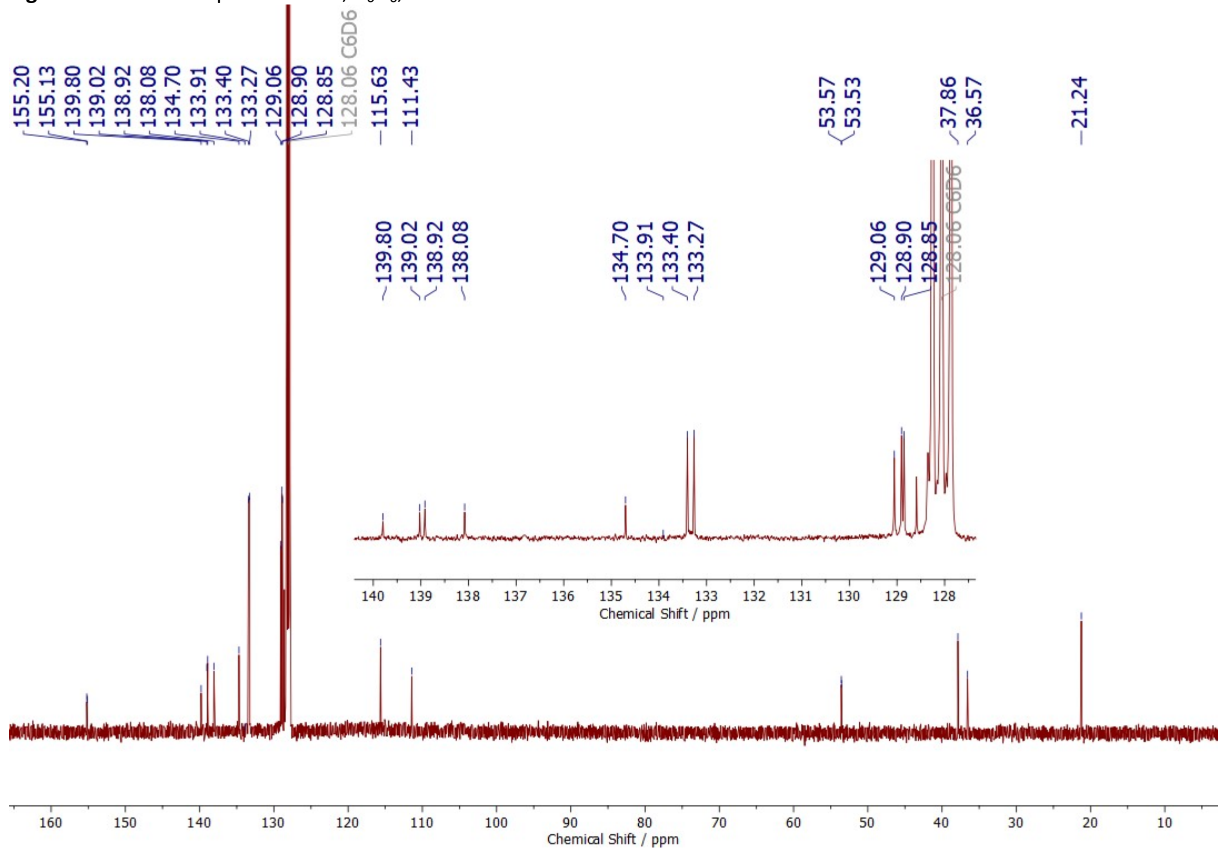

Figure S12. $^{13}C\{^{1}H\}$ NMR spectrum of **2**, C_6D_6, 25 °C.

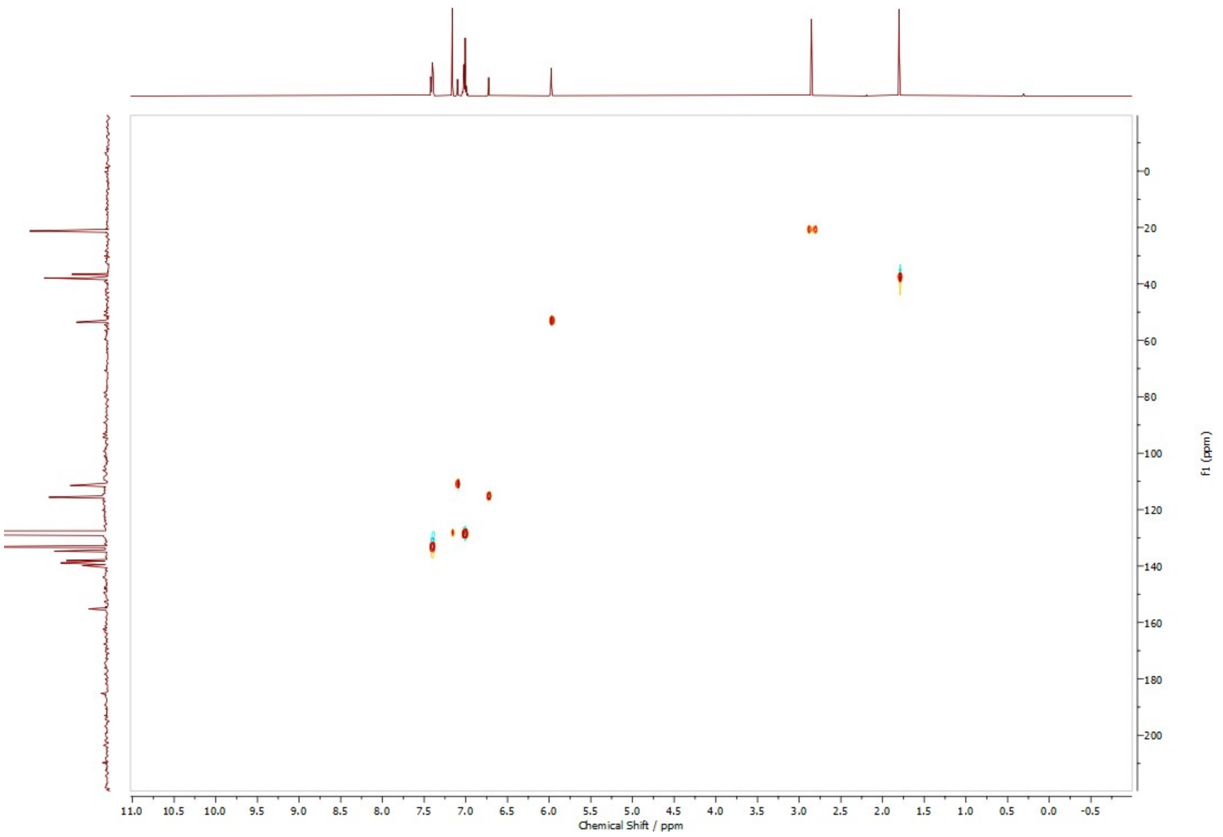

Figure S13. HSQC NMR spectrum of **2**, C_6D_6, 25 °C.

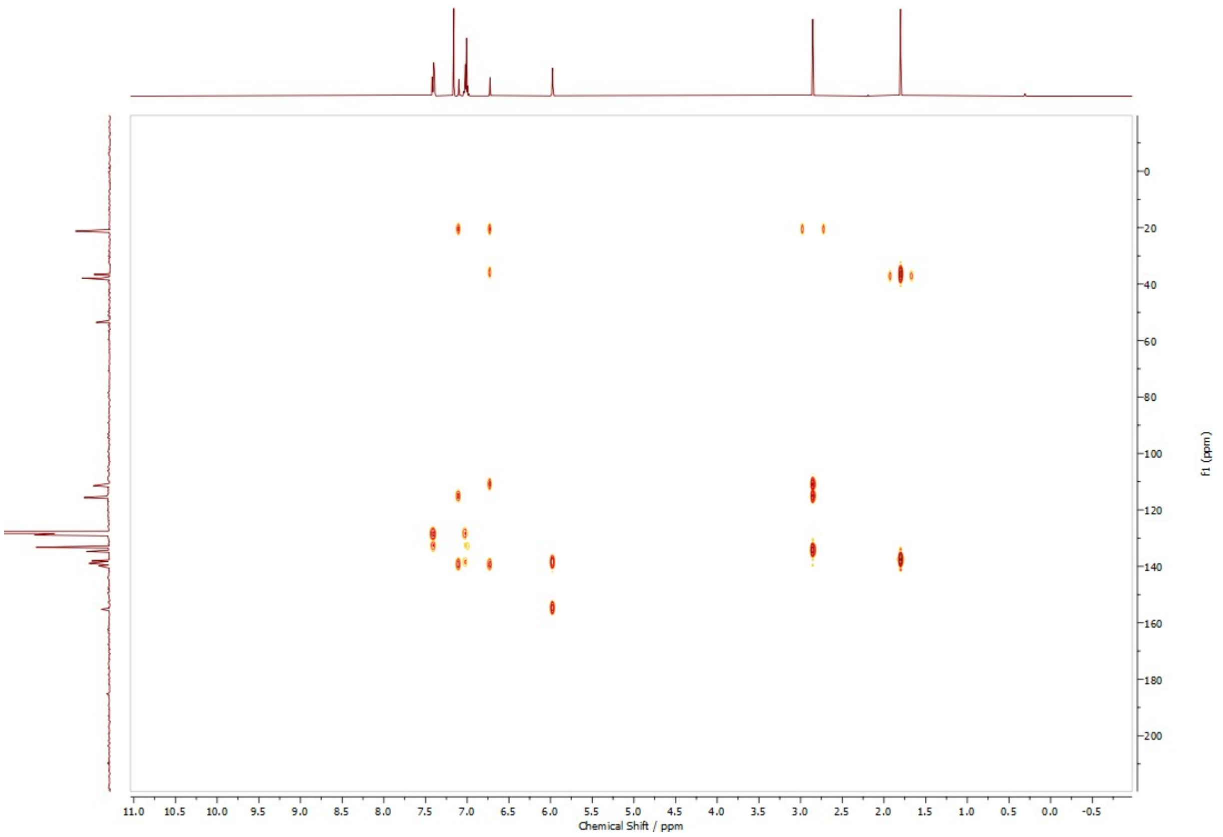

Figure S14. HMBC NMR spectrum of **2**, C_6D_6, 25 °C.

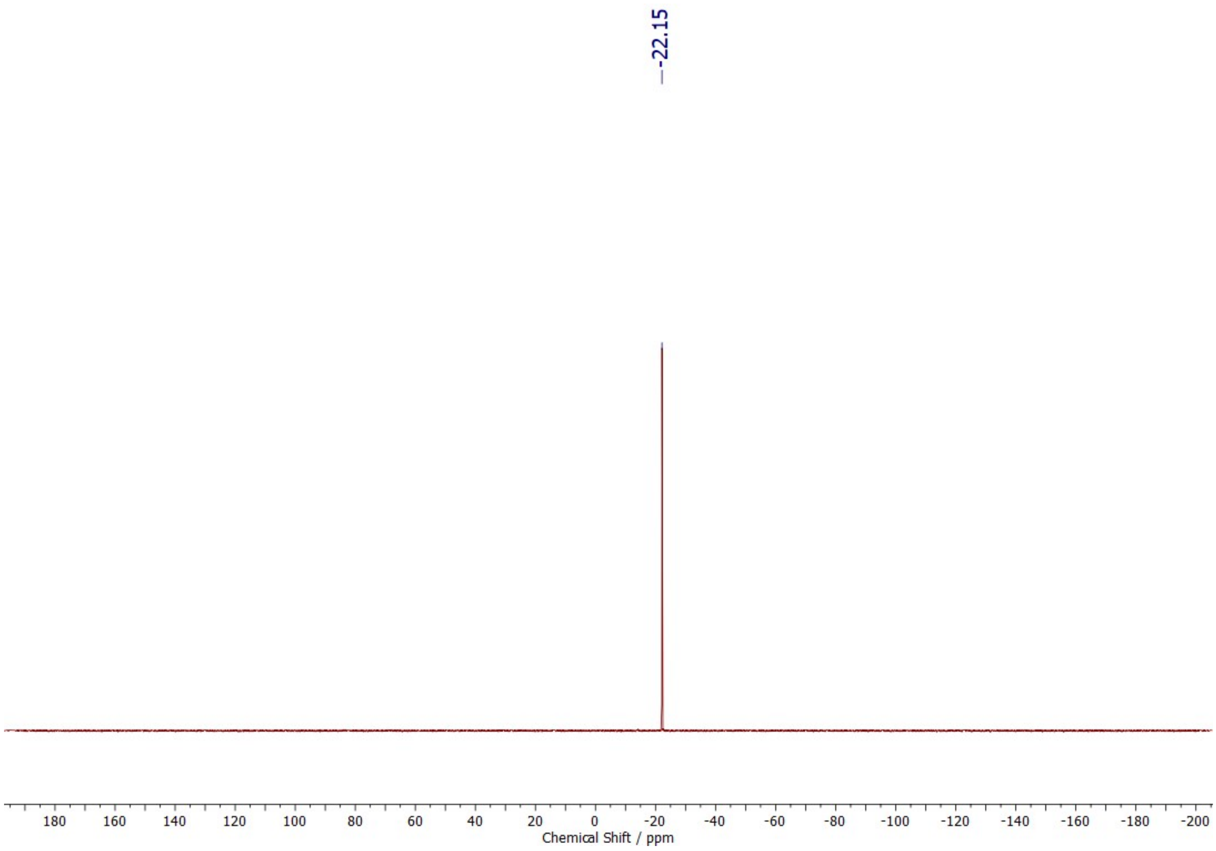

Figure S15. $^{31}P\{^{1}H\}$ NMR spectrum of **2**, C_6D_6, 25 °C

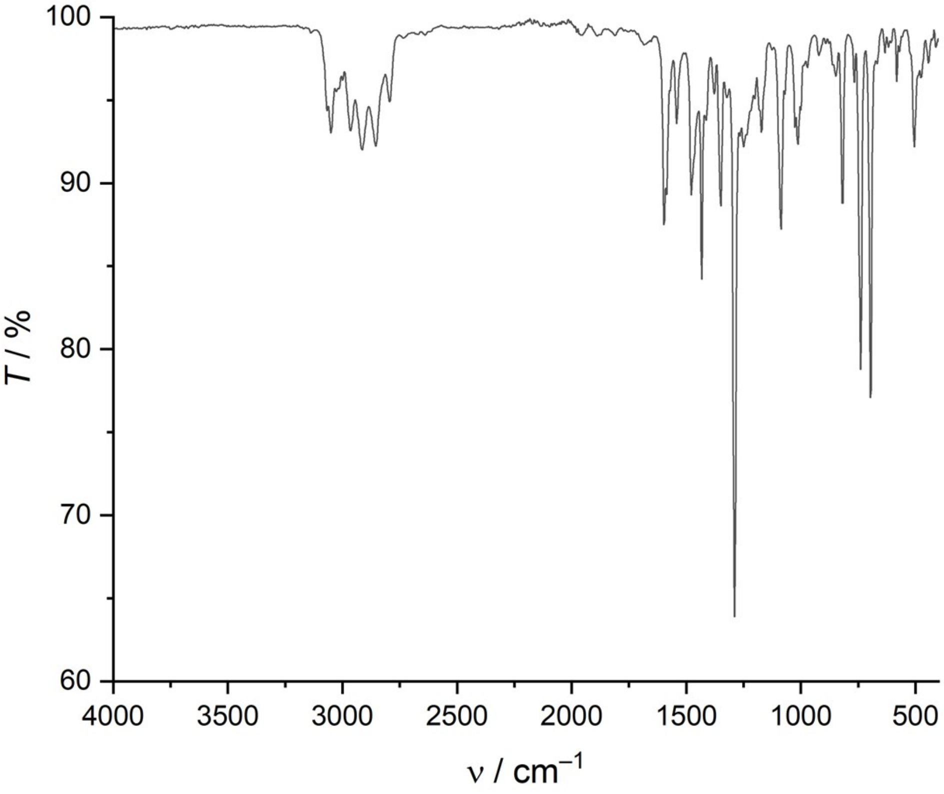

Figure S16. ATR-IR spectrum of **2**, solid, 25 °C.

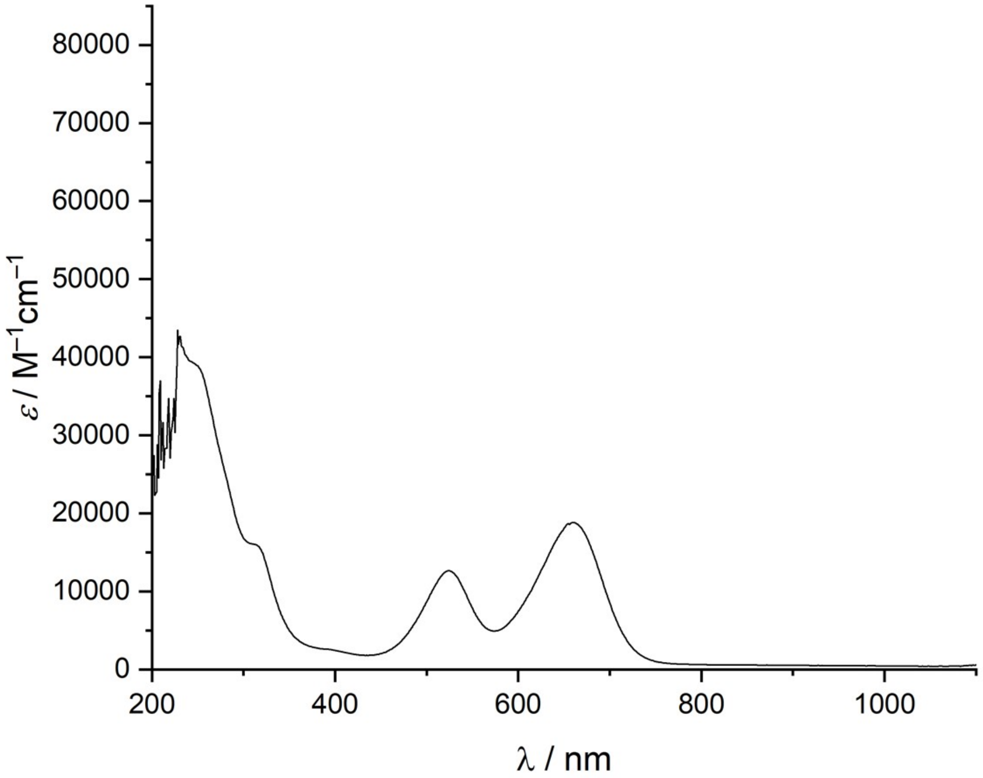

Figure S17. UV/vis spectrum of **2**, DCM, 25 °C.

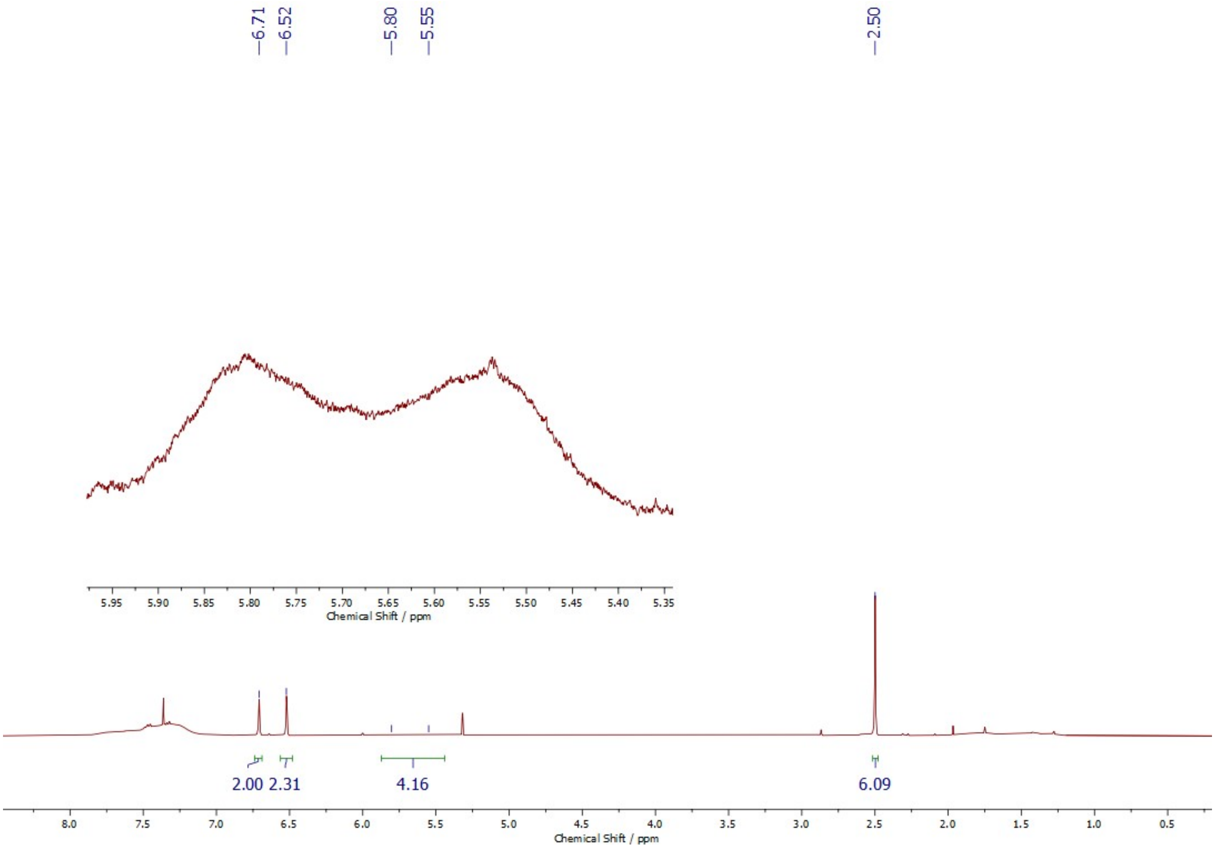

Figure S18. ^{1}H NMR spectrum of **3**, DCM, 25 °C; Inset: Zoomed in ^{1}H NMR spectrum showing the Inequivalence of the severely broadened ligand's methylene protons.

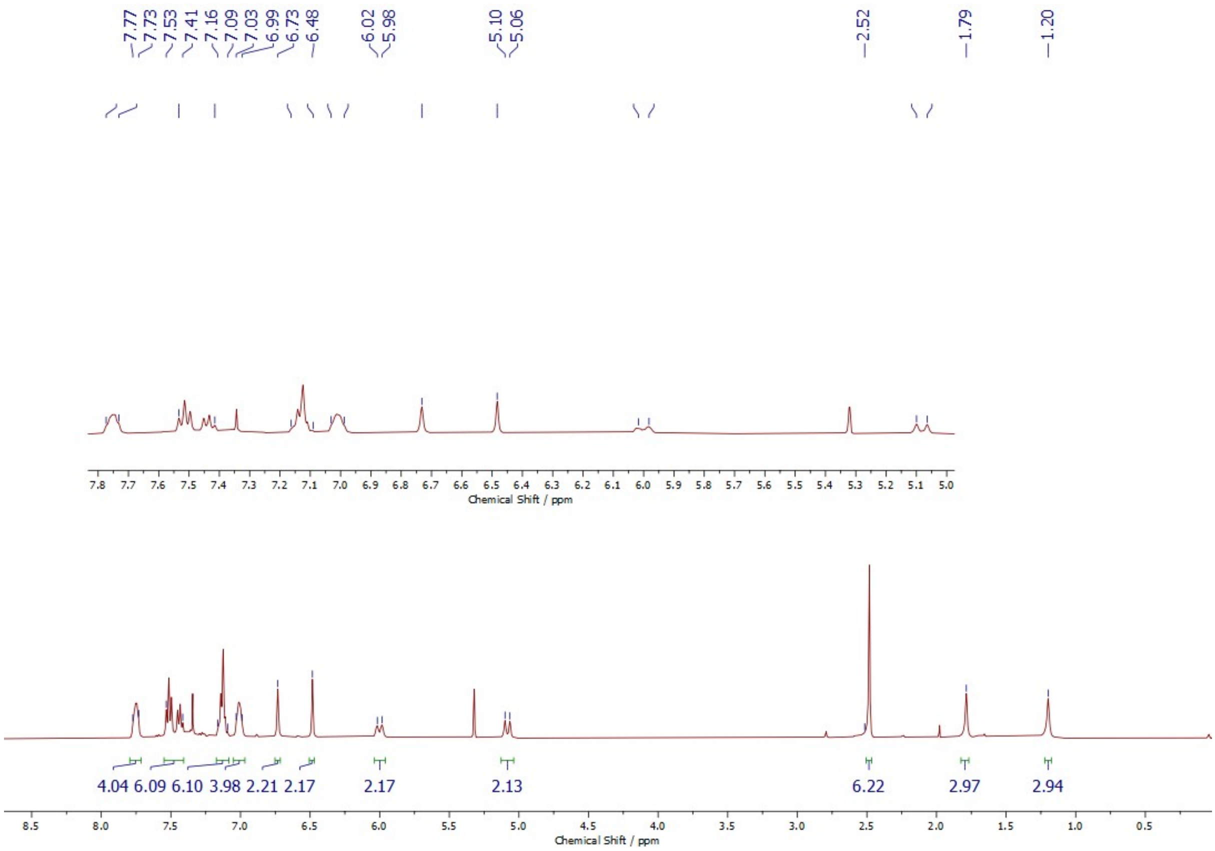

Figure S19. ^{1}H NMR spectrum of **3**, DCM, –60 °C; Inset: Aromatic region of the ^{1}H NMR spectrum revealing sharpening of the ligand signals.

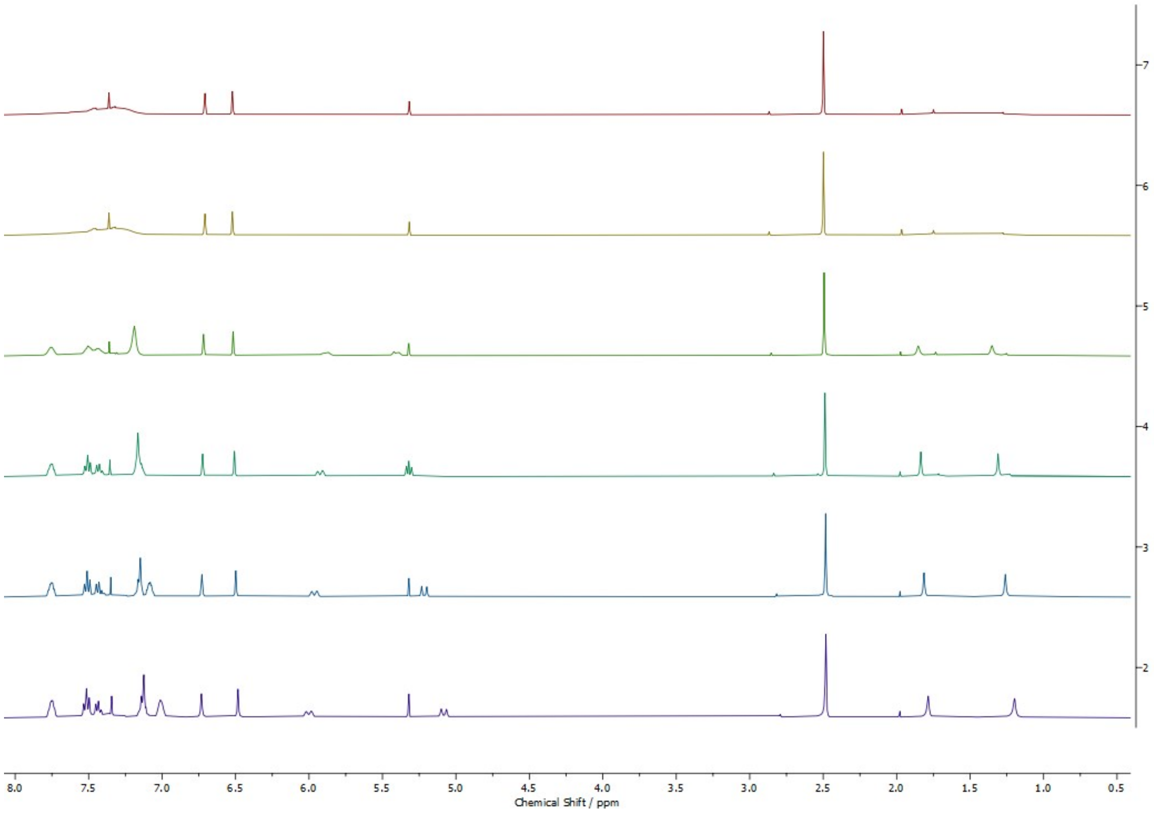

Figure S20. ^{1}H VT NMR of **3**, DCM, 25 °C (red), 0 °C (yellow), 0 °C (ligh green), –20 °C (dark green), –40 °C (blue), –60 °C (purple).

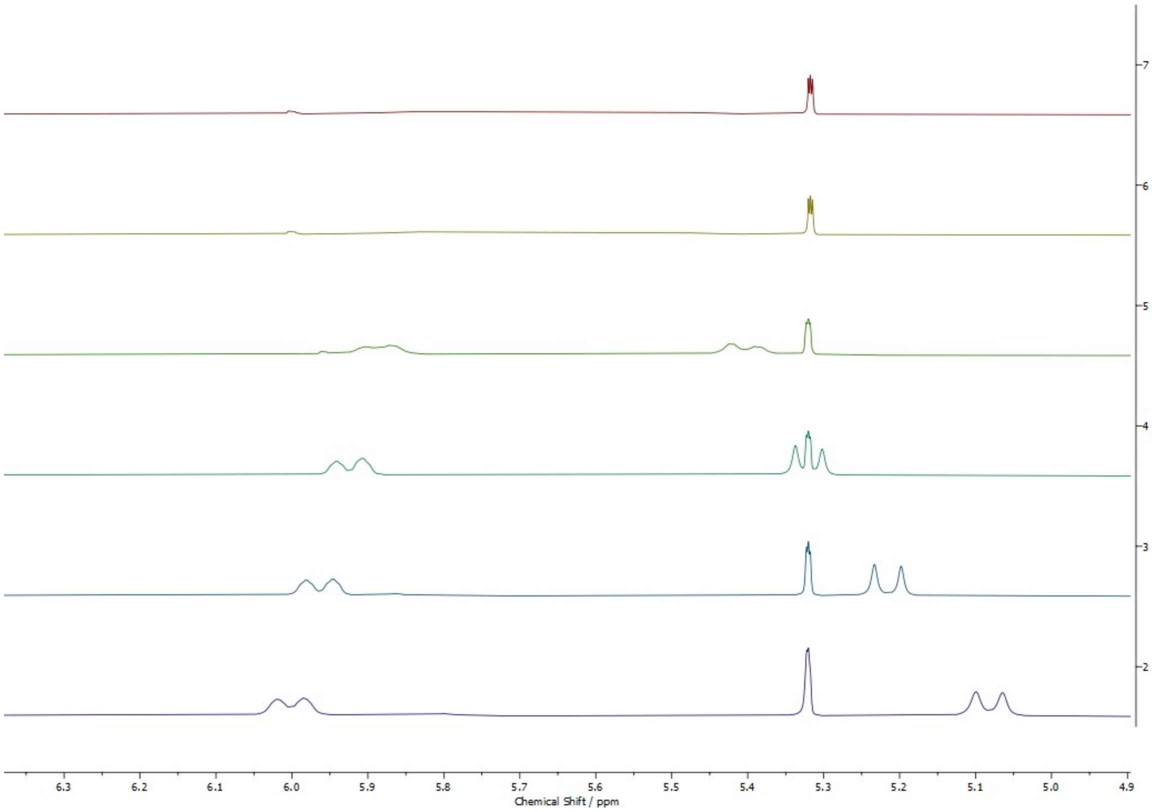

Figure S21. Zoomed in ^{1}H VT NMR of **3**, DCM, 25 °C (red), 0 °C (yellow), 0 °C (light green), –20 °C (dark green), –40 °C (blue), –60 °C (purple).

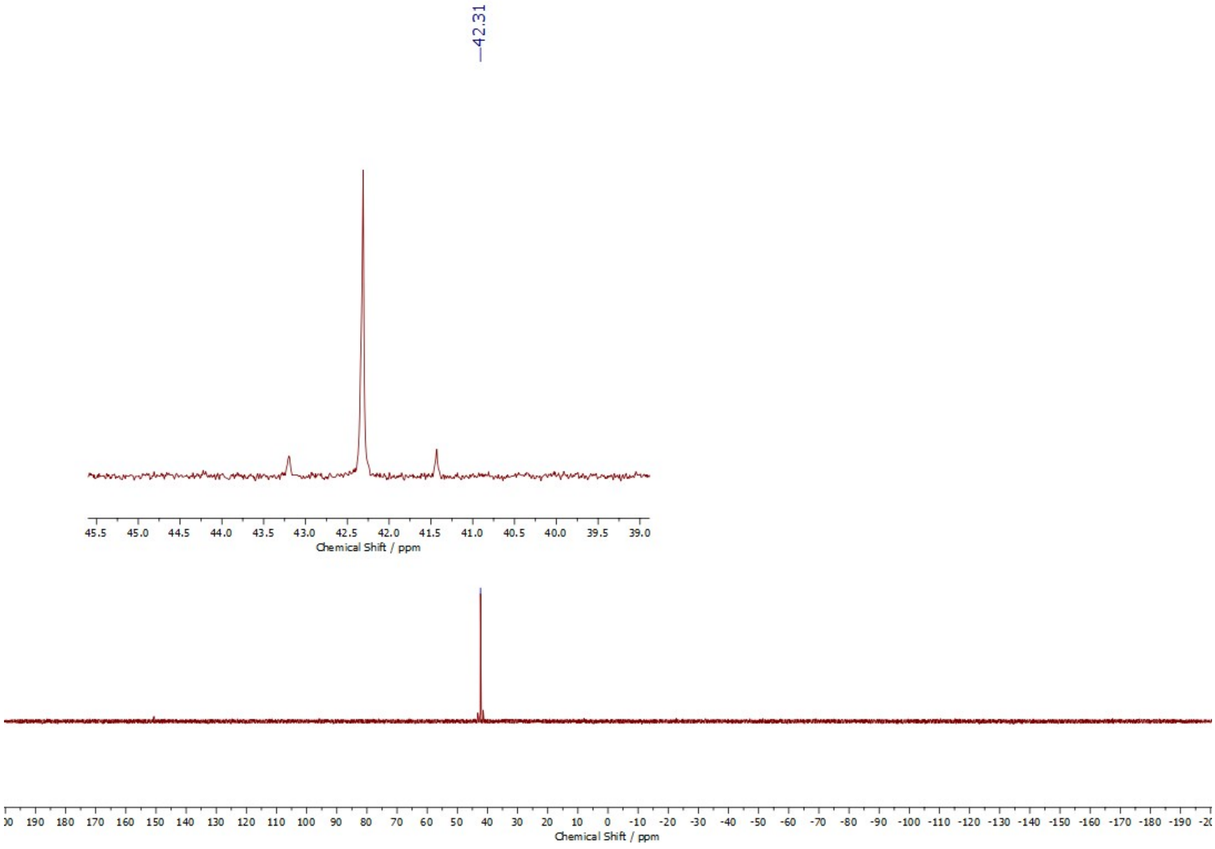

Figure S22. $^{31}P\{^{1}H\}$ NMR of **3**, DCM, 25 °C; Inset: zoomed in $^{31}P\{^{1}H\}$ NMR spectrum displaying the P–W coupling.

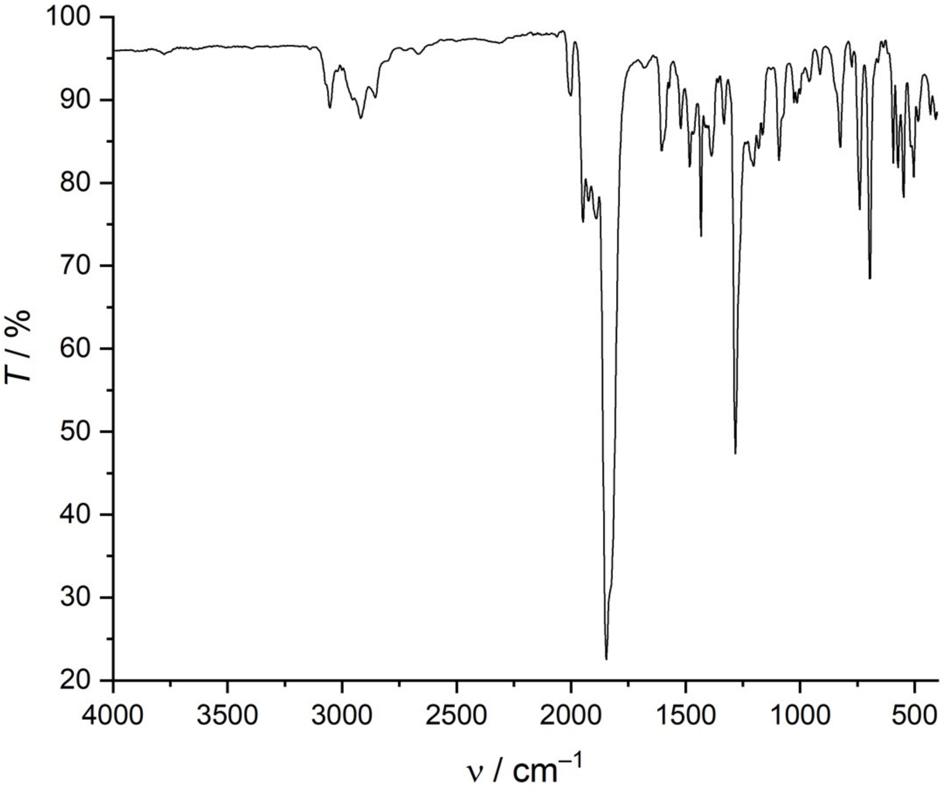

Figure S23. ATR-IR spectrum of **3**, solid, 25 °C.

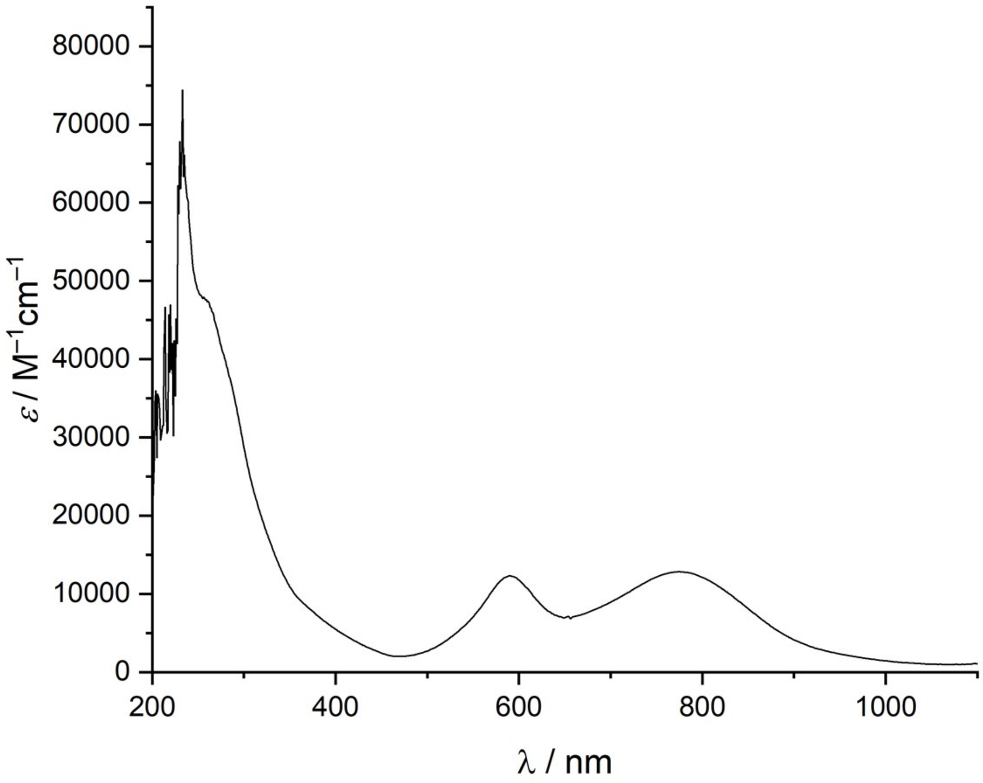

Figure S24. UV/vis spectrum of **3**, DCM, 25 °C.

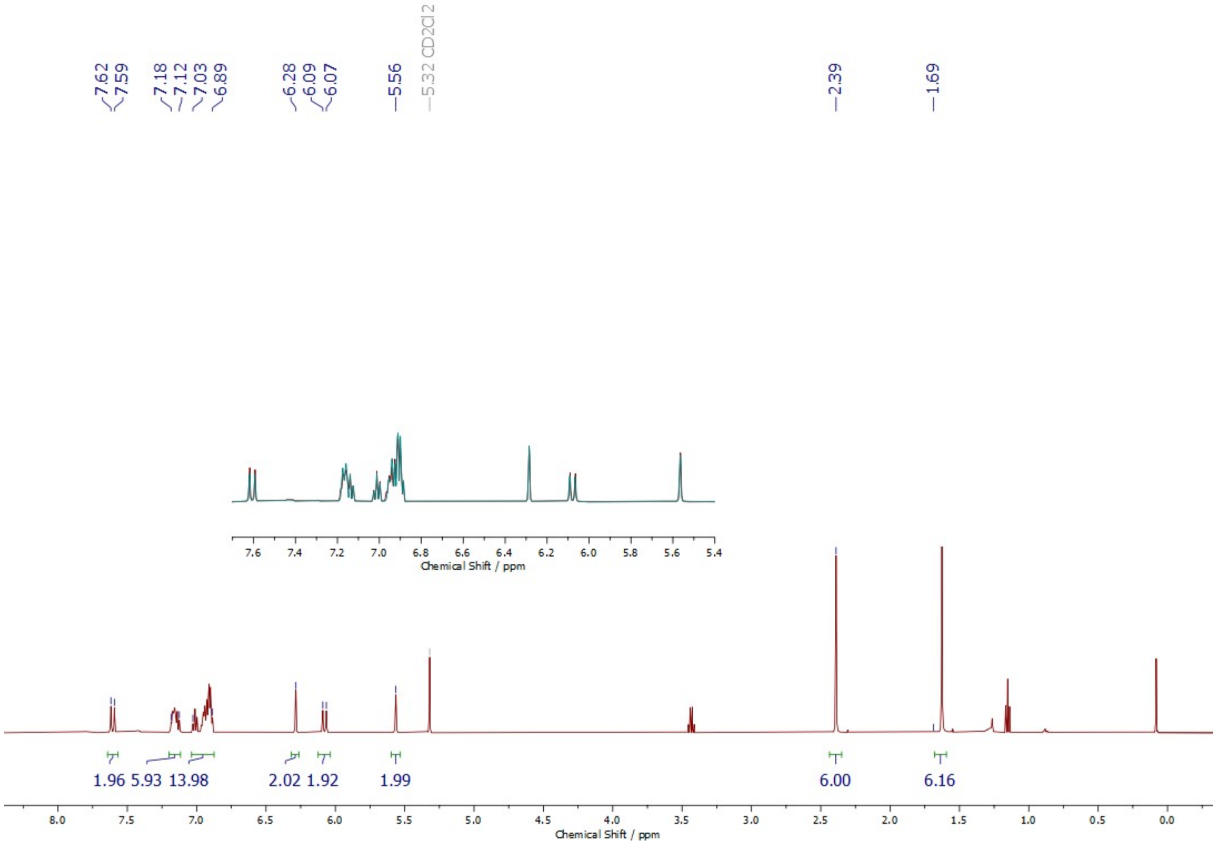

Figure S25. ^{1}H NMR spectrum of **4**, DCM, 25 °C, unlabeled peaks belong to residual Et_2O/hexane that can be removed via extensive drying and grease present in the deuterated solvent.; Inset: ^{1}H and $^{1}H\{^{31}P\}$ NMR spectrum of the aromatic region.

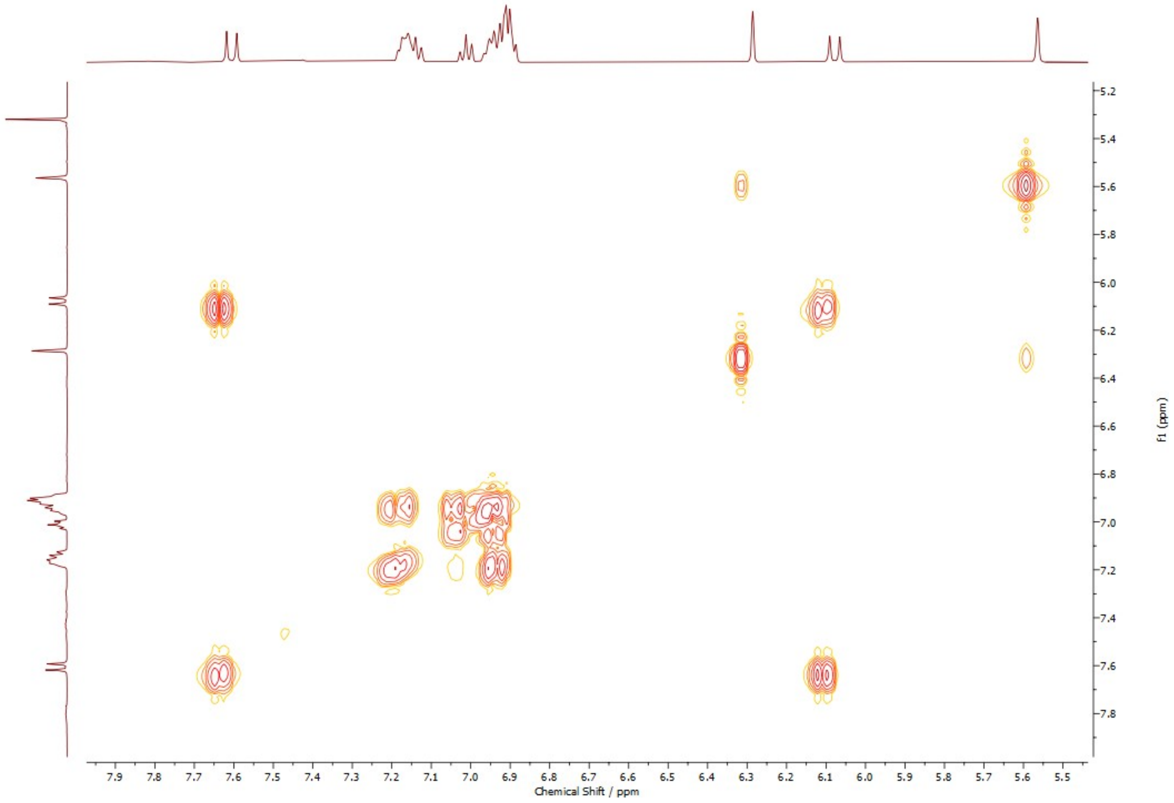

Figure S26. COSY NMR spectrum of **4**, DCM, 25 °C.

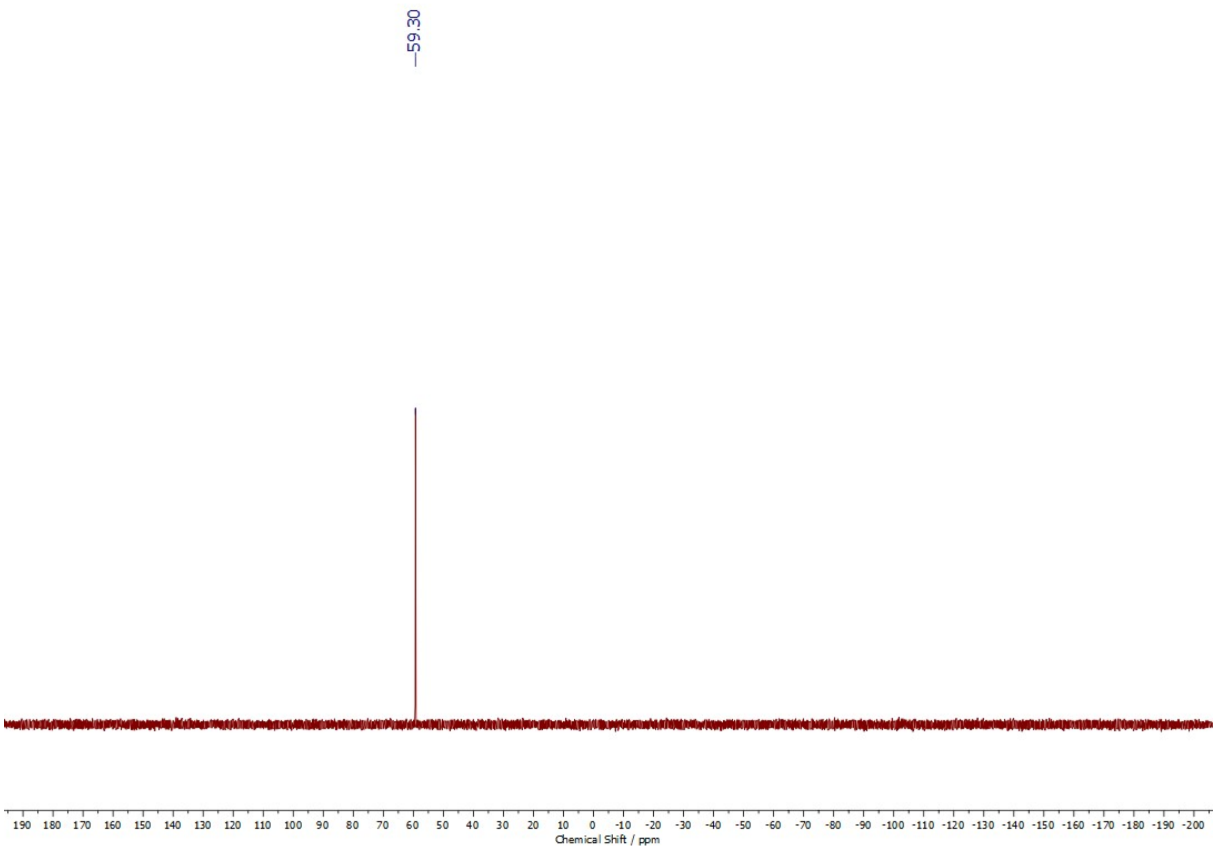

Figure S27. $^{31}P\{^{1}H\}$ NMR spectrum of **4**, DCM, 25 °C.

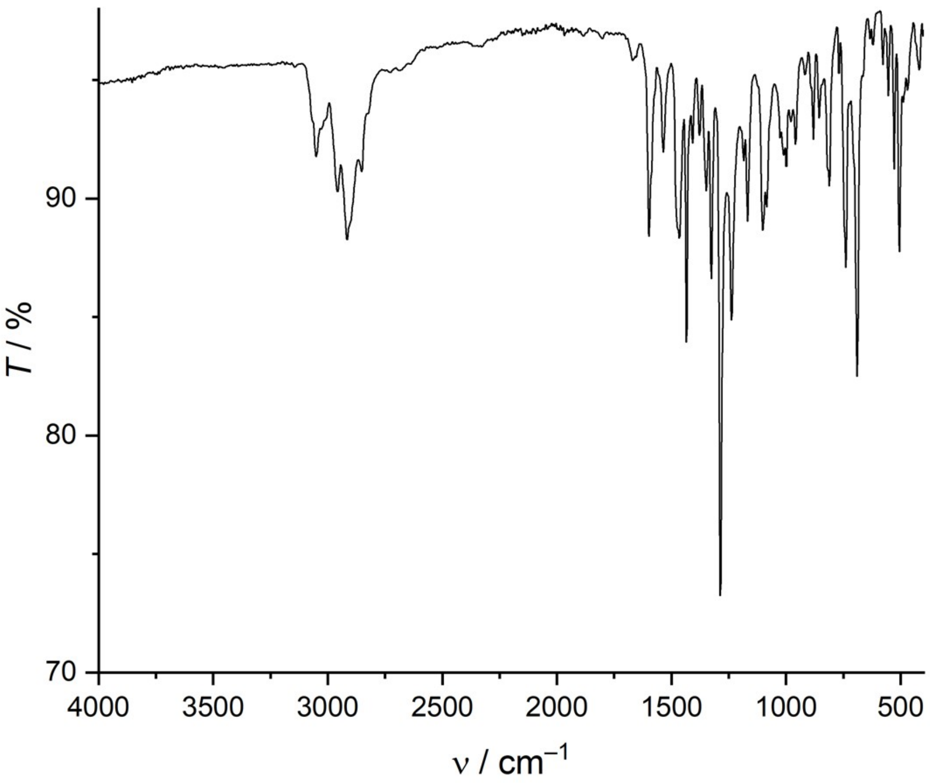

Figure S28. ATR-IR spectrum of **4**, solid, 25 °C.

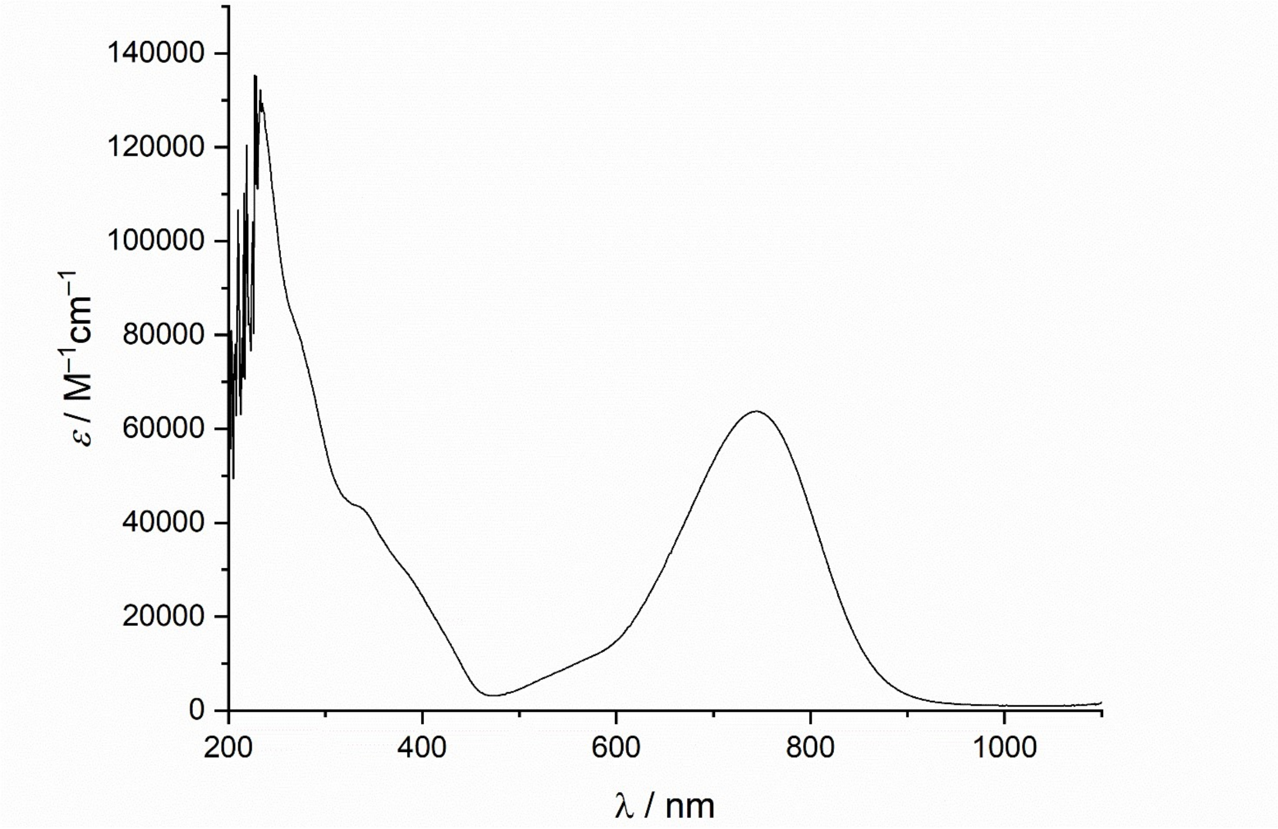

Figure S29. UV/vis spectrum of **4**, DCM, 25 °C.

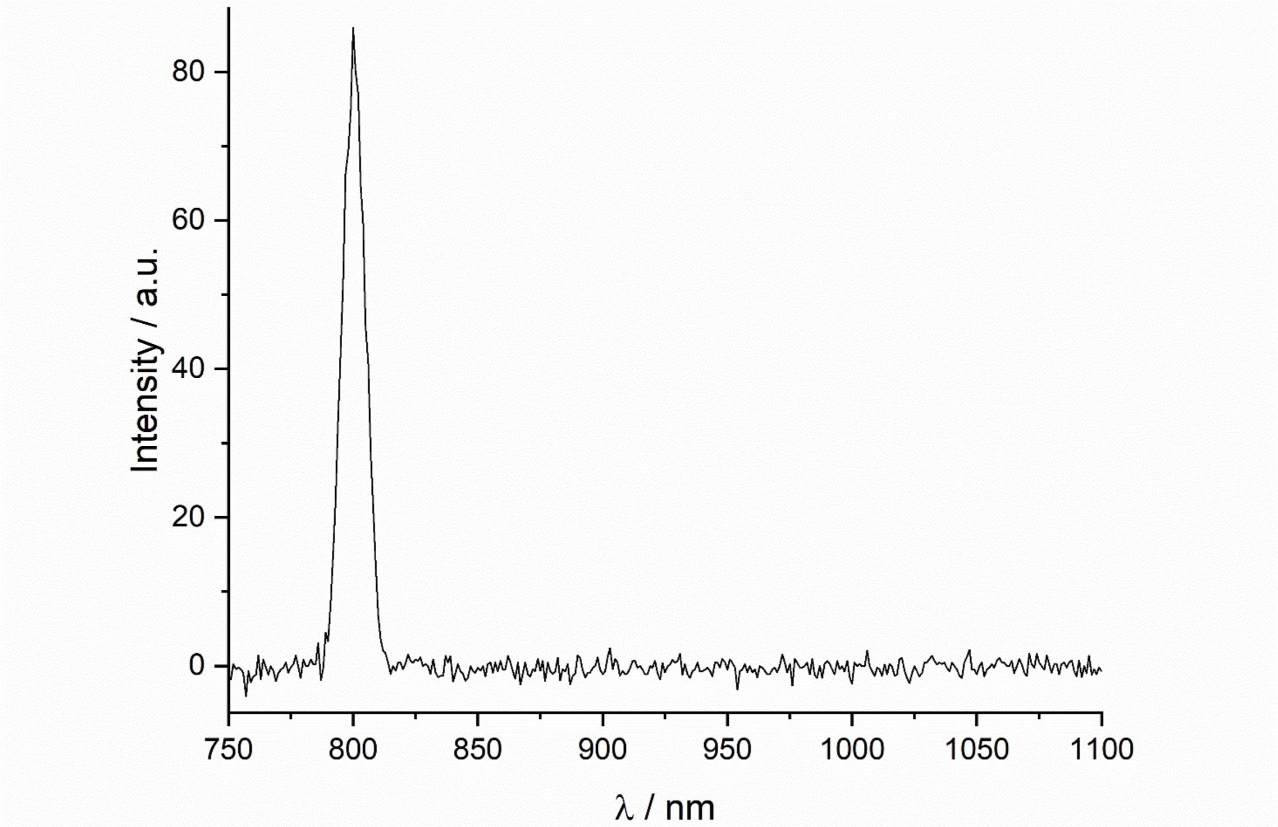

Figure S30. Emission spectrum of **4**, 10^{-5} M, DCM, 25 °C, λ_{ex} = 800 nm; no detectable fluorescence and only reflection was detected.

DOSY NMR Studies

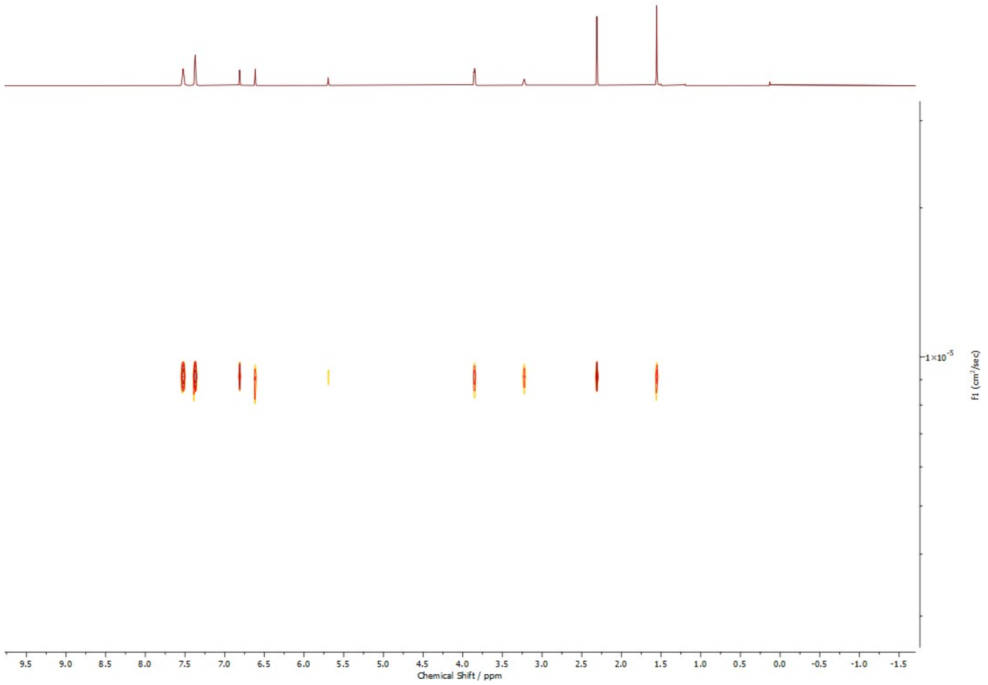

Figure S31. DOSY NMR of **1**, DCM, 25 °C.

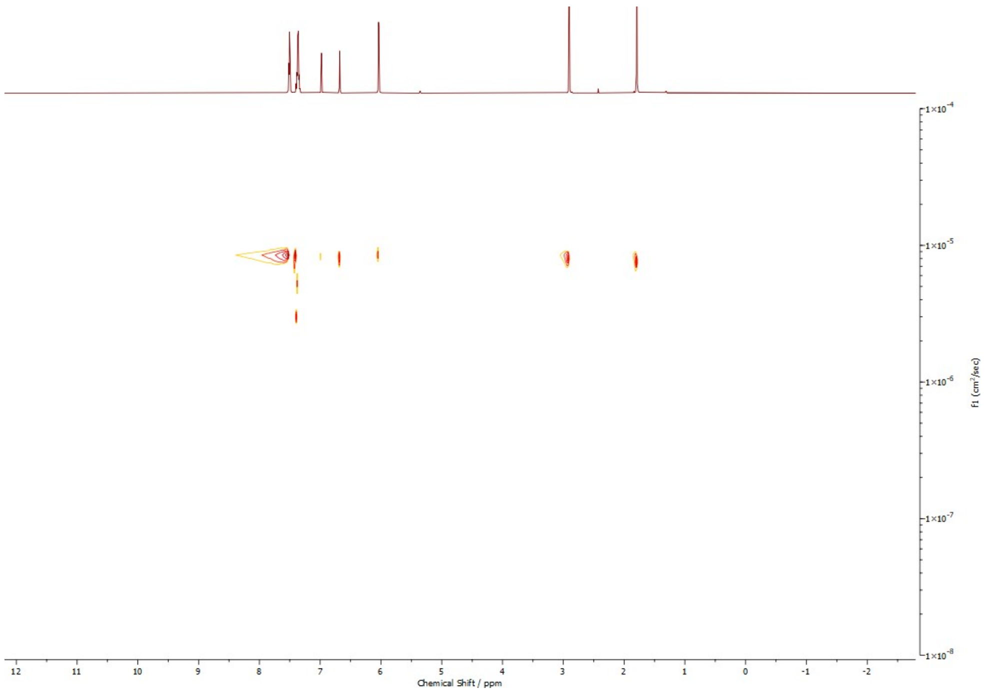

Figure S32. DOSY NMR of **2**, DCM, 25 °C.

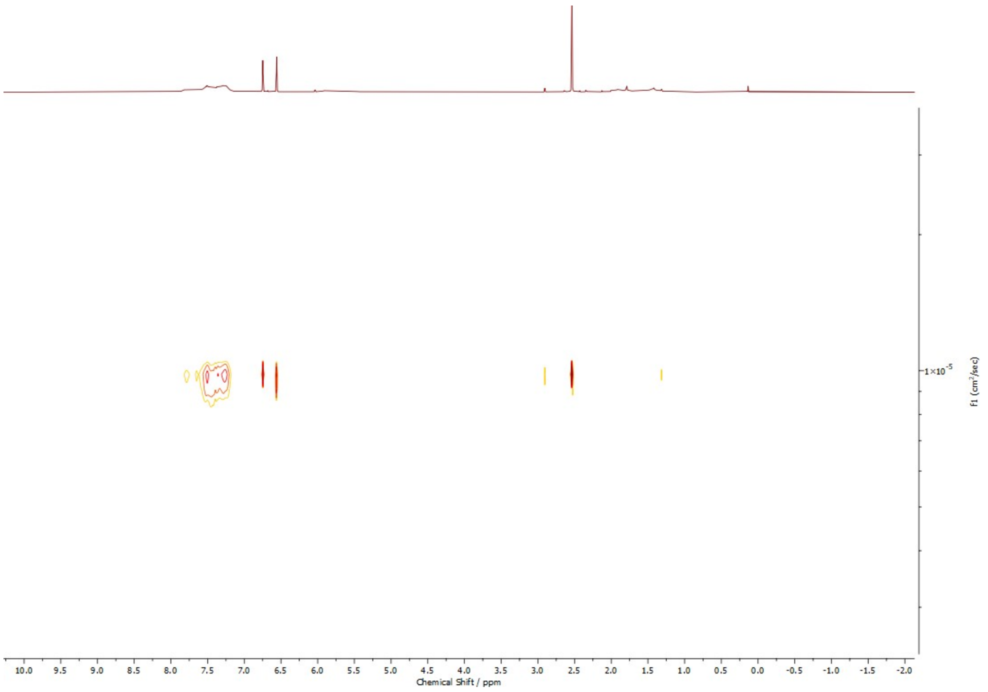

Figure S33. DOSY NMR of **3**, DCM, 25 °C.

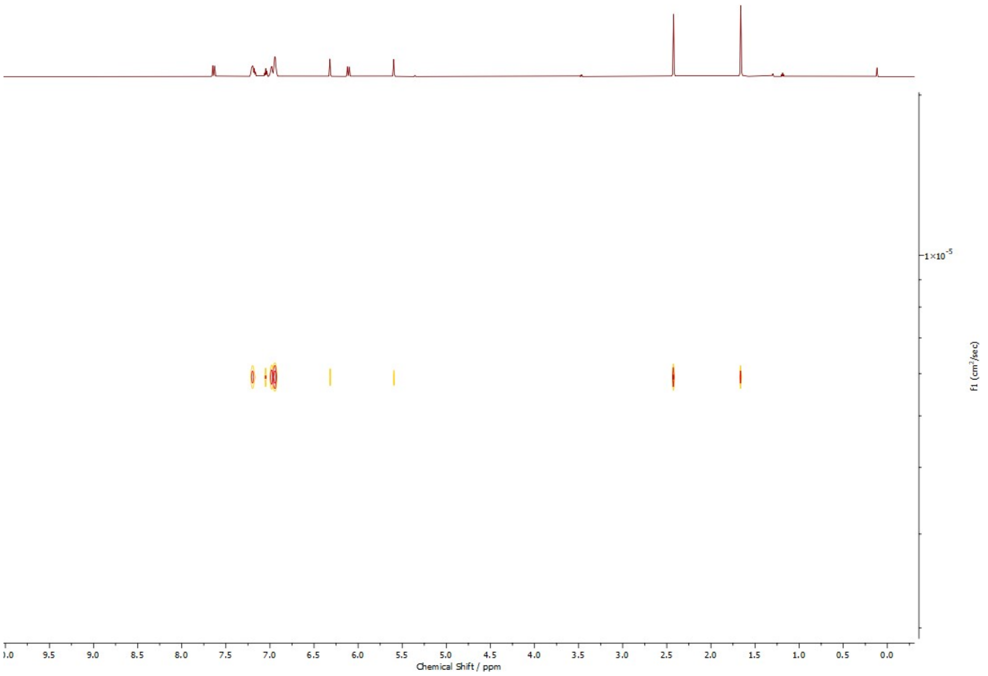

Figure S34. DOSY NMR of **4**, DCM, 25 °C.

Table S1. Diffusion coefficients derived from DOSY NMR.

Compound	Diffusion coefficient (10^{-6} cm²/s)
**1**	9.0
**2**	8.1
**3**	10
**4**	5.8

Application of the Stokes-Einstein equation shows that **4**'s radius is 1.7 times larger than **3**'s in solution ($D_1/D_2 = r_2/r_1$). In combination with the observed C_{2v} symmetry on the NMR timescale the connectivity of **4** revealed by SCXRD appears to also be present in solution.

Determination of Lewis-acidities via the Gutmann-Beckett method

The Lewis acidity of **2** and **3** was assessed via addition of one equivalent of Et_3PO and Me_3PS to a dichloromethane solution of the respective complexes including a capillary of PPh_3 as an internal standard. The acceptor number was derived via application of the following formulas:[57,59]

$$AN(Et_3PO) = 2.21 \cdot (\delta(^{31}P\ NMR)_{Sample}/ppm - 41.0)$$

$$AN(Me_3PS) = 6.41 \cdot (\delta(^{31}P\ NMR)_{Sample}/ppm - 29.2)$$

For both species an AN(Et_3PO) of 20 was obtained which represents an upper limit since the Lewis acidity of dichloromethane is 20.4. **2** and **3** both feature an AN(Me_3PS) of 8 rendering them weakly Lewis-acidic in line with computed FIAs.

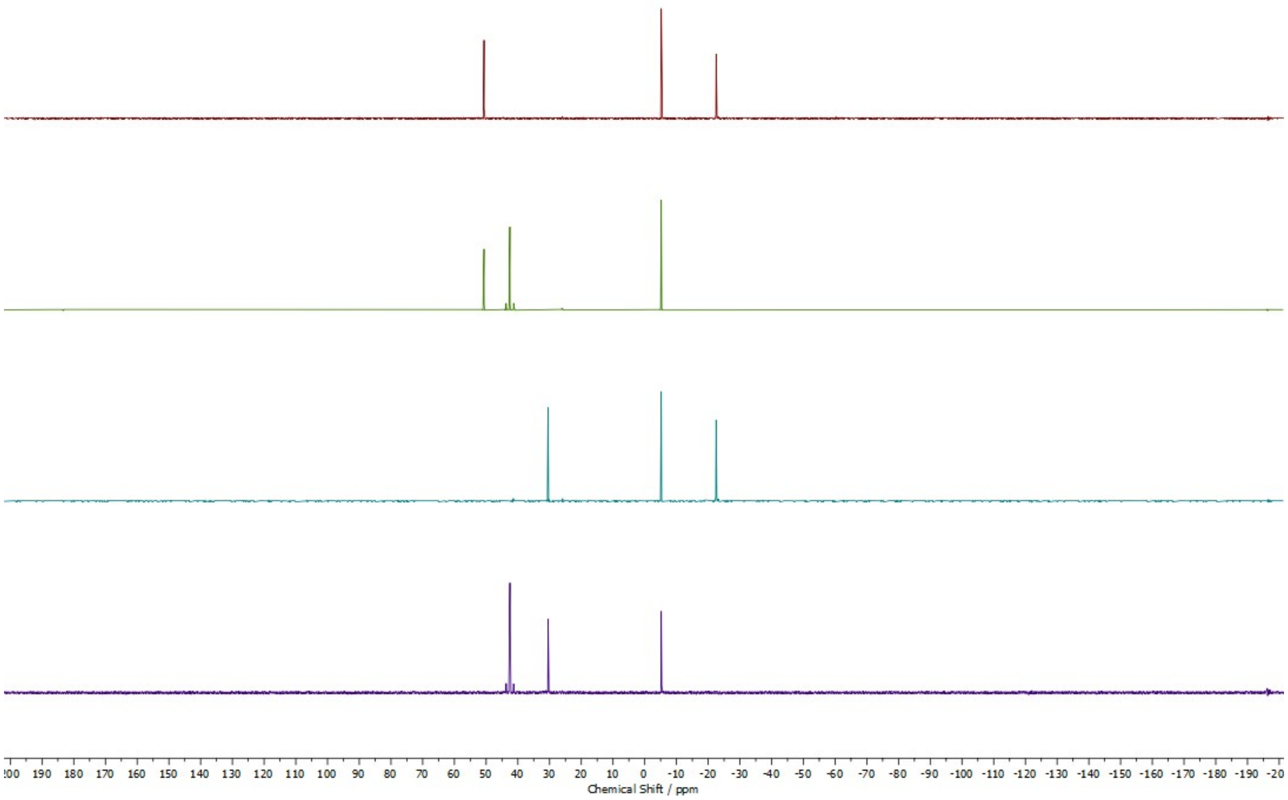

Figure S35. $^{31}P\{^1H\}$ NMR spectra of **2** (red) and **3** (green) in the presence of one equivalent of Et_3PO; $^{31}P\{^1H\}$ NMR spectra of **2** (teal) and **3** (purple) in the presence of one equivalent of Me_3PS, DCM, 25 °C, PPh_3 capillary as internal standard.

Cyclic voltammetry

2 features undefined, irreversible oxidative events on the cyclic voltammogram that are accompanied by rapid fouling of the glassy carbon electrode. To partially prevent this, **2** was measured with ferrocene present in solution providing an internal reference during measurement to prevent overoxidation of **2**. Nevertheless, significant electrode fouling was observed as indicated by the loss of the ferrocene/ferrocenium reverse peak and increasing loss of resolution. After ca. 6 measurements no defined peaks were observable anymore.

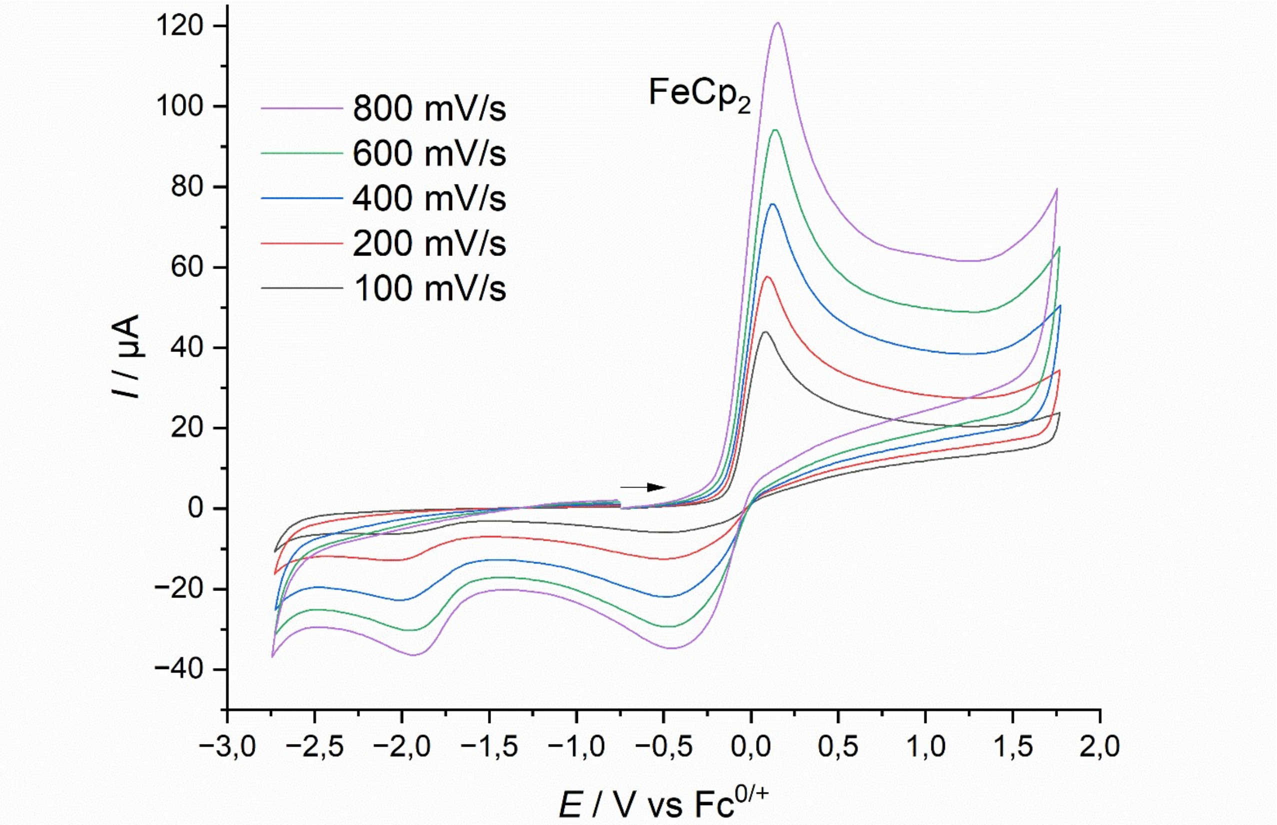

Figure S36. CV of **2** in DCM, 0.1 M $[NBu_4][PF_6]$, 1 mM **2**, 3 mM ferrocene, 25 °C.

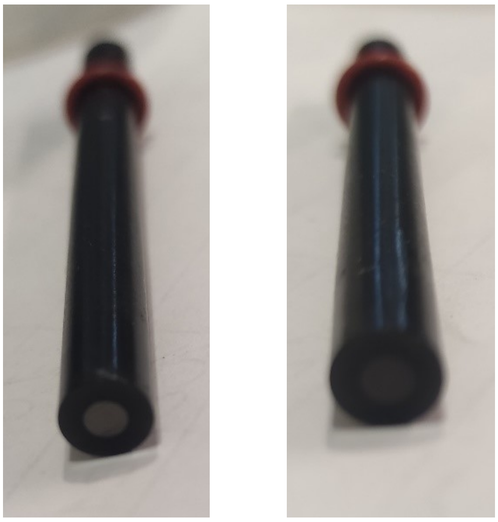

Figure S37. GC working electrode before (left) and after the measurement (right).

Since the measurement in DCM indicates that **2** might feature a reductive wave an additional CV was recorded in THF. Decamethylferrocene was present in solution to prevent fouling of the electrode by accidentally scanning towards high potentials in the absence of an internal reference. A pseudo-reversible reductive event was detected at $E_{1/2}$ = –1.98 V vs $Fc^{0/+}$.

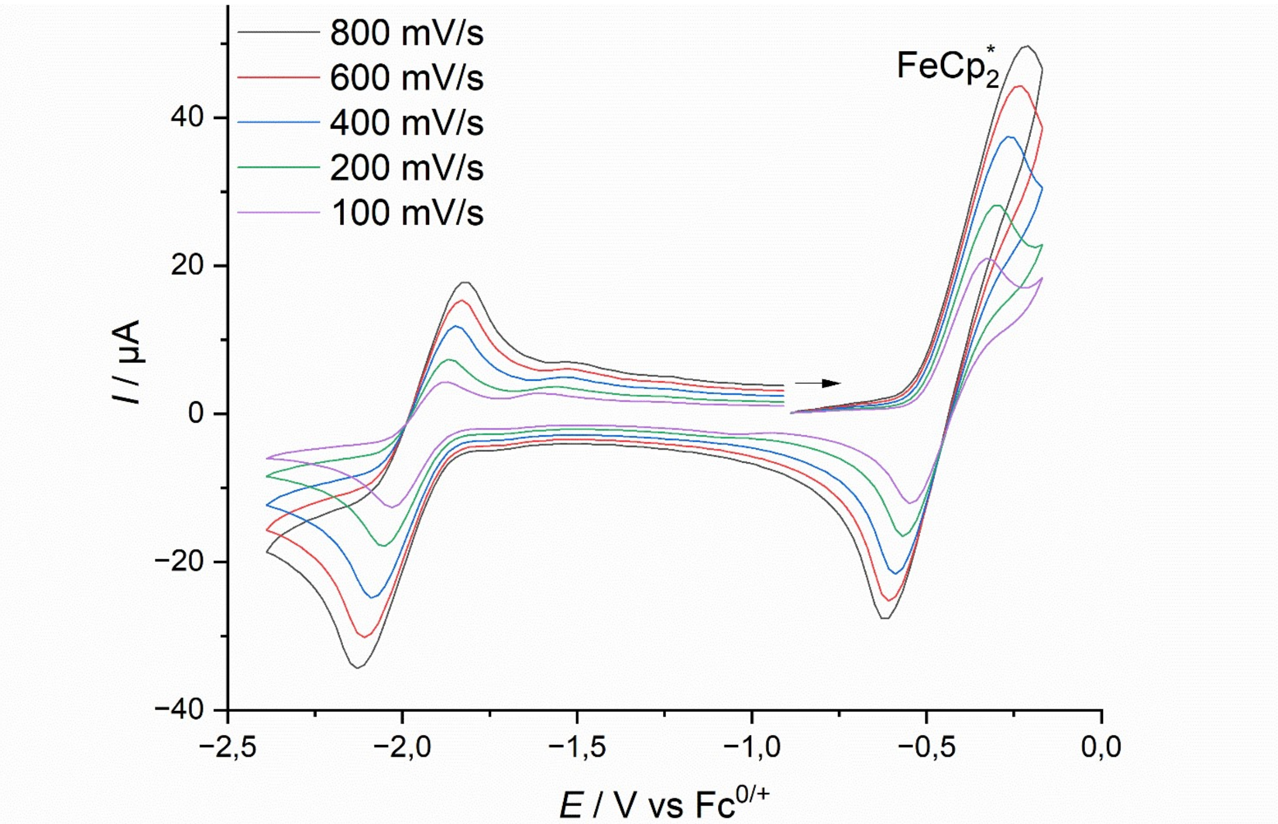

Figure S38. CV of **2** in THF, 0.1 M $[NBu_4][PF_6]$, 1 mM **2**, 1 mM decamethylferrocene, 25 °C.

The cyclic voltammograms of **3** could be recorded without the need of adding an internal reference before addition of **3**. A quasi-reversible reductive event at $E_{1/2}$ = −1.39 V vs $Fc^{0/+}$ and an irreversible oxidative event at E ≈ −0.2 V vs $Fc^{0/+}$ were recorded. After the measurement ferrocene was added to reference the displayed spectrum.

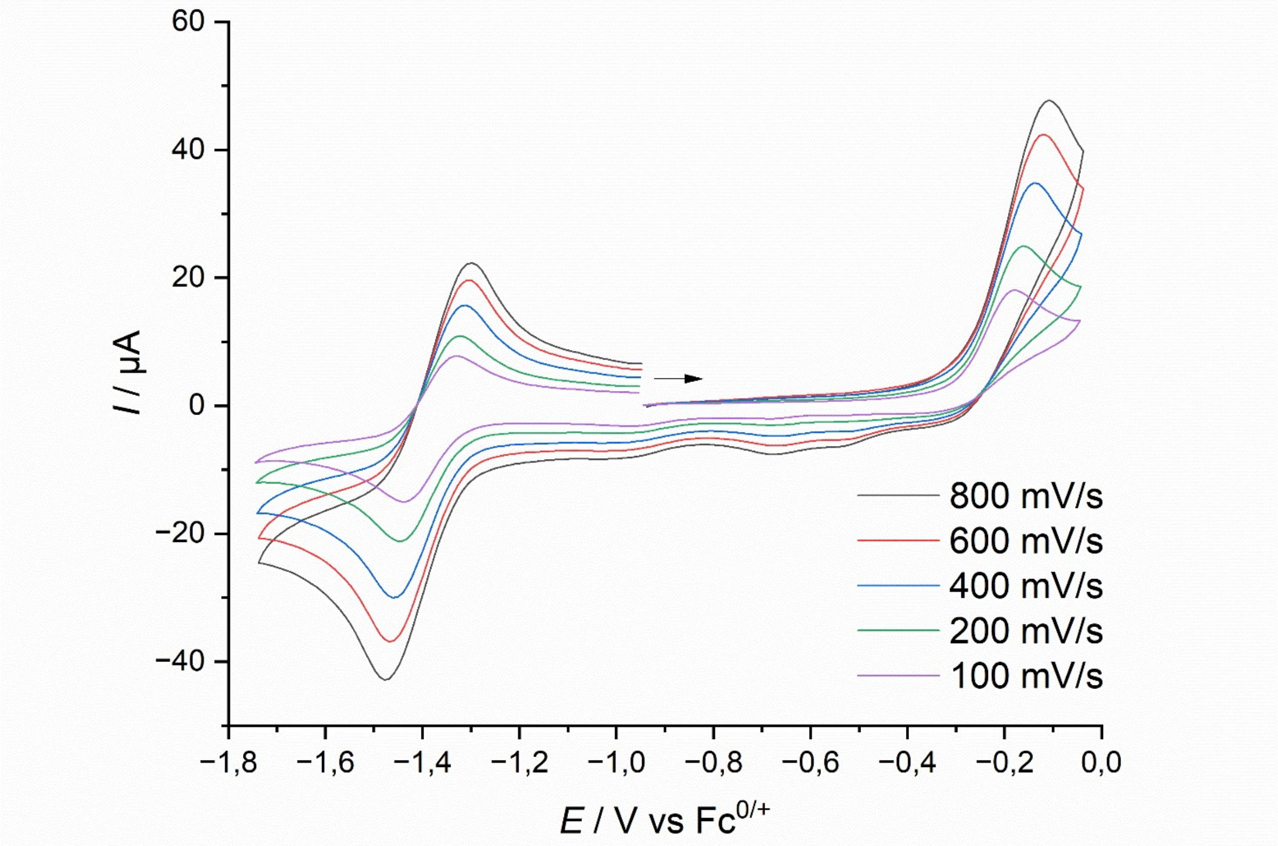

Figure S39. CV of **3** in DCM, 0.1 M $[NBu_4][PF_6]$, 1 mM **3**, 25 °C.

Single Crystal X-ray Diffraction

Crystallographic Details of 1

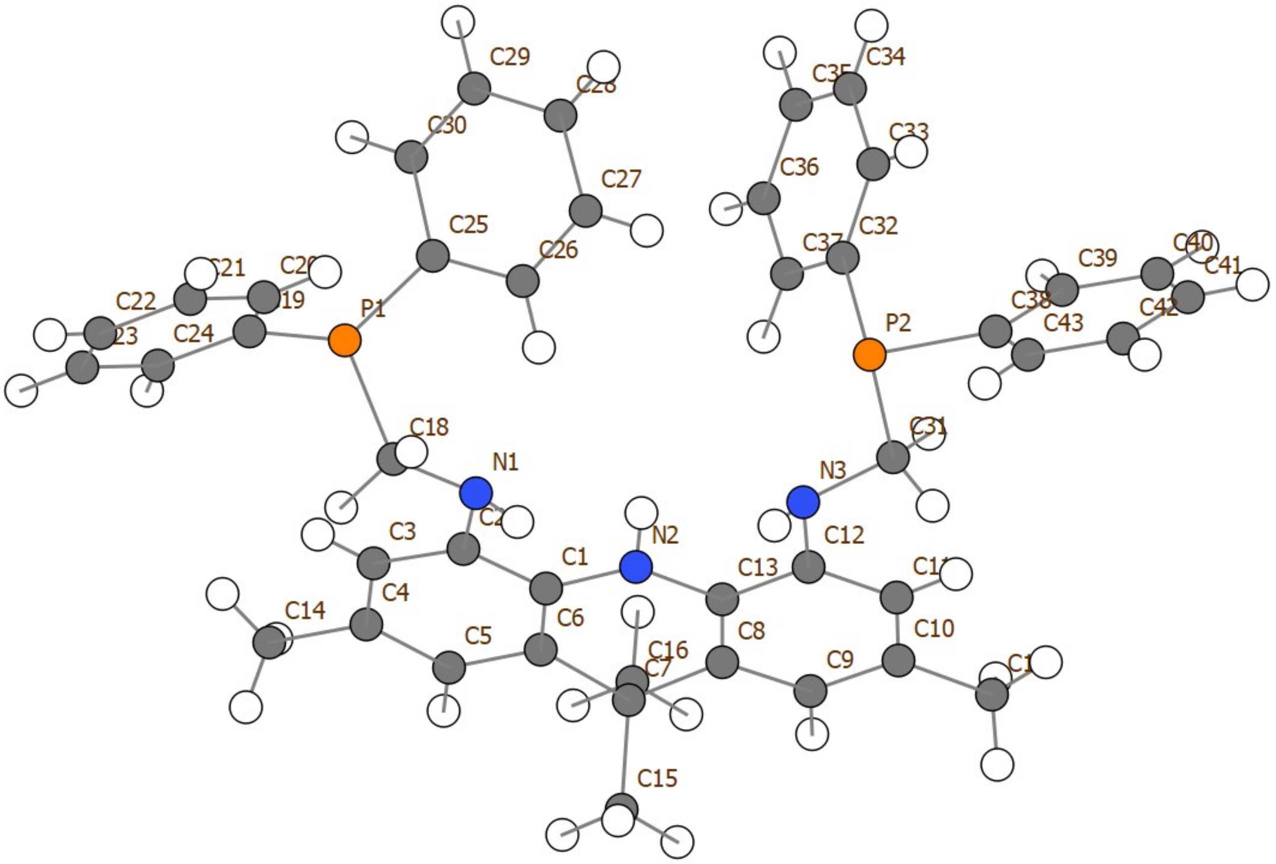

A clear colourless, block-shaped crystal of mo_ja23_117_0m_a (**1**) was mounted on a MiTeGen micromount with perfluoroether oil. Data were collected from a shock-cooled single crystal at 100.0 K on a Bruker D8 VENTURE dual wavelength Mo/Cu three-circle diffractometer with a microfocus sealed X-ray tube using a mirror optics as monochromator and a Bruker PHOTON III detector. The diffractometer was equipped with an Oxford Cryostream 800 low temperature device and used MoK_{α} radiation (λ = 0.71073 Å). All data were integrated with SAINT and a multi-scan absorption correction using SADABS was applied.[S2,S3] The structure was solved by iterative methods using olex2.solve and refined by full-matrix least-squares methods against F^2 by olex2.refine using Olex2.[S4] All non-hydrogen atoms were refined with anisotropic displacement parameters. All hydrogen atoms were refined with isotropic displacement parameters. Some were refined freely and some on calculated positions using a riding model with their U_{iso} values constrained to 1.5 times the U_{eq} of their pivot atoms for terminal sp^3 carbon atoms and 1.2 times for all other carbon atoms. Crystallographic data for the structures reported here have been deposited with the Cambridge Crystallographic Data Centre. CCDC 2320292 contain the supplementary crystallographic data for this paper.[S5] These data can be obtained free of charge from The Cambridge Crystallographic Data Centre via www.ccdc.cam.ac.uk/structures. This report was generated using FinalCif.[S6]

Table S2. Crystal data and structure refinement for **1**.

CCDC number	2320292
Empirical formula	$C_{43}H_{43}N_3P_2$
Formula weight	663.74
Temperature [K]	100.0
Crystal system	monoclinic
Space group (number)	$P2_1/n$ (14)
a [Å]	12.4142(13)
b [Å]	15.1667(13)
c [Å]	18.9994(18)
α [°]	90
β [°]	92.258(4)
γ [°]	90
Volume [Å^3]	3574.5(6)
Z	4
ρ_{calc} [gcm^{-3}]	1.233
μ [mm^{-1}]	0.157
F(000)	1408
Crystal size [mm^3]	0.28×0.18×0.15
Crystal colour	clear colourless
Crystal shape	block
Radiation	MoK_α (λ=0.71073 Å)
2θ range [°]	4.24 to 61.12 (0.70 Å)
Index ranges	−17 ≤ h ≤ 17<br>−21 ≤ k ≤ 21<br>−27 ≤ l ≤ 27
Reflections collected	135972
Independent reflections	10939<br>R_{int} = 0.0556<br>R_{sigma} = 0.0222
Completeness to θ = 25.242°	99.8 %
Data / Restraints / Parameters	10939/0/449
Goodness-of-fit on F^2	1.033
Final *R* indexes [I≥2σ(I)]	R_1 = 0.0393<br>wR_2 = 0.1027
Final *R* indexes [all data]	R_1 = 0.0454<br>wR_2 = 0.1069
Largest peak/hole [eÅ^{-3}]	0.90/-0.46

Table S3. Bond lengths and angles for **1.**

**Atom–Atom**	**Length [Å]**
P1–C18	1.8603(11)
P1–C19	1.8416(12)
P1–C25	1.8307(13)
N1–C2	1.4133(14)
N1–C18	1.4631(14)
C1–N2	1.4012(13)
C1–C2	1.4193(14)
C1–C6	1.3959(15)
P2–C31	1.8587(11)
P2–C32	1.8343(11)
P2–C38	1.8363(11)
N2–C13	1.3985(13)
C2–C3	1.3919(15)
N3–C12	1.4182(14)
N3–C31	1.4614(14)
C3–C4	1.4017(16)
C4–C5	1.3915(15)
C4–C14	1.5112(16)
C5–C6	1.4018(15)
C6–C7	1.5301(14)
C7–C8	1.5267(14)
C7–C15	1.5453(15)
C7–C16	1.5458(15)
C8–C9	1.4049(14)
C8–C13	1.3970(14)
C9–C10	1.3893(15)
C10–C11	1.4005(15)
C10–C17	1.5107(15)
C11–C12	1.3932(14)
C12–C13	1.4156(14)
C19–C20	1.3969(18)
C19–C24	1.3940(17)
C20–C21	1.3959(18)
C21–C22	1.383(2)
C22–C23	1.397(3)
C23–C24	1.3925(19)
C25–C26	1.3898(19)
C25–C30	1.4019(17)
C26–C27	1.3960(19)
C27–C28	1.377(3)
C28–C29	1.378(3)
C29–C30	1.393(2)
C32–C33	1.3998(16)
C32–C37	1.3975(16)
C33–C34	1.3878(17)
C34–C35	1.3773(19)
C35–C36	1.3872(18)
C36–C37	1.3955(17)
C38–C39	1.3997(16)
C38–C43	1.3964(15)
C39–C40	1.3934(17)
C40–C41	1.385(2)
C41–C42	1.385(2)
C42–C43	1.3931(19)

**Atom–Atom–Atom**	**Angle [°]**
C19–P1–C18	95.69(5)
C25–P1–C18	102.07(5)
C25–P1–C19	100.87(6)
C2–N1–C18	120.19(9)
N2–C1–C2	117.64(9)
C6–C1–N2	121.84(9)
C6–C1–C2	120.49(9)
C32–P2–C31	101.17(5)
C32–P2–C38	101.59(5)
C38–P2–C31	97.02(5)
C13–N2–C1	120.21(9)
N1–C2–C1	116.39(9)
C3–C2–N1	124.33(9)
C3–C2–C1	119.26(10)
C12–N3–C31	117.29(9)
C2–C3–C4	120.93(10)
C3–C4–C14	121.39(10)
C5–C4–C3	118.49(10)
C5–C4–C14	120.11(10)
C4–C5–C6	122.30(10)
C1–C6–C5	118.29(10)
C1–C6–C7	121.79(9)
C5–C6–C7	119.91(9)
C6–C7–C15	108.83(9)
C6–C7–C16	109.70(9)
C8–C7–C6	111.62(9)
C8–C7–C15	108.69(9)
C8–C7–C16	109.13(9)
C15–C7–C16	108.81(10)
C9–C8–C7	119.62(9)
C13–C8–C7	121.97(9)
C13–C8–C9	118.41(10)
C10–C9–C8	122.22(10)
C9–C10–C11	118.54(10)
C9–C10–C17	120.62(10)
C11–C10–C17	120.81(10)
C12–C11–C10	120.96(10)
C11–C12–N3	123.46(9)
C11–C12–C13	119.49(10)
C13–C12–N3	117.00(9)
N2–C13–C12	117.87(9)
C8–C13–N2	121.76(9)
C8–C13–C12	120.37(9)
N1–C18–P1	114.03(7)
C20–C19–P1	123.51(9)
C24–C19–P1	118.07(10)
C24–C19–C20	118.35(11)
C21–C20–C19	120.55(13)
C22–C21–C20	120.56(14)
C21–C22–C23	119.48(13)
C24–C23–C22	119.75(14)
C23–C24–C19	121.28(14)
C26–C25–P1	124.79(10)
C26–C25–C30	118.39(12)
C30–C25–P1	116.76(11)
C25–C26–C27	121.05(14)
C28–C27–C26	119.67(17)
C27–C28–C29	120.29(15)
C28–C29–C30	120.37(15)
C29–C30–C25	120.18(16)
N3–C31–P2	111.82(7)
C33–C32–P2	120.15(9)
C37–C32–P2	121.34(9)
C37–C32–C33	118.36(11)
C34–C33–C32	120.53(11)
C35–C34–C33	120.74(11)
C34–C35–C36	119.64(11)
C35–C36–C37	120.13(12)

C36–C37–C32	120.58(11)
C39–C38–P2	123.67(9)
C43–C38–P2	118.16(9)
C43–C38–C39	118.16(11)
C40–C39–C38	121.04(12)
C41–C40–C39	119.94(13)
C42–C41–C40	119.76(12)
C41–C42–C43	120.41(12)
C42–C43–C38	120.67(12)

Bonds to hydrogen atoms were omitted.

Table S4. Torsion angles for **1**.

**Atom–Atom–Atom–Atom**	**Torsion Angle [°]**
P1–C19–C20–C21	−175.73(11)
P1–C19–C24–C23	176.39(10)
P1–C25–C26–C27	178.72(12)
P1–C25–C30–C29	−179.51(10)
N1–C2–C3–C4	−177.39(10)
C1–N2–C13–C8	−7.35(15)
C1–N2–C13–C12	173.47(9)
C1–C2–C3–C4	4.18(16)
C1–C6–C7–C8	−7.80(14)
C1–C6–C7–C15	112.17(11)
C1–C6–C7–C16	−128.89(11)
P2–C32–C33–C34	−177.07(9)
P2–C32–C37–C36	177.01(11)
P2–C38–C39–C40	−179.90(10)
P2–C38–C43–C42	179.76(10)
N2–C1–C2–N1	−2.03(14)
N2–C1–C2–C3	176.52(9)
N2–C1–C6–C5	−179.58(10)
N2–C1–C6–C7	1.61(15)
C2–N1–C18–P1	−87.78(10)
C2–C1–N2–C13	−175.57(9)
C2–C1–C6–C5	2.47(15)
C2–C1–C6–C7	−176.34(9)
C2–C3–C4–C5	0.00(16)
C2–C3–C4–C14	178.72(10)
N3–C12–C13–N2	−2.02(14)
N3–C12–C13–C8	178.80(9)
C3–C4–C5–C6	−3.10(17)
C4–C5–C6–C1	1.85(16)
C4–C5–C6–C7	−179.32(10)
C5–C6–C7–C8	173.42(9)
C5–C6–C7–C15	−66.62(13)
C5–C6–C7–C16	52.32(13)
C6–C1–N2–C13	6.43(15)
C6–C1–C2–N1	176.00(9)
C6–C1–C2–C3	−5.45(15)
C6–C7–C8–C9	−173.19(9)
C6–C7–C8–C13	6.92(13)
C7–C8–C9–C10	179.31(9)
C7–C8–C13–N2	0.21(15)
C7–C8–C13–C12	179.37(9)
C8–C9–C10–C11	1.17(16)
C8–C9–C10–C17	179.14(10)
C9–C8–C13–N2	−179.68(9)
C9–C8–C13–C12	−0.52(15)
C9–C10–C11–C12	−0.22(16)
C10–C11–C12–N3	−178.24(10)
C10–C11–C12–C13	−1.05(15)
C11–C12–C13–N2	−179.39(9)
C11–C12–C13–C8	1.43(15)
C12–N3–C31–P2	167.49(8)
C13–C8–C9–C10	−0.79(15)
C14–C4–C5–C6	178.16(10)
C15–C7–C8–C9	66.77(12)
C15–C7–C8–C13	−113.12(11)
C16–C7–C8–C9	−51.77(13)
C16–C7–C8–C13	128.34(11)
C17–C10–C11–C12	−178.19(10)
C18–P1–C19–C20	83.58(11)
C18–P1–C19–C24	−93.40(10)
C18–P1–C25–C26	11.78(12)
C18–P1–C25–C30	−171.01(9)
C18–N1–C2–C1	−172.57(9)
C18–N1–C2–C3	8.96(15)
C19–P1–C18–N1	−178.82(8)
C19–P1–C25–C26	110.08(11)
C19–P1–C25–C30	−72.71(10)
C19–C20–C21–C22	−0.5(2)
C20–C19–C24–C23	−0.75(19)
C20–C21–C22–C23	−0.8(2)
C21–C22–C23–C24	1.3(2)
C22–C23–C24–C19	−0.5(2)
C24–C19–C20–C21	1.24(19)
C25–P1–C18–N1	−76.40(9)
C25–P1–C19–C20	−19.91(12)
C25–P1–C19–C24	163.11(10)
C25–C26–C27–C28	0.3(2)
C26–C25–C30–C29	−2.12(19)
C26–C27–C28–C29	−1.6(3)
C27–C28–C29–C30	1.0(2)
C28–C29–C30–C25	0.9(2)
C30–C25–C26–C27	1.6(2)
C31–P2–C32–C33	−143.66(9)
C31–P2–C32–C37	40.85(11)
C31–P2–C38–C39	70.09(11)
C31–P2–C38–C43	−108.65(10)
C31–N3–C12–C11	6.29(15)
C31–N3–C12–C13	−170.97(9)
C32–P2–C31–N3	−81.91(8)
C32–P2–C38–C39	−32.89(11)
C32–P2–C38–C43	148.38(9)
C32–C33–C34–C35	0.56(19)
C33–C32–C37–C36	1.44(19)
C33–C34–C35–C36	0.4(2)
C34–C35–C36–C37	−0.4(2)
C35–C36–C37–C32	−0.5(2)
C37–C32–C33–C34	−1.45(18)
C38–P2–C31–N3	174.75(8)
C38–P2–C32–C33	−44.01(10)
C38–P2–C32–C37	140.49(10)
C38–C39–C40–C41	0.5(2)
C39–C38–C43–C42	0.95(18)
C39–C40–C41–C42	0.3(2)
C40–C41–C42–C43	−0.5(2)
C41–C42–C43–C38	−0.1(2)
C43–C38–C39–C40	−1.17(19)

Bonds to hydrogen atoms were omitted.

Crystallographic Details of 2

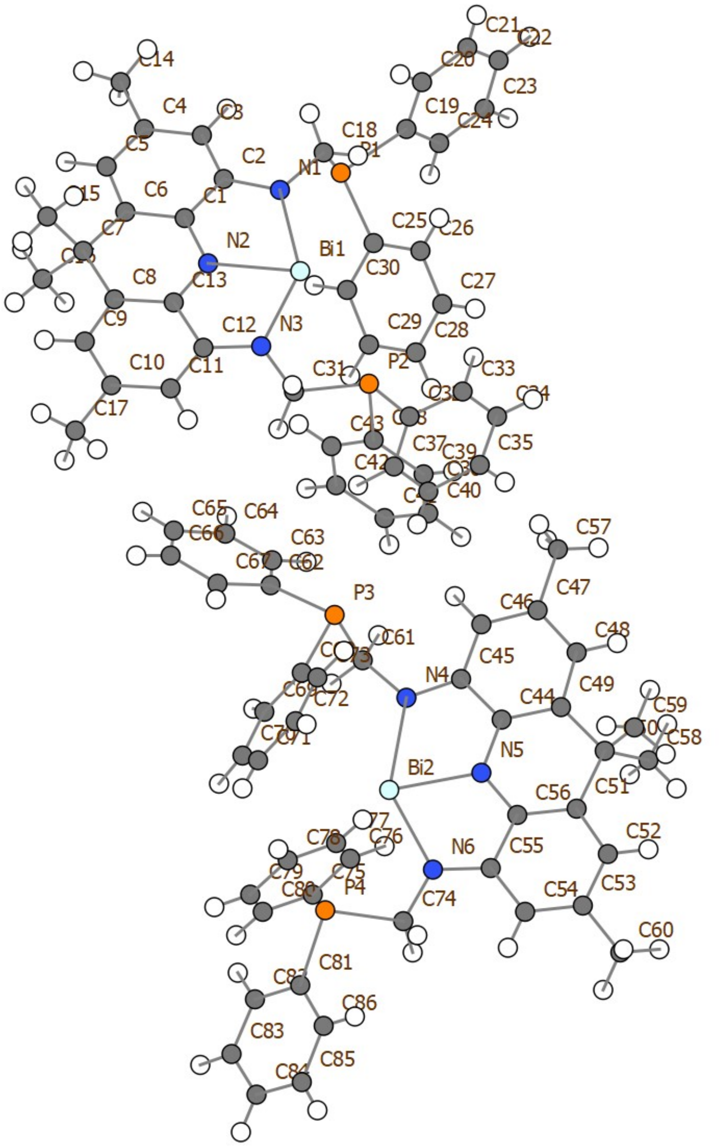

A clear dark violet, plate-shaped crystal of mo_ja23_ja_ii_18_0m_a (**2**) was mounted on a MiTeGen micromount with perfluoroether oil. Data were collected from a shock-cooled single crystal at 100.0 K on a Bruker D8 VENTURE dual wavelength Mo/Cu three-circle diffractometer with a microfocus sealed X-ray tube using a mirror optics as monochromator and a Bruker PHOTON III detector. The diffractometer was equipped with an Oxford Cryostream 800 low temperature device and used MoK_α radiation (λ = 0.71073 Å). All data were integrated with SAINT and a multi-scan absorption correction using SADABS was applied.[S2,S3] The structure was solved by iterative methods using olex2.solve and refined by full-matrix least-squares methods against F^2 by olex2.refine using Olex2.[S4] All non-hydrogen atoms were refined with anisotropic displacement parameters. All hydrogen atoms were refined with isotropic displacement parameters. Some were refined freely and some on calculated positions using a riding model with their U_{iso} values constrained to 1.5 times the U_{eq} of their pivot atoms for terminal sp^3 carbon atoms and 1.2 times for all other carbon atoms. Crystallographic data for the structures reported here have been deposited with the Cambridge Crystallographic Data Centre. CCDC 2320297 contain the supplementary crystallographic data for this paper.[S5] These data can be obtained free of charge from The Cambridge Crystallographic Data Centre via www.ccdc.cam.ac.uk/structures. This report was generated using FinalCif.[S6]

Table S5. Crystal data and structure refinement for **2**.

CCDC number	2320297
Empirical formula	$C_{43}H_{40}BiN_3P_2$
Formula weight	869.745
Temperature [K]	100.00
Crystal system	triclinic
Space group (number)	$P\overline{1}$ (2)
a [Å]	12.5454(10)
b [Å]	14.8524(15)
c [Å]	20.970(2)
α [°]	72.803(4)
β [°]	83.022(3)
γ [°]	86.907(3)
Volume [Å^3]	3704.2(6)
Z	4
ρ_{calc} [gcm^{-3}]	1.560
μ [mm^{-1}]	4.880
F(000)	1716.399
Crystal size [mm^3]	0.07×0.06×0.01
Crystal colour	clear dark violet
Crystal shape	plate
Radiation	Mo K_α (λ=0.71073 Å)
2θ range [°]	3.98 to 56.78 (0.75 Å)
Index ranges	$-16 \le h \le 16$<br>$-19 \le k \le 19$<br>$-27 \le l \le 28$
Reflections collected	153010
Independent reflections	18487<br>R_{int} = 0.0972<br>R_{sigma} = 0.0514
Completeness to θ = 25.2417°	100.0 %
Data / Restraints / Parameters	18487/0/891
Goodness-of-fit on F^2	1.0131
Final *R* indexes [$I \ge 2\sigma(I)$]	R_1 = 0.0367<br>wR_2 = 0.0771
Final *R* indexes [all data]	R_1 = 0.0587<br>wR_2 = 0.0870
Largest peak/hole [eÅ^{-3}]	2.08/-1.65

Table S6. Bond lengths and angles for **2**.

**Atom–Atom**	**Length [Å]**
Bi2–N5	2.180(3)
Bi2–N4	2.296(3)
Bi2–N6	2.307(3)
Bi1–N2	2.186(3)
Bi1–N1	2.287(4)
Bi1–N3	2.290(4)
P4–C75	1.829(5)
P4–C74	1.856(4)
P4–C81	1.833(5)
P2–C31	1.864(4)
P2–C38	1.831(5)
P2–C32	1.819(5)
P3–C61	1.876(4)
P3–C68	1.842(5)
P3–C62	1.835(5)
P1–C25	1.833(5)
P1–C18	1.873(4)
P1–C19	1.837(5)
N2–C1	1.372(6)
N2–C13	1.376(5)
N5–C44	1.371(5)
N5–C56	1.389(5)
N1–C2	1.353(5)
N1–C18	1.440(5)
N4–C45	1.365(5)
N4–C61	1.439(5)
N6–C55	1.355(5)
N6–C74	1.459(5)
N3–C12	1.359(5)
N3–C31	1.459(6)
C12–C13	1.433(6)
C12–C11	1.408(6)
C1–C2	1.424(6)
C1–C6	1.407(6)
C13–C8	1.414(6)
C44–C45	1.431(6)
C44–C49	1.410(6)
C2–C3	1.413(6)
C46–C45	1.406(6)
C46–C47	1.385(6)
C54–C55	1.413(6)
C54–C53	1.380(6)
C55–C56	1.430(6)
C76–C75	1.403(6)
C76–C77	1.379(7)
C75–C80	1.393(6)
C30–C25	1.386(7)
C30–C29	1.387(7)
C56–C51	1.398(6)
C49–C50	1.530(6)
C49–C48	1.383(6)
C11–C10	1.383(6)
C8–C7	1.529(6)
C8–C9	1.393(6)
C47–C48	1.404(6)
C47–C57	1.505(6)
C52–C51	1.384(6)
C52–C53	1.404(6)
C25–C26	1.389(7)
C51–C50	1.529(6)
C50–C59	1.548(6)
C50–C58	1.546(6)
C53–C60	1.510(6)
C77–C78	1.377(7)
C7–C6	1.540(6)
C7–C15	1.532(6)
C7–C16	1.543(6)
C9–C10	1.395(7)
C5–C6	1.382(6)
C5–C4	1.407(6)
C19–C24	1.391(6)
C19–C20	1.399(6)
C68–C69	1.395(6)
C68–C73	1.374(7)
C3–C4	1.385(6)
C81–C86	1.394(6)
C81–C82	1.400(6)
C64–C65	1.377(7)
C64–C63	1.390(6)
C65–C66	1.384(7)
C86–C85	1.395(6)
C4–C14	1.508(7)
C70–C69	1.374(7)
C70–C71	1.388(7)
C43–C38	1.394(6)
C43–C42	1.379(7)
C82–C83	1.385(6)
C26–C27	1.376(7)
C78–C79	1.392(7)
C38–C39	1.400(6)
C83–C84	1.383(7)
C73–C72	1.387(7)
C24–C23	1.390(7)
C66–C67	1.386(7)
C32–C37	1.403(6)
C32–C33	1.390(6)
C80–C79	1.391(7)
C63–C62	1.389(6)
C10–C17	1.516(6)
C67–C62	1.411(6)
C22–C23	1.375(7)
C22–C21	1.391(7)
C20–C21	1.382(7)
C37–C36	1.380(7)
C29–C28	1.399(8)
C71–C72	1.383(8)
C42–C41	1.388(7)
C33–C34	1.385(7)
C28–C27	1.379(8)
C85–C84	1.377(7)
C35–C36	1.392(8)
C35–C34	1.371(8)
C40–C39	1.392(7)
C40–C41	1.385(8)

**Atom–Atom–Atom**	**Angle [°]**
N4–Bi2–N5	71.89(13)
N6–Bi2–N5	71.92(13)
N6–Bi2–N4	143.79(12)
N1–Bi1–N2	71.29(13)
N3–Bi1–N2	72.03(13)

N3–Bi1–N1	143.32(12)	C48–C49–C44	117.5(4)
C74–P4–C75	102.1(2)	C48–C49–C50	121.4(4)
C81–P4–C75	98.6(2)	C10–C11–C12	120.8(4)
C81–P4–C74	100.60(19)	N6–C74–P4	111.0(3)
C38–P2–C31	101.9(2)	C7–C8–C13	121.6(4)
C32–P2–C31	103.5(2)	C9–C8–C13	117.2(4)
C32–P2–C38	100.6(2)	C9–C8–C7	121.1(4)
C68–P3–C61	98.1(2)	N4–C61–P3	110.3(3)
C62–P3–C61	101.1(2)	C48–C47–C46	119.0(4)
C62–P3–C68	100.7(2)	C57–C47–C46	119.9(4)
C18–P1–C25	98.8(2)	C57–C47–C48	121.1(4)
C19–P1–C25	100.5(2)	C53–C52–C51	122.1(4)
C19–P1–C18	101.16(19)	C30–C25–P1	118.6(4)
C1–N2–Bi1	119.7(3)	C26–C25–P1	122.7(4)
C13–N2–Bi1	119.2(3)	C26–C25–C30	118.7(5)
C13–N2–C1	121.0(4)	C52–C51–C56	118.2(4)
C44–N5–Bi2	119.9(3)	C50–C51–C56	121.0(4)
C56–N5–Bi2	119.6(3)	C50–C51–C52	120.8(4)
C56–N5–C44	120.4(3)	N3–C31–P2	107.7(3)
C2–N1–Bi1	117.4(3)	C51–C50–C49	112.1(3)
C18–N1–Bi1	121.8(3)	C59–C50–C49	108.8(4)
C18–N1–C2	120.8(4)	C59–C50–C51	108.2(4)
C45–N4–Bi2	116.2(3)	C58–C50–C49	108.5(4)
C61–N4–Bi2	124.0(3)	C58–C50–C51	109.5(4)
C61–N4–C45	119.8(3)	C58–C50–C59	109.7(4)
C55–N6–Bi2	116.1(3)	C52–C53–C54	119.5(4)
C74–N6–Bi2	125.3(3)	C60–C53–C54	120.4(4)
C74–N6–C55	118.5(4)	C60–C53–C52	120.1(4)
C12–N3–Bi1	116.5(3)	C78–C77–C76	120.8(5)
C31–N3–Bi1	124.0(3)	C47–C48–C49	122.8(4)
C31–N3–C12	119.3(4)	C6–C7–C8	111.4(3)
C13–C12–N3	115.7(4)	C15–C7–C8	109.3(4)
C11–C12–N3	126.4(4)	C15–C7–C6	108.7(4)
C11–C12–C13	117.9(4)	C16–C7–C8	108.3(4)
C2–C1–N2	116.2(4)	C16–C7–C6	110.0(4)
C6–C1–N2	122.7(4)	C16–C7–C15	109.1(4)
C6–C1–C2	121.1(4)	C10–C9–C8	122.4(4)
C12–C13–N2	116.3(4)	C4–C5–C6	122.4(4)
C8–C13–N2	122.0(4)	N1–C18–P1	110.7(3)
C8–C13–C12	121.6(4)	C7–C6–C1	121.1(4)
C45–C44–N5	116.1(4)	C5–C6–C1	118.1(4)
C49–C44–N5	122.5(4)	C5–C6–C7	120.8(4)
C49–C44–C45	121.4(4)	C24–C19–P1	116.8(4)
C1–C2–N1	115.3(4)	C20–C19–P1	124.4(3)
C3–C2–N1	126.4(4)	C20–C19–C24	118.5(4)
C3–C2–C1	118.3(4)	C69–C68–P3	121.9(4)
C47–C46–C45	121.1(4)	C73–C68–P3	118.3(4)
C53–C54–C55	120.9(4)	C73–C68–C69	119.8(4)
C54–C55–N6	125.7(4)	C4–C3–C2	120.8(4)
C56–C55–N6	116.4(4)	C86–C81–P4	122.2(3)
C56–C55–C54	117.8(4)	C82–C81–P4	119.1(3)
C44–C45–N4	115.8(4)	C82–C81–C86	118.6(4)
C46–C45–N4	126.2(4)	C63–C64–C65	120.6(5)
C46–C45–C44	118.0(4)	C66–C65–C64	119.6(4)
C77–C76–C75	120.9(5)	C85–C86–C81	120.4(4)
C76–C75–P4	122.0(3)	C3–C4–C5	119.2(4)
C80–C75–P4	119.7(4)	C14–C4–C5	120.7(4)
C80–C75–C76	117.9(4)	C14–C4–C3	120.0(4)
C29–C30–C25	120.3(5)	C71–C70–C69	120.0(5)
C55–C56–N5	115.7(4)	C42–C43–C38	121.2(4)
C51–C56–N5	122.8(4)	C70–C69–C68	120.2(5)
C51–C56–C55	121.4(4)	C83–C82–C81	120.7(4)
C50–C49–C44	121.1(4)	C27–C26–C25	121.0(5)

C79–C78–C77	119.3(4)
C43–C38–P2	120.2(3)
C39–C38–P2	121.2(4)
C39–C38–C43	118.2(4)
C84–C83–C82	119.9(4)
C72–C73–C68	120.1(5)
C23–C24–C19	120.5(5)
C67–C66–C65	120.4(5)
C37–C32–P2	123.6(4)
C33–C32–P2	117.4(4)
C33–C32–C37	118.8(4)
C79–C80–C75	120.9(5)
C62–C63–C64	120.8(4)
C9–C10–C11	120.1(4)
C17–C10–C11	120.7(4)
C17–C10–C9	119.1(4)
C62–C67–C66	120.6(5)
C21–C22–C23	118.7(5)
C21–C20–C19	120.3(4)
C36–C37–C32	120.5(5)
C63–C62–P3	121.3(3)
C67–C62–P3	120.4(4)
C67–C62–C63	118.0(4)
C28–C29–C30	120.6(5)
C72–C71–C70	119.8(5)
C80–C79–C78	120.2(5)
C41–C42–C43	119.9(5)
C71–C72–C73	120.1(5)
C22–C23–C24	121.0(5)
C20–C21–C22	120.9(5)
C34–C33–C32	120.6(5)
C27–C28–C29	118.5(5)
C84–C85–C86	120.0(5)
C85–C84–C83	120.4(5)
C34–C35–C36	120.5(5)
C41–C40–C39	119.6(5)
C40–C39–C38	120.8(5)
C35–C36–C37	119.6(5)
C28–C27–C26	120.8(5)
C35–C34–C33	120.1(5)
C40–C41–C42	120.2(5)

Bonds to hydrogen atoms were omitted.

Table S7. Torsion angles for **2**.

Atom–Atom–Atom–Atom	Torsion Angle [°]
Bi2–N5–C44–C45	0.4(3)
Bi2–N5–C44–C49	−178.8(3)
Bi2–N5–C56–C55	−3.3(3)
Bi2–N5–C56–C51	177.4(3)
Bi2–N4–C45–C44	3.8(3)
Bi2–N4–C45–C46	−175.4(3)
Bi2–N4–C61–P3	92.3(2)
Bi2–N6–C55–C54	−177.7(3)
Bi2–N6–C55–C56	3.2(3)
Bi2–N6–C74–P4	−14.0(3)
Bi1–N2–C1–C2	−0.6(3)
Bi1–N2–C1–C6	179.8(3)
Bi1–N2–C13–C12	−2.0(3)
Bi1–N2–C13–C8	177.3(3)
Bi1–N1–C2–C1	3.6(3)
Bi1–N1–C2–C3	−175.5(3)
Bi1–N1–C18–P1	94.5(2)
Bi1–N3–C12–C13	4.0(3)
Bi1–N3–C12–C11	−177.1(3)
Bi1–N3–C31–P2	−14.7(3)
P4–C75–C76–C77	173.2(4)
P4–C75–C80–C79	−173.2(4)
P4–C74–N6–C55	170.0(3)
P4–C81–C86–C85	177.0(4)
P4–C81–C82–C83	−176.2(3)
P2–C31–N3–C12	169.8(3)
P2–C38–C43–C42	173.6(4)
P2–C38–C39–C40	−173.9(4)
P2–C32–C37–C36	175.9(4)
P2–C32–C33–C34	−175.3(4)
P3–C61–N4–C45	−85.2(3)
P3–C68–C69–C70	177.3(4)
P3–C68–C73–C72	−178.7(4)
P3–C62–C63–C64	−174.2(4)
P3–C62–C67–C66	174.4(4)
P1–C25–C30–C29	−177.8(4)
P1–C25–C26–C27	177.9(4)
P1–C18–N1–C2	−83.2(3)
P1–C19–C24–C23	173.3(4)
P1–C19–C20–C21	−171.0(4)
N2–C1–C2–N1	−2.0(4)
N2–C1–C2–C3	177.2(4)
N2–C1–C6–C7	3.7(5)
N2–C1–C6–C5	−177.8(4)
N2–C13–C12–N3	−1.4(4)
N2–C13–C12–C11	179.6(4)
N2–C13–C8–C7	2.0(5)
N2–C13–C8–C9	−179.7(4)
N5–C44–C45–N4	−2.8(4)
N5–C44–C45–C46	176.5(4)
N5–C44–C49–C50	0.6(5)
N5–C44–C49–C48	−179.0(4)
N5–C56–C55–N6	−0.1(4)
N5–C56–C55–C54	−179.3(4)
N5–C56–C51–C52	179.8(4)
N5–C56–C51–C50	2.1(5)
N1–C2–C1–C6	177.6(4)
N1–C2–C3–C4	179.9(5)
N4–C45–C44–C49	176.3(4)
N4–C45–C46–C47	−177.9(4)
N6–C55–C54–C53	−179.3(4)
N6–C55–C56–C51	179.2(4)
N3–C12–C13–C8	179.3(4)
N3–C12–C11–C10	−178.6(4)
C12–C13–C8–C7	−178.7(4)
C12–C13–C8–C9	−0.5(5)
C12–C11–C10–C9	−0.6(5)
C12–C11–C10–C17	177.1(4)
C1–C2–C3–C4	0.8(5)
C1–C6–C7–C8	−3.4(5)
C1–C6–C7–C15	117.1(5)
C1–C6–C7–C16	−123.5(5)
C1–C6–C5–C4	0.4(5)
C13–C8–C7–C6	0.7(4)
C13–C8–C7–C15	−119.4(4)
C13–C8–C7–C16	121.8(4)
C13–C8–C9–C10	0.1(5)

C44–C45–C46–C47	2.9(5)
C44–C49–C50–C51	0.2(4)
C44–C49–C50–C59	−119.4(4)
C44–C49–C50–C58	121.3(4)
C44–C49–C48–C47	2.1(5)
C2–C3–C4–C5	2.1(5)
C2–C3–C4–C14	−176.8(4)
C46–C47–C48–C49	−3.6(5)
C54–C55–C56–C51	0.0(5)
C54–C53–C52–C51	0.8(5)
C55–C56–C51–C52	0.6(5)
C55–C56–C51–C50	−177.2(4)
C76–C75–C80–C79	0.1(5)
C76–C77–C78–C79	−0.1(6)
C75–C80–C79–C78	−0.2(6)
C30–C25–C26–C27	0.3(5)
C30–C29–C28–C27	1.0(6)
C56–C51–C52–C53	−1.0(5)
C56–C51–C50–C49	−1.5(4)
C56–C51–C50–C59	118.5(4)
C56–C51–C50–C58	−122.0(4)
C49–C50–C51–C52	−179.2(4)
C49–C48–C47–C57	175.0(4)
C11–C10–C9–C8	0.4(5)
C8–C7–C6–C5	178.2(4)
C8–C9–C10–C17	−177.3(4)
C52–C51–C50–C59	−59.2(4)
C52–C51–C50–C58	60.3(5)
C25–C26–C27–C28	0.1(5)
C77–C78–C79–C80	0.3(6)
C7–C6–C5–C4	178.9(4)
C19–C24–C23–C22	−1.6(6)
C19–C20–C21–C22	−2.0(6)
C68–C69–C70–C71	2.3(5)
C68–C73–C72–C71	0.3(6)
C81–C86–C85–C84	−1.4(5)
C81–C82–C83–C84	−0.5(5)
C64–C65–C66–C67	0.8(6)
C64–C63–C62–C67	−0.1(6)
C65–C66–C67–C62	−0.6(6)
C86–C85–C84–C83	1.1(6)
C70–C71–C72–C73	1.1(6)
C43–C38–C39–C40	−0.5(5)
C43–C42–C41–C40	0.7(6)
C82–C83–C84–C85	−0.1(6)
C26–C27–C28–C29	−0.7(6)
C38–C39–C40–C41	0.9(6)
C24–C23–C22–C21	2.1(7)
C66–C67–C62–C63	0.3(6)
C32–C37–C36–C35	−1.3(6)
C32–C33–C34–C35	−0.6(6)
C37–C36–C35–C34	0.7(7)
C42–C41–C40–C39	−1.0(6)
C33–C34–C35–C36	0.3(7)

Bonds to hydrogen atoms were omitted.

Crystallographic Details of 3

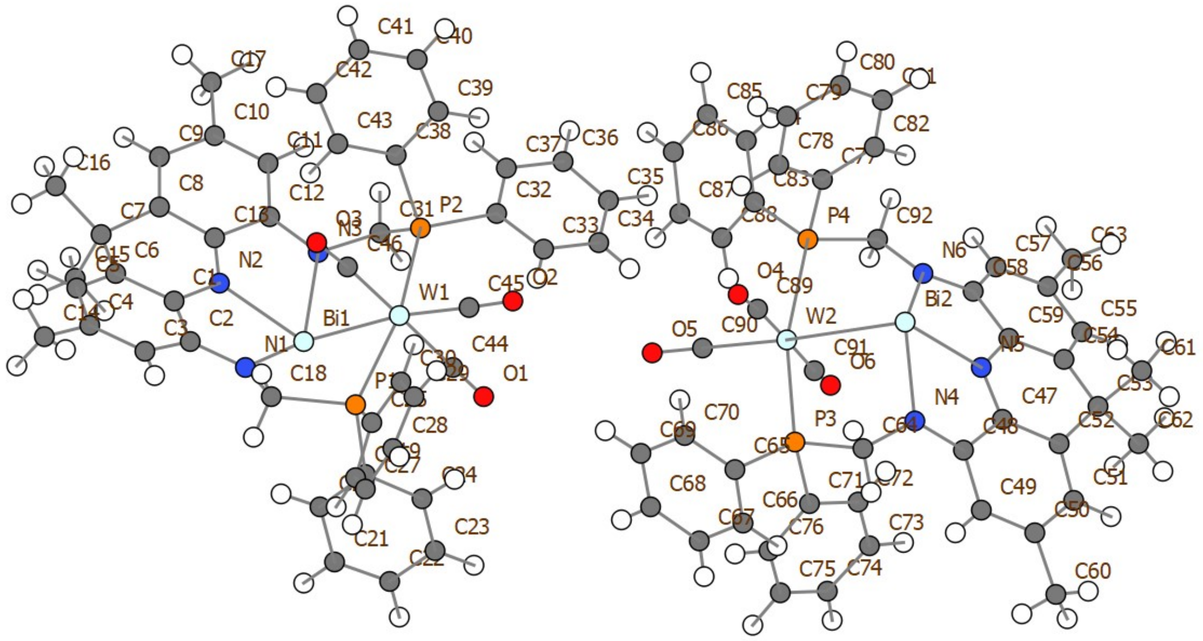

A clear dark blue, block-shaped crystal of mo_ja23_JA_i_85_0m_a (**3**) was mounted on a MiTeGen micromount with perfluoroether oil. Data were collected from a shock-cooled single crystal at 100.0 K on a Bruker D8 VENTURE dual wavelength Mo/Cu three-circle diffractometer with a microfocus sealed X-ray tube using a mirror optics as monochromator and a Bruker PHOTON III detector. The diffractometer was equipped with an Oxford Cryostream 800 low temperature device and used MoK_{α} radiation (λ = 0.71073 Å). All data were integrated with SAINT and a multi-scan absorption correction using SADABS was applied.[S2,S3] The structure was solved by iterative methods using olex2.solve and refined by full-matrix least-squares methods against F^2 by olex2.refine using Olex2.[S4] All non-hydrogen atoms were refined with anisotropic displacement parameters. All hydrogen atoms were refined with isotropic displacement parameters. Some were refined freely and some on calculated positions using a riding model with their U_{iso} values constrained to 1.5 times the U_{eq} of their pivot atoms for terminal sp^3 carbon atoms and 1.2 times for all other carbon atoms. Crystallographic data for the structures reported here have been deposited with the Cambridge Crystallographic Data Centre. CCDC 2320304 contain the supplementary crystallographic data for this paper.[S5] These data can be obtained free of charge from The Cambridge Crystallographic Data Centre via www.ccdc.cam.ac.uk/structures. This report was generated using FinalCif.[S6]

Table S8. Crystal data and structure refinement for **3**.

CCDC number	2320304
Empirical formula	$C_{106}H_{96}Bi_2N_6O_6P_4W_2$
Formula weight	1229.760
Temperature [K]	100.0
Crystal system	triclinic
Space group (number)	$P\overline{1}$ (2)
a [Å]	10.9439(7)
b [Å]	21.0052(10)
c [Å]	21.9721(12)
α [°]	92.010(2)
β [°]	101.049(2)
γ [°]	103.267(2)
Volume [Å^3]	4808.4(5)
Z	4
ρ_{calc} [gcm^{-3}]	1.699
μ [mm^{-1}]	6.157
F(000)	2379.248
Crystal size [mm^3]	0.08×0.07×0.02
Crystal colour	clear dark blue
Crystal shape	block
Radiation	Mo K_α (λ=0.71073 Å)
2θ range [°]	3.94 to 50.86 (0.83 Å)
Index ranges	−13 ≤ h ≤ 13<br>−23 ≤ k ≤ 25<br>−26 ≤ l ≤ 26
Reflections collected	58362
Independent reflections	17672<br>R_{int} = 0.1025<br>R_{sigma} = 0.1049
Completeness to θ = 25.2417°	99.8 %
Data / Restraints / Parameters	17672/2/987
Goodness-of-fit on F^2	0.9731
Final R indexes [I≥2σ(I)]	R_1 = 0.0515<br>wR_2 = 0.1138
Final R indexes [all data]	R_1 = 0.0910<br>wR_2 = 0.1334
Largest peak/hole [eÅ^{-3}]	3.59/-1.80

Table S9. Bond lengths and angles for **3**.

Atom–Atom	Length [Å]	Atom–Atom	Length [Å]
Bi2–W2	2.8049(6)	C58–C57	1.415(14)
Bi2–N6	2.368(8)	C11–C10	1.365(14)
Bi2–N5	2.264(8)	C83–C88	1.375(16)
Bi2–N4	2.303(8)	C83–C84	1.348(15)
Bi1–W1	2.8059(6)	C19–C20	1.37(2)
Bi1–N3	2.328(8)	C19–C24	1.39(2)
Bi1–N1	2.346(8)	C8–C13	1.407(14)
Bi1–N2	2.256(8)	C8–C7	1.552(15)
W1–P2	2.453(3)	C8–C9	1.363(15)
W1–P1	2.460(3)	C47–C52	1.424(14)
W1–C46	2.026(12)	C33–C34	1.356(17)
W1–C44	2.006(12)	C33–C32	1.362(15)
W1–C45	1.892(13)	C71–C72	1.370(12)
W2–P4	2.455(3)	C71–C76	1.382(12)
W2–P3	2.451(3)	C52–C53	1.504(16)
W2–C90	1.977(13)	C52–C51	1.375(16)
W2–C89	2.004(13)	C43–C42	1.389(16)
W2–C91	2.032(13)	C43–C38	1.352(14)
P4–C92	1.854(10)	C53–C62	1.505(15)
P4–C83	1.842(11)	C53–C61	1.605(16)
P4–C77	1.815(10)	C13–N2	1.333(13)
P2–C31	1.887(10)	C51–C50	1.384(16)
P2–C38	1.851(11)	C7–C6	1.506(16)
P2–C32	1.840(11)	C7–C16	1.499(14)
P3–C64	1.900(10)	C7–C15	1.558(16)
P3–C71	1.834(11)	C50–C49	1.359(15)
P3–C65	1.851(12)	C50–C60	1.542(15)
P1–C25	1.816(12)	C57–C56	1.349(14)
P1–C19	1.804(14)	C10–C9	1.409(15)
P1–C18	1.866(11)	C10–C17	1.522(15)
N6–C92	1.443(13)	C86–C87	1.40(2)
N6–C58	1.327(12)	C86–C85	1.39(2)
N3–C12	1.329(12)	C79–C78	1.414(15)
N3–C31	1.435(12)	C79–C80	1.375(17)
N1–C2	1.332(13)	C2–C3	1.439(15)
N1–C18	1.451(13)	C88–C87	1.390(17)
N5–C59	1.353(13)	C82–C81	1.379(17)
N5–C47	1.339(13)	C82–C77	1.372(15)
N4–C48	1.330(12)	C72–C73	1.353(16)
N4–C64	1.471(12)	O6–C91	1.112(13)
O5–C90	1.178(14)	C65–C70	1.361(16)
O4–C89	1.170(13)	C65–C66	1.399(18)
O3–C46	1.121(12)	C6–C5	1.384(16)
O1–C44	1.141(12)	C70–C69	1.397(18)
C25–C30	1.388(17)	C45–O2	1.188(14)
C25–C26	1.395(17)	C3–C4	1.348(16)
C1–C2	1.432(15)	C76–C75	1.369(18)
C1–C6	1.418(14)	C20–C21	1.466(17)
C1–N2	1.360(13)	C78–C77	1.385(14)
C48–C47	1.415(15)	C56–C55	1.438(16)
C48–C49	1.432(14)	C56–C63	1.486(16)
C12–C11	1.420(15)	C37–C36	1.409(16)
C12–C13	1.450(14)	C37–C32	1.387(16)
C54–C59	1.428(14)	C39–C40	1.388(19)
C54–C53	1.516(15)	C39–C38	1.369(16)
C54–C55	1.359(15)	C42–C41	1.38(2)
C59–C58	1.444(14)	C4–C5	1.417(18)
		C4–C14	1.488(16)

C80–C81	1.321(18)
C84–C85	1.346(18)
C27–C28	1.35(2)
C27–C26	1.354(18)
C69–C68	1.40(2)
C24–C23	1.377(17)
C73–C74	1.40(2)
C75–C74	1.31(2)
C34–C35	1.37(2)
C29–C28	1.33(2)
C29–C30	1.430(19)
C36–C35	1.38(2)
C23–C22	1.34(2)
C68–C67	1.33(2)
C40–C41	1.34(2)
C22–C21	1.31(2)
C66–C67	1.368(19)

**Atom–Atom–Atom**	**Angle [°]**
N6–Bi2–W2	87.6(2)
N5–Bi2–W2	118.8(2)
N5–Bi2–N6	69.4(3)
N4–Bi2–W2	91.8(2)
N4–Bi2–N6	132.0(3)
N4–Bi2–N5	69.2(3)
N3–Bi1–W1	91.8(2)
N1–Bi1–W1	88.6(2)
N1–Bi1–N3	133.3(3)
N2–Bi1–W1	118.8(2)
N2–Bi1–N3	69.6(3)
N2–Bi1–N1	69.6(3)
P2–W1–Bi1	86.59(7)
P1–W1–Bi1	79.71(7)
P1–W1–P2	165.90(10)
C46–W1–Bi1	93.1(3)
C46–W1–P2	92.2(3)
C46–W1–P1	85.3(3)
C44–W1–Bi1	88.7(3)
C44–W1–P2	90.0(4)
C44–W1–P1	93.0(3)
C44–W1–C46	177.2(5)
C45–W1–Bi1	173.0(4)
C45–W1–P2	95.5(4)
C45–W1–P1	98.5(4)
C45–W1–C46	93.5(5)
C45–W1–C44	84.6(5)
P4–W2–Bi2	78.07(7)
P3–W2–Bi2	86.53(7)
P3–W2–P4	164.37(10)
C90–W2–Bi2	176.5(3)
C90–W2–P4	98.6(3)
C90–W2–P3	96.8(3)
C89–W2–Bi2	89.4(3)
C89–W2–P4	93.2(3)
C89–W2–P3	89.3(3)
C89–W2–C90	89.8(4)
C91–W2–Bi2	91.6(4)
C91–W2–P4	85.9(3)
C91–W2–P3	91.9(3)
C91–W2–C90	89.1(5)
C91–W2–C89	178.5(5)
C92–P4–W2	110.4(3)
C83–P4–W2	121.3(4)
C83–P4–C92	100.1(5)
C77–P4–W2	118.0(4)
C77–P4–C92	102.3(5)
C77–P4–C83	101.8(5)
C31–P2–W1	112.8(3)
C38–P2–W1	115.8(4)
C38–P2–C31	102.8(5)
C32–P2–W1	121.8(4)
C32–P2–C31	97.9(5)
C32–P2–C38	102.8(5)
C64–P3–W2	113.3(3)
C71–P3–W2	116.3(4)
C71–P3–C64	101.3(5)
C65–P3–W2	119.7(4)
C65–P3–C64	98.5(5)
C65–P3–C71	104.8(5)
C25–P1–W1	122.3(4)
C19–P1–W1	115.4(5)
C19–P1–C25	102.6(6)
C18–P1–W1	111.6(4)
C18–P1–C25	98.8(5)
C18–P1–C19	103.5(7)
C92–N6–Bi2	121.4(6)
C58–N6–Bi2	116.6(7)
C58–N6–C92	120.5(9)
C12–N3–Bi1	116.7(7)
C31–N3–Bi1	117.1(6)
C31–N3–C12	124.7(9)
C2–N1–Bi1	116.0(7)
C18–N1–Bi1	121.0(7)
C18–N1–C2	121.5(9)
C59–N5–Bi2	117.7(7)
C47–N5–Bi2	117.6(7)
C47–N5–C59	123.9(9)
C48–N4–Bi2	117.4(7)
C64–N4–Bi2	118.1(6)
C64–N4–C48	123.7(9)
N6–C92–P4	110.1(7)
O3–C46–W1	174.6(11)
C30–C25–P1	120.4(9)
C26–C25–P1	122.0(10)
C26–C25–C30	117.5(12)
C6–C1–C2	124.7(10)
N2–C1–C2	114.2(9)
N2–C1–C6	120.6(11)
C47–C48–N4	116.3(9)
C49–C48–N4	126.7(11)
C49–C48–C47	117.0(10)
O5–C90–W2	177.7(11)
N4–C64–P3	108.3(6)
C11–C12–N3	126.1(10)
C13–C12–N3	116.8(10)
C13–C12–C11	117.1(9)
C53–C54–C59	118.8(10)
C55–C54–C59	115.9(10)
C55–C54–C53	125.2(9)
C54–C59–N5	120.2(10)
C58–C59–N5	116.2(9)
C58–C59–C54	122.9(10)

C59–C58–N6	116.1(9)	C73–C72–C71	122.7(13)
C57–C58–N6	127.9(10)	C70–C65–P3	118.8(10)
C57–C58–C59	116.0(9)	C66–C65–P3	120.8(10)
C10–C11–C12	120.1(10)	C66–C65–C70	120.3(12)
C88–C83–P4	118.2(9)	C7–C6–C1	120.4(10)
C84–C83–P4	123.3(10)	C5–C6–C1	114.2(11)
C84–C83–C88	118.5(11)	C5–C6–C7	125.4(11)
C20–C19–P1	125.2(13)	C69–C70–C65	120.4(14)
C24–C19–P1	118.6(13)	O2–C45–W1	179.5(13)
C24–C19–C20	116.2(14)	C4–C3–C2	122.0(12)
C7–C8–C13	117.2(10)	C75–C76–C71	119.9(14)
C9–C8–C13	119.3(10)	C21–C20–C19	120.6(16)
C9–C8–C7	123.4(9)	C77–C78–C79	120.3(10)
C48–C47–N5	115.7(9)	C55–C56–C57	118.8(11)
C52–C47–N5	121.2(10)	C63–C56–C57	121.4(11)
C52–C47–C48	122.8(10)	C63–C56–C55	119.8(10)
C32–C33–C34	121.8(14)	C32–C37–C36	119.6(13)
C72–C71–P3	119.9(9)	C1–N2–Bi1	118.8(7)
C76–C71–P3	123.3(10)	C13–N2–Bi1	119.2(7)
C76–C71–C72	116.6(12)	C13–N2–C1	121.4(9)
C53–C52–C47	118.9(10)	C38–C39–C40	118.6(13)
C51–C52–C47	116.2(11)	C41–C42–C43	116.2(13)
C51–C52–C53	124.8(10)	C56–C55–C54	123.6(10)
C38–C43–C42	123.9(13)	O6–C91–W2	176.7(11)
C52–C53–C54	112.4(9)	C5–C4–C3	119.3(11)
C62–C53–C54	112.9(10)	C14–C4–C3	120.7(13)
C62–C53–C52	113.8(10)	C14–C4–C5	120.0(12)
C61–C53–C54	104.5(9)	C81–C80–C79	119.6(12)
C61–C53–C52	105.2(10)	C85–C84–C83	123.1(15)
C61–C53–C62	107.2(10)	C26–C27–C28	122.7(15)
C8–C13–C12	120.8(10)	C68–C69–C70	117.1(15)
N2–C13–C12	114.8(9)	C23–C24–C19	122.4(17)
N2–C13–C8	124.3(10)	C74–C73–C72	119.2(14)
C50–C51–C52	122.3(11)	C74–C75–C76	123.4(15)
O4–C89–W2	175.5(10)	C75–C74–C73	118.1(14)
C6–C7–C8	110.3(9)	C4–C5–C6	124.4(11)
C16–C7–C8	110.8(10)	C35–C34–C33	120.4(15)
C16–C7–C6	111.6(10)	C30–C29–C28	119.3(15)
C15–C7–C8	106.7(9)	C29–C28–C27	120.4(14)
C15–C7–C6	106.1(10)	C29–C30–C25	120.1(13)
C15–C7–C16	111.2(10)	C27–C26–C25	119.8(14)
C49–C50–C51	121.9(10)	C80–C81–C82	122.2(13)
C60–C50–C51	118.6(11)	C35–C36–C37	119.2(15)
C60–C50–C49	119.5(11)	C22–C23–C24	120.0(17)
C56–C57–C58	122.7(11)	C67–C68–C69	122.8(15)
C9–C10–C11	121.5(10)	C36–C35–C34	119.9(12)
C17–C10–C11	119.9(11)	C41–C40–C39	122.0(15)
C17–C10–C9	118.6(10)	C21–C22–C23	121.9(15)
C85–C86–C87	123.7(14)	C82–C77–P4	123.6(8)
C80–C79–C78	119.3(11)	C78–C77–P4	118.8(8)
O1–C44–W1	179.4(11)	C78–C77–C82	117.6(10)
N3–C31–P2	111.0(7)	C43–C38–P2	119.7(9)
C1–C2–N1	117.9(9)	C39–C38–P2	121.6(9)
C3–C2–N1	126.8(10)	C39–C38–C43	118.5(12)
C3–C2–C1	115.3(10)	C33–C32–P2	120.2(10)
C87–C88–C83	123.3(12)	C37–C32–P2	120.6(9)
C77–C82–C81	120.8(12)	C37–C32–C33	119.0(11)
C10–C9–C8	120.9(10)	C67–C66–C65	119.6(15)
N1–C18–P1	112.3(7)	C84–C85–C86	117.2(15)
C88–C87–C86	114.1(14)	C40–C41–C42	120.7(14)
C50–C49–C48	119.5(11)	C66–C67–C68	119.8(17)

C22–C21–C20	118.8(17)	Bonds to hydrogen atoms were omitted.

Table S10. Torsion angles for **3**.

Atom–Atom–Atom–Atom	Torsion Angle [°]
Bi2–N6–C92–P4	0.6(6)
Bi2–N6–C58–C59	2.0(8)
Bi2–N6–C58–C57	−176.5(8)
Bi2–N5–C59–C54	166.4(8)
Bi2–N5–C59–C58	−23.2(8)
Bi2–N5–C47–C48	21.3(7)
Bi2–N5–C47–C52	−165.0(7)
Bi2–N4–C48–C47	−5.7(8)
Bi2–N4–C48–C49	174.6(7)
Bi2–N4–C64–P3	54.3(5)
Bi1–N3–C12–C11	−175.8(7)
Bi1–N3–C12–C13	4.6(7)
Bi1–N3–C31–P2	−53.0(5)
Bi1–N1–C2–C1	−1.6(8)
Bi1–N1–C2–C3	178.2(7)
Bi1–N1–C18–P1	5.9(6)
Bi1–N2–C1–C2	21.3(7)
Bi1–N2–C1–C6	−166.2(8)
Bi1–N2–C13–C12	−19.0(7)
Bi1–N2–C13–C8	164.3(7)
P4–C92–N6–C58	165.9(7)
P4–C83–C88–C87	177.2(10)
P4–C83–C84–C85	−177.0(13)
P4–C77–C82–C81	178.0(11)
P4–C77–C78–C79	−177.9(8)
P2–C31–N3–C12	112.4(8)
P2–C38–C43–C42	172.7(10)
P2–C38–C39–C40	−174.9(14)
P2–C32–C33–C34	172.8(10)
P2–C32–C37–C36	−173.3(10)
P3–C64–N4–C48	−115.2(7)
P3–C71–C72–C73	−175.3(10)
P3–C71–C76–C75	175.8(12)
P3–C65–C70–C69	−176.6(10)
P3–C65–C66–C67	176.7(12)
P1–C25–C30–C29	178.7(12)
P1–C25–C26–C27	−176.2(11)
P1–C19–C20–C21	175.0(11)
P1–C19–C24–C23	−176.0(10)
P1–C18–N1–C2	−159.9(7)
N6–C58–C59–N5	13.5(11)
N6–C58–C59–C54	−176.5(10)
N6–C58–C57–C56	175.9(13)
N3–C12–C11–C10	172.6(11)
N3–C12–C13–C8	−174.2(9)
N3–C12–C13–N2	9.0(10)
N1–C2–C1–C6	175.5(10)
N1–C2–C1–N2	−12.4(11)
N1–C2–C3–C4	−176.9(13)
N5–C59–C54–C53	−12.4(12)
N5–C59–C54–C55	168.9(11)
N5–C59–C58–C57	−167.8(10)
N5–C47–C48–N4	−10.0(11)
N5–C47–C48–C49	169.7(9)
N5–C47–C52–C53	9.8(12)
N5–C47–C52–C51	−173.2(11)
N4–C48–C47–C52	176.5(9)
N4–C48–C49–C50	−175.2(11)
C25–C30–C29–C28	−4.1(17)

C25–C26–C27–C28	−1.0(17)
C1–C2–C3–C4	2.8(13)
C1–C6–C7–C8	−26.6(11)
C1–C6–C7–C16	−150.2(11)
C1–C6–C7–C15	88.6(11)
C1–C6–C5–C4	0.2(13)
C1–N2–C13–C12	170.1(10)
C1–N2–C13–C8	−6.5(12)
C48–C47–C52–C53	−177.0(11)
C48–C47–C52–C51	−0.0(12)
C48–C49–C50–C51	−2.8(12)
C48–C49–C50–C60	176.2(10)
C12–C11–C10–C9	5.8(13)
C12–C11–C10–C17	−173.4(10)
C12–C13–C8–C7	174.2(9)
C12–C13–C8–C9	−2.3(12)
C54–C59–C58–C57	2.3(12)
C54–C53–C52–C47	−22.5(11)
C54–C53–C52–C51	160.7(10)
C54–C55–C56–C57	0.2(15)
C54–C55–C56–C63	179.6(13)
C59–C58–C57–C56	−2.6(13)
C58–C57–C56–C55	1.5(14)
C58–C57–C56–C63	−177.9(12)
C11–C10–C9–C8	−1.7(14)
C83–C88–C87–C86	1.4(15)
C83–C84–C85–C86	−1.7(16)
C19–C20–C21–C22	3.3(16)
C19–C24–C23–C22	−1.7(16)
C8–C7–C6–C5	156.1(9)
C8–C9–C10–C17	177.5(12)
C47–C52–C53–C62	−152.5(11)
C47–C52–C53–C61	90.5(11)
C47–C52–C51–C50	2.7(12)
C33–C34–C35–C36	1.5(17)
C33–C32–C37–C36	0.7(13)
C71–C72–C73–C74	0.5(15)
C71–C76–C75–C74	−2.0(17)
C52–C51–C50–C49	−1.3(14)
C52–C51–C50–C60	179.6(12)
C43–C42–C41–C40	0.4(18)
C43–C38–C39–C40	−1.1(15)
C7–C6–C5–C4	177.7(12)
C79–C78–C77–C82	−0.0(13)
C79–C80–C81–C82	3.0(16)
C2–C3–C4–C5	−0.2(14)
C2–C3–C4–C14	−179.9(13)
C72–C73–C74–C75	−2.2(16)
C65–C70–C69–C68	0.3(15)
C65–C66–C67–C68	−0.5(17)
C6–C5–C4–C3	−1.5(15)
C6–C5–C4–C14	178.2(14)
C70–C69–C68–C67	−1.8(18)
C76–C75–C74–C73	3.0(19)
C20–C21–C22–C23	−3.5(16)
C37–C36–C35–C34	−2.0(15)
C39–C40–C41–C42	−3(2)
C27–C28–C29–C30	5(2)
C69–C68–C67–C66	2(2)
C24–C23–C22–C21	2.8(16)

Bonds to hydrogen atoms were omitted.

Crystallographic Details of 4

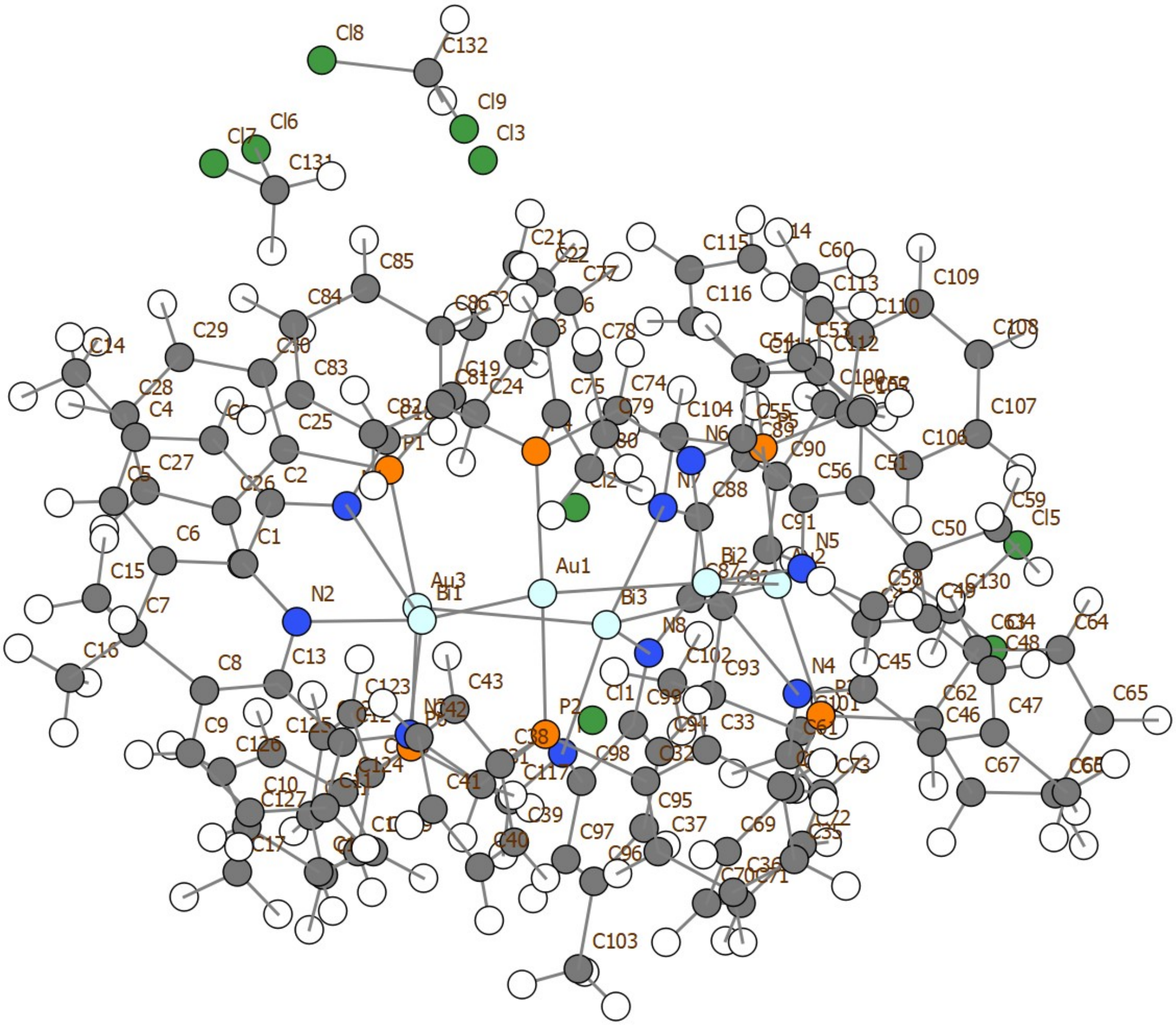

A clear dark blue, block-shaped crystal of mo_ja23_ja_iii_au20_1_0m_a (**4**) was mounted on a MiTeGen micromount with perfluoroether oil. Data were collected from a shock-cooled single crystal at 100.00 K on a Bruker D8 VENTURE dual wavelength Mo/Cu three-circle diffractometer with a microfocus sealed X-ray tube using a mirror optics as monochromator and a Bruker PHOTON III detector. The diffractometer was equipped with an Oxford Cryostream 800 low temperature device and used Mo K_α radiation (λ = 0.71073 Å). All data were integrated with SAINT and a multi-scan absorption correction using SADABS was applied.[S2,S3] The structure was solved by iterative methods using olex2.solve and refined by full-matrix least-squares methods against F^2 by olex2.refine using Olex2.[S4] All non-hydrogen atoms were refined with anisotropic displacement parameters. All hydrogen atoms were refined isotropic on calculated positions using a riding model with their U_{iso} values constrained to 1.5 times the U_{eq} of their pivot atoms for terminal sp^3 carbon atoms and 1.2 times for all other carbon atoms. Disordered moieties were refined using bond lengths restraints and displacement parameter restraints. Crystallographic data for the structures reported here have not been deposited with the Cambridge Crystallographic Data Centre.[S5] Despite numerous attempts, no data set with sufficient resolution and quality could be obtained. Nevertheless, the overall connectivity of the cluster can be demonstrated and is fully in line with the other spectroscopic results. This report was generated using FinalCif.[S6]

Table S11. Crystal data and structure refinement for mo_ja23_ja_iii_au20_1_0m_a

CCDC number	Not deposited
Empirical formula	$C_{281}H_{300}Au_6Bi_6Cl_{12}N_{18}O_5P_{12}$
Formula weight	7242.429
Temperature [K]	100.00
Crystal system	monoclinic
Space group (number)	$P2_1/c$ (14)
a [Å]	17.351(3)
b [Å]	30.435(5)
c [Å]	28.042(5)
α [°]	90
β [°]	102.440(5)
γ [°]	90
Volume [$Å^3$]	14460(4)
Z	2
ρ_{calc} [gcm^{-3}]	1.663
μ [mm^{-1}]	6.898
F(000)	6969.014
Crystal size [mm^3]	0.365×0.144×0.144
Crystal colour	clear dark blue
Crystal shape	block
Radiation	Mo K_α (λ=0.71073 Å)
2θ range [°]	3.68 to 42.10 (0.99 Å)
Index ranges	−17 ≤ h ≤ 17<br>−30 ≤ k ≤ 28<br>−28 ≤ l ≤ 28
Reflections collected	152903
Independent reflections	15340<br>R_{int} = 0.1302<br>R_{sigma} = 0.0596
Completeness to θ = 21.0485°	98.1 %
Data / Restraints / Parameters	15340/8/949
Absorption correction T_{min}/T_{max} (method)	0.2326/0.7446<br>(multi-scan)
Goodness-of-fit on F^2	1.1942
Final R indexes [$I \geq 2\sigma(I)$]	R_1 = 0.0799<br>wR_2 = 0.1764
Final R indexes [all data]	R_1 = 0.1129<br>wR_2 = 0.2152
Largest peak/hole [$eÅ^{-3}$]	3.23/−2.33

Table S12. Bond lengths and angles for mo_ja23_ja_iii_au20_1_0m_a

Atom–Atom	Length [Å]
Bi1–Au1	3.2565(17)
Bi1–Au3	3.5389(18)
Bi1–N1	2.33(2)
Bi1–N2	2.26(2)
Bi1–N3	2.33(2)
Bi2–Au1	3.2950(17)
Bi2–Au2	3.3386(17)
Bi2–N4	2.37(2)
Bi2–N5	2.21(2)
Bi2–N6	2.28(2)
Bi3–Au2	3.2884(16)
Bi3–Au3	3.1715(16)
Bi3–N7	2.35(2)
Bi3–N8	2.27(2)
Bi3–N9	2.31(2)
Au1–P2	2.338(7)
Au1–P4	2.344(8)
Au2–P3	2.315(7)
Au2–P5	2.330(7)
Au3–P1	2.327(7)
Au3–P6	2.328(7)
P1–C18	1.92(3)
P1–C19	1.81(3)
P1–C25	1.84(3)
P2–C31	1.83(2)
P2–C32	1.81(3)
P2–C38	1.89(4)
P3–C61	1.88(3)
P3–C62	1.79(3)
P3–C68	1.77(3)
P4–C74	1.86(3)
P4–C75	1.78(3)
P4–C81	1.81(3)
P5–C104	1.83(3)
P5–C105	1.81(3)
P5–C111	1.78(3)
P6–C117	1.84(3)
P6–C118	1.84(3)
P6–C124	1.87(4)
N1–C2	1.31(3)
N1–C18	1.48(4)
N2–C1	1.35(3)
N2–C13	1.39(3)
N3–C12	1.34(3)
N3–C31	1.47(3)
N4–C45	1.32(3)
N4–C61	1.40(3)
N5–C44	1.38(3)
N5–C56	1.40(3)
N6–C55	1.35(3)
N6–C74	1.48(3)
N7–C88	1.34(3)
N7–C104	1.42(3)
N8–C87	1.35(3)
N8–C99	1.34(3)
N9–C98	1.36(3)
N9–C117	1.38(3)
C1–C2	1.38(4)
C1–C6	1.45(4)
C2–C3	1.45(4)
C3–C4	1.42(4)
C4–C5	1.38(4)
C4–C14	1.48(4)
C5–C6	1.33(4)
C6–C7	1.61(4)
C7–C8	1.56(4)
C7–C15	1.47(3)
C7–C16	1.49(4)
C8–C9	1.38(4)
C8–C13	1.40(4)
C9–C10	1.45(4)
C10–C11	1.41(4)
C10–C17	1.41(4)
C11–C12	1.44(4)
C12–C13	1.46(4)
C19–C20	1.34(4)
C19–C24	1.41(4)
C20–C21	1.48(4)
C21–C22	1.31(4)
C22–C23	1.37(4)
C23–C24	1.40(4)
C25–C26	1.39(4)
C25–C30	1.38(4)
C26–C27	1.40(5)
C27–C28	1.36(5)
C28–C29	1.31(5)
C29–C30	1.39(4)
C32–C33	1.38(4)
C32–C37	1.36(4)
C33–C34	1.38(4)
C34–C35	1.45(4)
C35–C36	1.36(4)
C36–C37	1.42(4)
C38–C39	1.38(5)
C38–C43	1.35(5)
C39–C40	1.35(5)
C40–C41	1.42(5)
C41–C42	1.28(5)
C42–C43	1.42(5)
C44–C45	1.45(4)
C44–C49	1.40(4)
C45–C46	1.44(4)
C46–C47	1.32(4)
C47–C48	1.44(4)
C47–C57	1.52(4)
C48–C49	1.36(4)
C49–C50	1.52(4)
C50–C51	1.47(4)
C50–C58	1.63(3)
C50–C59	1.54(4)
C51–C52	1.52(4)
C51–C56	1.39(4)
C52–C53	1.40(4)
C53–C54	1.31(4)
C53–C60	1.61(4)
C54–C55	1.41(4)
C55–C56	1.43(4)
C62–C63	1.41(4)
C62–C67	1.37(4)
C63–C64	1.36(4)
C64–C65	1.32(4)
C65–C66	1.42(4)
C66–C67	1.41(4)

C68–C69	1.48(4)
C68–C73	1.39(4)
C69–C70	1.35(4)
C70–C71	1.38(5)
C71–C72	1.41(5)
C72–C73	1.29(4)
C75–C76	1.37(4)
C75–C80	1.48(4)
C76–C77	1.45(4)
C77–C78	1.45(5)
C78–C79	1.30(4)
C79–C80	1.47(4)
C81–C82	1.31(4)
C81–C86	1.40(4)
C82–C83	1.40(4)
C83–C84	1.30(4)
C84–C85	1.44(4)
C85–C86	1.44(4)
C87–C88	1.52(4)
C87–C92	1.35(4)
C88–C89	1.46(4)
C89–C90	1.32(4)
C90–C91	1.35(4)
C90–C100	1.59(4)
C91–C92	1.33(4)
C92–C93	1.61(4)
C93–C94	1.50(4)
C93–C101	1.60(4)
C93–C102	1.63(4)
C94–C95	1.38(4)
C94–C99	1.37(4)
C95–C96	1.39(4)
C96–C97	1.32(4)
C96–C103	1.54(4)
C97–C98	1.47(4)
C98–C99	1.50(4)
C105–C106	1.33(5)
C105–C110	1.37(5)
C106–C107	1.45(4)
C107–C108	1.34(5)
C108–C109	1.35(5)
C109–C110	1.29(4)
C111–C112	1.42(4)
C111–C116	1.36(4)
C112–C113	1.44(4)
C113–C114	1.38(4)
C114–C115	1.41(4)
C115–C116	1.32(4)
C118–C119	1.35(4)
C118–C123	1.38(4)
C119–C120	1.38(4)
C120–C121	1.38(4)
C121–C122	1.37(4)
C122–C123	1.33(4)
C124–C125	1.34(5)
C124–C129	1.35(5)
C125–C126	1.37(5)
C126–C127	1.36(5)
C127–C128	1.40(5)
C128–C129	1.53(5)
C130–Cl4	1.62(6)
C130–Cl5	1.79(5)
C131–Cl6	1.74(6)
C131–Cl7	1.70(6)
C132–Cl8	1.78(5)
C132–Cl9	1.77(6)

**Atom–Atom–Atom**	**Angle [°]**
Au3–Bi1–Au1	141.76(5)
N1–Bi1–Au1	114.6(5)
N1–Bi1–Au3	71.5(5)
N2–Bi1–Au1	103.3(5)
N2–Bi1–Au3	113.3(6)
N2–Bi1–N1	68.3(8)
N3–Bi1–Au1	72.9(5)
N3–Bi1–Au3	129.0(5)
N3–Bi1–N1	139.0(8)
N3–Bi1–N2	70.7(8)
Au2–Bi2–Au1	145.01(5)
N4–Bi2–Au1	120.5(5)
N4–Bi2–Au2	74.5(5)
N5–Bi2–Au1	101.8(6)
N5–Bi2–Au2	113.2(6)
N5–Bi2–N4	69.8(8)
N6–Bi2–Au1	71.9(6)
N6–Bi2–Au2	118.6(6)
N6–Bi2–N4	140.3(7)
N6–Bi2–N5	70.6(8)
Au3–Bi3–Au2	138.45(5)
N7–Bi3–Au2	71.2(5)
N7–Bi3–Au3	112.0(5)
N8–Bi3–Au2	102.8(5)
N8–Bi3–Au3	117.6(5)
N8–Bi3–N7	71.1(8)
N9–Bi3–Au2	125.4(5)
N9–Bi3–Au3	79.3(5)
N9–Bi3–N7	141.4(8)
N9–Bi3–N8	71.1(8)
Bi2–Au1–Bi1	93.58(4)
P2–Au1–Bi1	89.79(19)
P2–Au1–Bi2	96.87(19)
P4–Au1–Bi1	97.8(2)
P4–Au1–Bi2	90.1(2)
P4–Au1–P2	169.4(3)
Bi3–Au2–Bi2	98.51(4)
P3–Au2–Bi2	87.85(19)
P3–Au2–Bi3	98.36(18)
P5–Au2–Bi2	101.99(19)
P5–Au2–Bi3	89.29(18)
P5–Au2–P3	166.6(3)
Bi3–Au3–Bi1	97.28(4)
P1–Au3–Bi1	85.7(2)
P1–Au3–Bi3	108.23(18)
P6–Au3–Bi1	94.1(2)
P6–Au3–Bi3	86.02(19)
P6–Au3–P1	165.7(3)
C18–P1–Au3	112.9(10)
C19–P1–Au3	118.6(9)
C19–P1–C18	105.4(14)
C25–P1–Au3	109.4(10)
C25–P1–C18	103.4(13)
C25–P1–C19	105.9(13)
C31–P2–Au1	109.8(8)
C32–P2–Au1	116.3(9)
C32–P2–C31	109.3(11)
C38–P2–Au1	112.4(11)

C38–P2–C31	103.9(13)	C6–C5–C4	127(3)
C38–P2–C32	104.4(14)	C5–C6–C1	117(3)
C61–P3–Au2	112.3(9)	C7–C6–C1	120(2)
C62–P3–Au2	110.3(10)	C7–C6–C5	123(3)
C62–P3–C61	105.6(14)	C8–C7–C6	111(2)
C68–P3–Au2	116.5(10)	C15–C7–C6	108(2)
C68–P3–C61	106.9(13)	C15–C7–C8	111(2)
C68–P3–C62	104.5(14)	C16–C7–C6	107(2)
C74–P4–Au1	108.5(9)	C16–C7–C8	110(2)
C75–P4–Au1	112.3(11)	C16–C7–C15	110(2)
C75–P4–C74	106.1(14)	C9–C8–C7	118(2)
C81–P4–Au1	117.8(11)	C13–C8–C7	120(3)
C81–P4–C74	108.7(15)	C13–C8–C9	122(3)
C81–P4–C75	102.7(15)	C10–C9–C8	122(3)
C104–P5–Au2	109.5(9)	C11–C10–C9	117(3)
C105–P5–Au2	111.0(13)	C17–C10–C9	121(3)
C105–P5–C104	106.3(15)	C17–C10–C11	122(3)
C111–P5–Au2	118.6(11)	C12–C11–C10	122(3)
C111–P5–C104	107.5(14)	C11–C12–N3	127(3)
C111–P5–C105	103.1(15)	C13–C12–N3	115(3)
C117–P6–Au3	115.8(9)	C13–C12–C11	118(3)
C118–P6–Au3	111.1(10)	C8–C13–N2	124(3)
C118–P6–C117	101.1(13)	C12–C13–N2	117(2)
C124–P6–Au3	109.2(13)	C12–C13–C8	119(3)
C124–P6–C117	110.5(17)	N1–C18–P1	108.6(19)
C124–P6–C118	108.7(15)	C20–C19–P1	122(2)
C2–N1–Bi1	118.5(19)	C24–C19–P1	116(2)
C18–N1–Bi1	119.7(18)	C24–C19–C20	121(3)
C18–N1–C2	122(2)	C21–C20–C19	120(3)
C1–N2–Bi1	119.4(18)	C22–C21–C20	118(3)
C13–N2–Bi1	118.4(17)	C23–C22–C21	123(3)
C13–N2–C1	122(2)	C24–C23–C22	120(3)
C12–N3–Bi1	118.7(18)	C23–C24–C19	118(3)
C31–N3–Bi1	120.5(16)	C26–C25–P1	117(2)
C31–N3–C12	119(2)	C30–C25–P1	121(2)
C45–N4–Bi2	116.7(17)	C30–C25–C26	121(3)
C61–N4–Bi2	121.4(17)	C27–C26–C25	118(3)
C61–N4–C45	121(2)	C28–C27–C26	120(3)
C44–N5–Bi2	121.6(17)	C29–C28–C27	121(3)
C56–N5–Bi2	118.8(18)	C30–C29–C28	122(3)
C56–N5–C44	119(2)	C29–C30–C25	117(3)
C55–N6–Bi2	120.0(18)	N3–C31–P2	110.2(16)
C74–N6–Bi2	122.3(16)	C33–C32–P2	120(2)
C74–N6–C55	117(2)	C37–C32–P2	121(2)
C88–N7–Bi3	115.5(18)	C37–C32–C33	118(3)
C104–N7–Bi3	120.5(17)	C34–C33–C32	125(3)
C104–N7–C88	122(2)	C35–C34–C33	115(3)
C87–N8–Bi3	121.5(18)	C36–C35–C34	121(3)
C99–N8–Bi3	120.1(18)	C37–C36–C35	119(3)
C99–N8–C87	118(2)	C36–C37–C32	121(3)
C98–N9–Bi3	117.9(18)	C39–C38–P2	116(3)
C117–N9–Bi3	125.2(17)	C43–C38–P2	118(3)
C117–N9–C98	116(2)	C43–C38–C39	125(4)
C2–C1–N2	117(3)	C40–C39–C38	118(4)
C6–C1–N2	122(3)	C41–C40–C39	116(4)
C6–C1–C2	121(3)	C42–C41–C40	125(4)
C1–C2–N1	116(3)	C43–C42–C41	119(4)
C3–C2–N1	125(3)	C42–C43–C38	115(4)
C3–C2–C1	119(3)	C45–C44–N5	114(2)
C4–C3–C2	120(3)	C49–C44–N5	124(3)
C5–C4–C3	117(3)	C49–C44–C45	121(3)
C14–C4–C3	122(3)	C44–C45–N4	117(3)
C14–C4–C5	121(3)	C46–C45–N4	129(3)

C46–C45–C44	114(3)
C47–C46–C45	125(3)
C48–C47–C46	118(3)
C57–C47–C46	122(3)
C57–C47–C48	120(3)
C49–C48–C47	121(3)
C48–C49–C44	120(3)
C50–C49–C44	118(3)
C50–C49–C48	123(3)
C51–C50–C49	112(2)
C58–C50–C49	103(2)
C58–C50–C51	106(2)
C59–C50–C49	114(2)
C59–C50–C51	116(2)
C59–C50–C58	104(2)
C52–C51–C50	118(2)
C56–C51–C50	124(3)
C56–C51–C52	118(3)
C53–C52–C51	114(3)
C54–C53–C52	127(3)
C60–C53–C52	111(2)
C60–C53–C54	122(3)
C55–C54–C53	122(3)
C54–C55–N6	131(3)
C56–C55–N6	114(2)
C56–C55–C54	116(3)
C51–C56–N5	119(3)
C55–C56–N5	117(2)
C55–C56–C51	124(3)
N4–C61–P3	110.3(18)
C63–C62–P3	122(2)
C67–C62–P3	123(2)
C67–C62–C63	115(3)
C64–C63–C62	125(3)
C65–C64–C63	120(3)
C66–C65–C64	120(3)
C67–C66–C65	119(3)
C66–C67–C62	122(3)
C69–C68–P3	123(2)
C73–C68–P3	123(2)
C73–C68–C69	114(2)
C70–C69–C68	118(3)
C71–C70–C69	124(3)
C72–C71–C70	117(3)
C73–C72–C71	120(4)
C72–C73–C68	126(3)
N6–C74–P4	107.5(18)
C76–C75–P4	120(2)
C80–C75–P4	119(2)
C80–C75–C76	120(3)
C77–C76–C75	119(3)
C78–C77–C76	118(3)
C79–C78–C77	122(3)
C80–C79–C78	120(3)
C79–C80–C75	118(3)
C82–C81–P4	120(3)
C86–C81–P4	120(3)
C86–C81–C82	120(3)
C83–C82–C81	121(3)
C84–C83–C82	123(4)
C85–C84–C83	119(3)
C86–C85–C84	116(3)
C85–C86–C81	120(3)

C88–C87–N8	112(2)
C92–C87–N8	129(3)
C92–C87–C88	119(3)
C87–C88–N7	119(2)
C89–C88–N7	128(3)
C89–C88–C87	113(2)
C90–C89–C88	121(3)
C91–C90–C89	123(3)
C100–C90–C89	114(3)
C100–C90–C91	123(3)
C92–C91–C90	121(3)
C91–C92–C87	123(3)
C93–C92–C87	114(3)
C93–C92–C91	122(3)
C94–C93–C92	114(2)
C101–C93–C92	108(2)
C101–C93–C94	113(3)
C102–C93–C92	106(2)
C102–C93–C94	110(2)
C102–C93–C101	105(2)
C95–C94–C93	120(3)
C99–C94–C93	119(3)
C99–C94–C95	121(3)
C96–C95–C94	122(3)
C97–C96–C95	119(3)
C103–C96–C95	123(3)
C103–C96–C97	118(3)
C98–C97–C96	125(3)
C97–C98–N9	131(3)
C99–C98–N9	115(2)
C99–C98–C97	114(2)
C94–C99–N8	125(3)
C98–C99–N8	116(2)
C98–C99–C94	119(3)
N7–C104–P5	110.8(19)
C106–C105–P5	122(3)
C110–C105–P5	122(3)
C110–C105–C106	117(3)
C107–C106–C105	119(3)
C108–C107–C106	119(4)
C109–C108–C107	120(4)
C110–C109–C108	119(4)
C109–C110–C105	126(4)
C112–C111–P5	116(2)
C116–C111–P5	126(3)
C116–C111–C112	118(3)
C113–C112–C111	119(3)
C114–C113–C112	120(3)
C115–C114–C113	117(3)
C116–C115–C114	123(3)
C115–C116–C111	123(3)
N9–C117–P6	107.5(19)
C119–C118–P6	118(2)
C123–C118–P6	122(2)
C123–C118–C119	120(3)
C120–C119–C118	121(3)
C121–C120–C119	118(3)
C122–C121–C120	120(3)
C123–C122–C121	121(3)
C122–C123–C118	120(3)
C125–C124–P6	112(3)
C129–C124–P6	118(3)
C129–C124–C125	129(4)

C126–C125–C124	118(4)
C127–C126–C125	120(4)
C128–C127–C126	124(4)
C129–C128–C127	116(4)
C128–C129–C124	113(4)
Cl5–C130–Cl4	114(3)
Cl7–C131–Cl6	118(3)
Cl9–C132–Cl8	111(3)

Bonds to hydrogen atoms were omitted.

Table S13. Torsion angles for mo_ja23_ja_iii_au20_1_0m_a

**Atom–Atom–Atom–Atom**	**Torsion Angle [°]**
Bi1–N1–C2–C1	5(2)
Bi1–N1–C2–C3	−172.8(18)
Bi1–N1–C18–P1	80.8(15)
Bi1–N2–C1–C2	−4(2)
Bi1–N2–C1–C6	−179.7(19)
Bi1–N2–C13–C8	179.2(19)
Bi1–N2–C13–C12	1.5(19)
Bi1–N3–C12–C11	−178.8(18)
Bi1–N3–C12–C13	8.9(19)
Bi1–N3–C31–P2	75.5(14)
Bi2–N4–C45–C44	8(2)
Bi2–N4–C45–C46	−173.1(18)
Bi2–N4–C61–P3	73.2(15)
Bi2–N5–C44–C45	0(2)
Bi2–N5–C44–C49	−178(2)
Bi2–N5–C56–C51	177(2)
Bi2–N5–C56–C55	−4(2)
Bi2–N6–C55–C54	179.3(19)
Bi2–N6–C55–C56	−2(2)
Bi2–N6–C74–P4	80.6(15)
Bi3–N7–C88–C87	8(2)
Bi3–N7–C88–C89	−173.1(19)
Bi3–N7–C104–P5	76.9(15)
Bi3–N8–C87–C88	−5(2)
Bi3–N8–C87–C92	177(2)
Bi3–N8–C99–C94	−176(2)
Bi3–N8–C99–C98	5(2)
Bi3–N9–C98–C97	177(2)
Bi3–N9–C98–C99	3(2)
Bi3–N9–C117–P6	64.4(16)
P1–C18–N1–C2	−101(2)
P1–C19–C20–C21	−174(2)
P1–C19–C24–C23	176(2)
P1–C25–C26–C27	168(3)
P1–C25–C30–C29	−169(3)
P2–C31–N3–C12	−89(2)
P2–C32–C33–C34	174(2)
P2–C32–C37–C36	−174(2)
P2–C38–C39–C40	−178(3)
P2–C38–C43–C42	176(3)
P3–C61–N4–C45	−98(2)
P3–C62–C63–C64	−178(3)
P3–C62–C67–C66	177(2)
P3–C68–C69–C70	179(3)
P3–C68–C73–C72	−179(3)
P4–C74–N6–C55	−90(2)
P4–C75–C76–C77	−174(3)
P4–C75–C80–C79	180(3)
P4–C81–C82–C83	174(2)
P4–C81–C86–C85	−174(2)
P5–C104–N7–C88	−88(2)
P5–C105–C106–C107	−178(3)
P5–C105–C110–C109	180(3)
P5–C111–C112–C113	178(2)
P5–C111–C116–C115	177(3)
P6–C117–N9–C98	−107(2)
P6–C118–C119–C120	177(2)
P6–C118–C123–C122	179(2)
P6–C124–C125–C126	−170(3)
P6–C124–C129–C128	168(3)
N1–C2–C1–N2	−1(3)
N1–C2–C1–C6	175(2)
N1–C2–C3–C4	−175(3)
N2–C1–C2–C3	177(2)
N2–C1–C6–C5	−178(3)
N2–C1–C6–C7	−6(3)
N2–C13–C8–C7	7(3)
N2–C13–C8–C9	−178(3)
N2–C13–C12–N3	−7(3)
N2–C13–C12–C11	−180(2)
N3–C12–C11–C10	−176(3)
N3–C12–C13–C8	175(2)
N4–C45–C44–N5	−6(3)
N4–C45–C44–C49	172(3)
N4–C45–C46–C47	−179(3)
N5–C44–C45–C46	175(2)
N5–C44–C49–C48	−173(3)
N5–C44–C49–C50	8(3)
N5–C56–C51–C50	−7(3)
N5–C56–C51–C52	177(2)
N5–C56–C55–N6	4(3)
N5–C56–C55–C54	−178(3)
N6–C55–C54–C53	179(3)
N6–C55–C56–C51	−177(2)
N7–C88–C87–N8	−2(3)
N7–C88–C87–C92	177(3)
N7–C88–C89–C90	−179(3)
N8–C87–C88–C89	178(3)
N8–C87–C92–C91	−175(3)
N8–C87–C92–C93	−3(4)
N8–C99–C94–C93	1(4)
N8–C99–C94–C95	178(3)
N8–C99–C98–N9	−5(3)
N8–C99–C98–C97	−180(3)
N9–C98–C97–C96	−174(3)
N9–C98–C99–C94	176(3)
C1–C2–C3–C4	7(3)
C1–C6–C5–C4	−7(3)
C1–C6–C7–C8	11(3)
C1–C6–C7–C15	132(3)
C1–C6–C7–C16	−109(3)
C2–C3–C4–C5	−7(3)
C2–C3–C4–C14	176(3)
C3–C4–C5–C6	7(3)
C4–C5–C6–C7	−178(3)
C5–C6–C7–C8	−178(3)
C5–C6–C7–C15	−57(3)
C5–C6–C7–C16	62(3)
C6–C7–C8–C9	174(2)
C6–C7–C8–C13	−11(2)
C7–C8–C9–C10	174(2)
C7–C8–C13–C12	−175(2)
C8–C9–C10–C11	−1(3)

C8–C9–C10–C17	176(3)
C8–C13–C12–C11	2(3)
C9–C10–C11–C12	4(3)
C10–C11–C12–C13	−4(3)
C19–C20–C21–C22	−3(3)
C19–C24–C23–C22	0(3)
C20–C21–C22–C23	4(3)
C21–C22–C23–C24	−3(4)
C25–C26–C27–C28	5(4)
C25–C30–C29–C28	−3(4)
C26–C27–C28–C29	−9(5)
C27–C28–C29–C30	8(5)
C32–C33–C34–C35	2(3)
C32–C37–C36–C35	−1(4)
C33–C34–C35–C36	−3(3)
C34–C35–C36–C37	2(3)
C38–C39–C40–C41	1(4)
C38–C43–C42–C41	3(4)
C39–C40–C41–C42	1(4)
C40–C41–C42–C43	−3(4)
C44–C45–C46–C47	−1(3)
C44–C49–C48–C47	−5(3)
C44–C49–C50–C51	−21(3)
C44–C49–C50–C58	93(3)
C44–C49–C50–C59	−155(3)
C45–C46–C47–C48	4(3)
C45–C46–C47–C57	−179(3)
C46–C47–C48–C49	−2(3)
C47–C48–C49–C50	174(2)
C48–C49–C50–C51	160(3)
C48–C49–C50–C58	−86(3)
C48–C49–C50–C59	26(3)
C49–C50–C51–C52	−163(2)
C49–C50–C51–C56	20(3)
C50–C51–C52–C53	−176(3)
C50–C51–C56–C55	174(3)
C51–C52–C53–C54	3(3)
C51–C52–C53–C60	179(2)
C51–C56–C55–C54	2(3)
C52–C53–C54–C55	−3(4)
C53–C54–C55–C56	1(3)
C62–C63–C64–C65	3(4)
C62–C67–C66–C65	−1(3)
C63–C64–C65–C66	−2(4)
C64–C65–C66–C67	1(4)
C68–C69–C70–C71	2(4)
C68–C73–C72–C71	−2(4)
C69–C70–C71–C72	0(4)
C70–C71–C72–C73	0(4)
C75–C76–C77–C78	−12(4)
C75–C80–C79–C78	2(4)
C76–C77–C78–C79	19(5)
C77–C78–C79–C80	−14(5)
C81–C82–C83–C84	−2(4)
C81–C86–C85–C84	2(4)
C82–C83–C84–C85	5(4)
C83–C84–C85–C86	−5(4)
C87–C88–C89–C90	0(3)
C87–C92–C91–C90	−8(4)
C87–C92–C93–C94	7(3)
C87–C92–C93–C101	−120(3)
C87–C92–C93–C102	129(3)
C88–C89–C90–C91	−1(4)
C88–C89–C90–C100	180(3)
C89–C90–C91–C92	5(4)
C90–C91–C92–C93	−179(3)
C91–C92–C93–C94	179(3)
C91–C92–C93–C101	52(3)
C91–C92–C93–C102	−59(3)
C92–C93–C94–C95	177(3)
C92–C93–C94–C99	−6(3)
C93–C94–C95–C96	−179(3)
C93–C94–C99–C98	−179(3)
C94–C95–C96–C97	−4(4)
C94–C95–C96–C103	−178(3)
C94–C99–C98–C97	1(3)
C95–C96–C97–C98	2(4)
C96–C97–C98–C99	0(4)
C105–C106–C107–C108	0(4)
C105–C110–C109–C108	−2(4)
C106–C107–C108–C109	3(4)
C107–C108–C109–C110	−2(4)
C111–C112–C113–C114	4(3)
C111–C116–C115–C114	5(4)
C112–C113–C114–C115	0(3)
C113–C114–C115–C116	−4(4)
C118–C119–C120–C121	7(3)
C118–C123–C122–C121	1(3)
C119–C120–C121–C122	−7(3)
C120–C121–C122–C123	3(4)
C124–C125–C126–C127	1(4)
C124–C129–C128–C127	1(4)
C125–C126–C127–C128	−3(4)
C126–C127–C128–C129	2(4)

Bonds to hydrogen atoms were omitted.

X-ray Absorption Near Edge Structure (XANES) spectroscopy

XANES measurements were performed in transmission at the Bi L_1-edge (16388 eV) and Bi L_3-edge (13419 eV) at the BAMline, located at BESSY II (Berlin, Germany).[S7] The incident energy was tuned by a double crystal monochromator in a Si(111) arrangement ($\Delta E/E = 2x10^{-4}$). Two ionization chambers were used to measure the I0 signal (before the sample) and the I1 signal (after the sample). For I_0 and I_1 a 5 cm and 15 cm long chamber was used, respectively. The chambers were filled with Ar. The beam size was 4 mm (horizontal) x 2 mm (vertical). The measurement protocol was the following: 10 eV steps until 20 eV before the edge, followed by 0.5 eV steps until 20 eV above the edge and 2 eV steps until 200 eV above the edge. From then on equidistant k-steps were taken (every 2.4 Å) until 14 Å.
The data evaluation and treatment were performed by using the ATHENA program from the DEMETER package.[S8] The Bi-foil was used to calibrate the energy delivered by the Si(111) monochromator. All spectra were normalized to the pre-edge (linear funtion) and post-edge (a degree 3 polynomial function).

Bismuth has a ground state electron configuration of [Xe] $4f^{14}5d^{10}6s^{2}6p^{3}$. For lower oxidation states (until 3+) the 6p orbitals are of interest, and as such absorption transitions between 2s → 6p are relevant, which can be probed by Bi L_1-edge. For higher oxidation states, dipole allows 2p2/3 → 6s or 5d, which can be probed by Bi L_3-edge.[S9] In the case of Bi L_1-edge, the higher the oxidation state the higher the energy is to promote 2s electrons into empty 6p orbitals, which translates into a higher absorption edge energy in the XANES spectra (Figure 2 / Figure S34). Bi L_1 transitions have a sharper maximum than the Bi L_3 transitions. This is because in the former transitions are more localized (only to unoccupied 6p states), while in the latter this is delocalized (to both unoccupied 6s and 5d states). Since these samples show a maximum oxidation of 3+, Bi L_3-edge (Figure S36) was less sensitive to the shift than the Bi L_1-edge (Figure S34). Nevertheless, the Bi L_3-edge XANES spectra reveal that the coordination environment is different between **2** and $BiPh_3$. $BiPh_3$ has a shoulder at lower energy (13412.3 eV), which is stronger that the main absorption edge (at 13418 eV), where the edge of **2** falls. Both the oxidation state and the bond length affect the energy shift. In coordination environments that promote a strong hybridization of the 6s and 5d orbitals, and additional orbitals (p and f), the pre-edge peak becomes noticeable. This is the case of $BiPh_3$, which results in the shoulder at the edge in Figure S36.

Another important conclusion is the inferring about the stability of the **2** sample. As such we probed the Bi L_1-edge after 1 h and the difference spectrum demonstrating the stability of the sample over the course of data acquisition is shown in Figure S35.

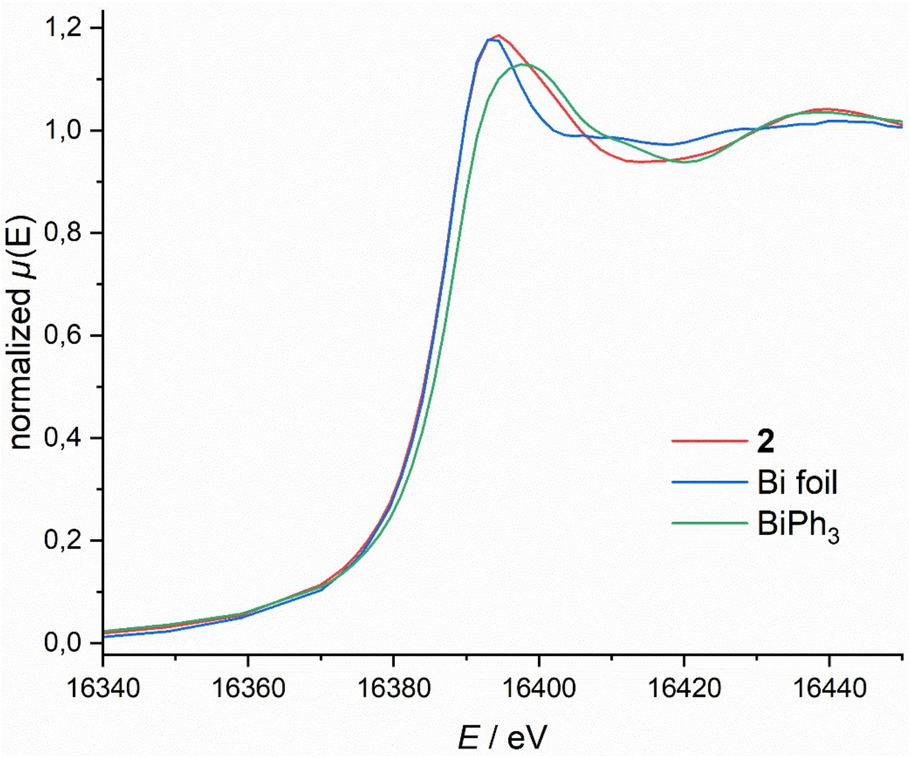

Figure S40. Bi L_1-edge XANES spectrum of **2**, Bi foil and $BiPh_3$.

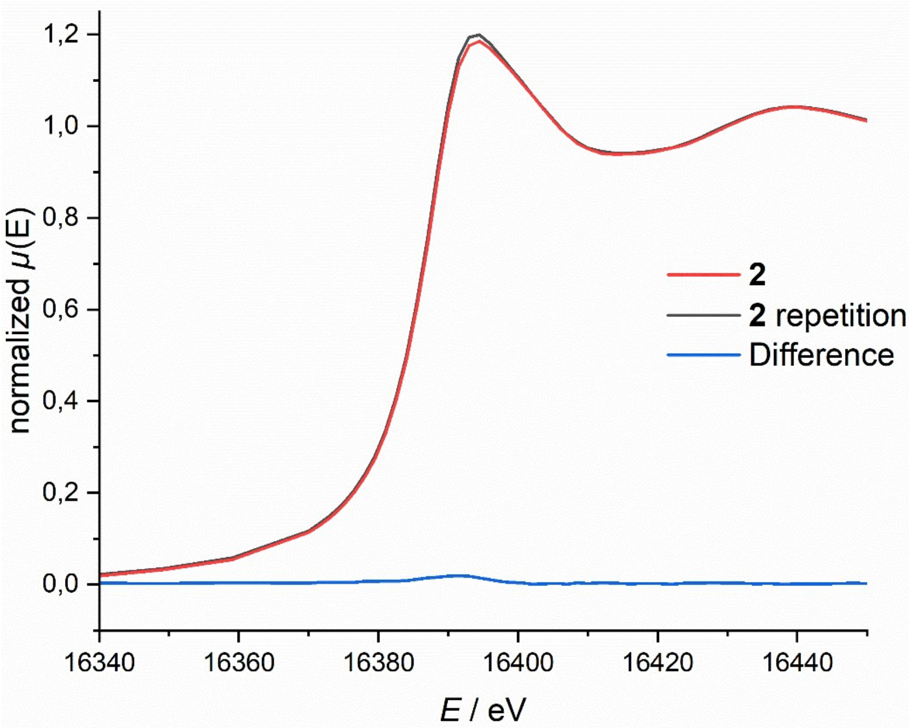

Figure S41. Bi L_1-edge XANES spectrum of **2** after 1 h and the difference spectrum demonstrating the stability of the sample over the course of data acquisition.

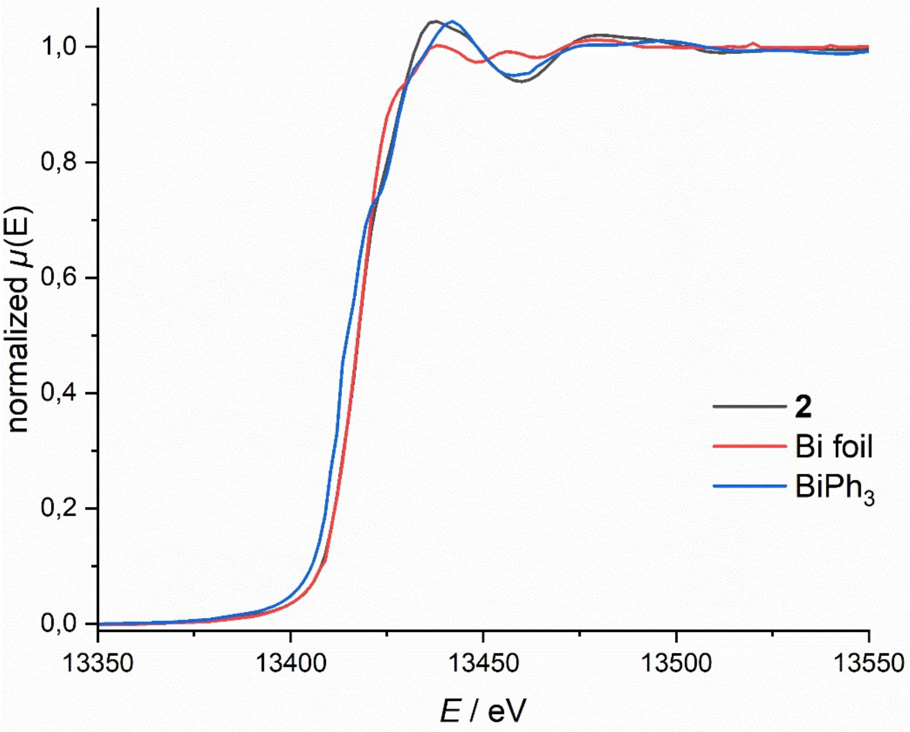

Figure S42. Bi L_3-edge XANES spectrum of **2**, Bi foil and $BiPh_3$.

Computational Details

All calculations were carried out with the ORCA program package.[S10] Density fitting techniques, also called resolution-of-identity approximation (RI)[S11], were used for all BP86, PBE and TPSS calculations, whereas an additional chain-of-spheres approximation for the HF exchange (RIJCOSX)[S12] was used for CAM-B3LYP calculations. Atom-pairwise dispersion corrections were used for all DFT calculations (D3BJ).[S13,S14] All geometries and thermal corrections were obtained with the BP86-D3BJ/def2-SVP (**4**) and PBE-D3BJ/def2-SVP (**2**, **3**) (method on isolated molecules using a medium-tight grid (Grid5) and TIGHTSCF convergence criteria for SCF procedure and the default convergence criteria for geometry optimisations. TDDFT calculations were conducted using CAM-B3LYP/def2-TZVP[S15] with the CPCM[S16] model for dichlormethane. CASSCF calculations were conducted using the def2-SVP basis set. For all calculations using the def2-SVP and def2-TZVP, the respective build-in effective core potentials (ECP) of the ORCA program package were used. EDA-NOCV calculations were conducted using TPSS-D3BJ[S17] together with the DKH-Hamiltonian and the all electron relativistic basis set cc-pVTZ-DK. In addition, the CPCM model for THF was used to avoid the domination of the orbital term by electrostatic and polarization interactions. Plots of the Laplacian and Electron Localisation Function (ELF) of the tungsten complex were performed on the density obtained from the EDA calculations (TPSS-D3BJ/cc-pVTZ-DK) using the program Multiwfn.[S18] The methyl groups on the NNN scaffold were truncated to H to improve computational efficiency. **4** could not be optimized due to its large size and consequently the PPh_2 moieties were truncated to PMe_2, while only the equatiorial Me groups of the acridane ligand were truncated to H to realize similar bond geometries when compared to the molecular structures derived from SCXRD.

The transition state for the rocking motion of the Bi ligand around the metal center in complex **3** was obtained as follows: First, a relaxed surface scan was performed, where the N–Bi–W angle was widened from 100 to 175°. This scan showed a monotonous increase in energy. Thus, a frequency calculation on the 175° geometry was performed and the obtained hessian with one imaginary mode was used as an input for an OPTTS calculation using ORCA.

A transition state was located with one imaginary frequency that corresponds to a rocking mode of the Bi pincer around the $W(CO)_3$ moiety. An activation barrier of 12 kcal/mol was obtained in line with a sharpening of resonances in the ^1H NMR at low temperatures.

Fluoride Ion Affinities were calculated in the gas phase following the protocol established by Greb and co-workers.[60] Calculations were performed on the PW6B95-D3BJ/def2-TZVPP level of theory with the COF_2 reference system.[S13-S15,S19] The obtained FIAs (**2**: 61 kcal/mol; **3**: 56 kcal/mol) are well below the computed value for BH_3 (68 kcal/mol) rendering both complexes as weak Lewis-acids.

XYZ-coordinates

2

77

C	3.627044	17.712055	0.358447
C	2.529381	17.193076	-0.326378
C	4.606912	16.838775	0.915381
H	3.732645	18.798055	0.487658
C	4.408511	15.414047	0.759607
C	3.278454	14.902634	0.069893
C	2.354801	15.799599	-0.476405
H	1.476263	15.409121	-1.013551
H	1.779856	17.881108	-0.746857
C	3.080341	13.404198	0.001471
C	4.329748	12.606256	0.303457
C	5.399060	13.239381	0.988848
C	6.578354	12.498914	1.382345
C	6.638957	11.116040	1.037162
C	5.590019	10.515630	0.342444
H	7.509508	10.519046	1.342045
C	4.441578	11.251275	-0.024167
H	5.653000	9.446676	0.086817
H	3.617260	10.756200	-0.561197
N	5.390720	14.589153	1.259297
N	5.728771	17.218336	1.579053
N	7.533404	13.193941	2.049189
C	8.812266	12.601329	2.342317
C	6.103384	18.604120	1.691472
Bi	7.135524	15.499481	2.351417
H	2.289386	13.116341	0.735628
H	2.670506	13.114821	-0.989156
P	7.981073	18.701854	1.883740
H	5.664784	19.105625	2.586655
H	5.803157	19.206806	0.801149
P	10.086013	13.980785	2.488652
H	9.144310	11.870910	1.566825
H	8.832335	12.054426	3.315241
C	8.370509	23.069160	3.483857
C	8.774671	21.977492	4.266972
C	7.861945	22.855375	2.191055
H	8.454642	24.092669	3.880263
C	7.757904	21.553275	1.682298
H	7.544660	23.710452	1.574908
C	8.160214	20.447928	2.464048
H	7.370441	21.390003	0.664750
C	8.670417	20.672394	3.758645
H	8.990349	19.813218	4.369126
H	9.177034	22.141696	5.277919
C	9.657975	19.583101	-0.219836
C	10.100989	19.647643	-1.549431
C	8.514098	18.828044	0.120204
C	9.415947	18.950262	-2.557740
C	7.842741	18.113502	-0.896397
C	8.290587	18.180829	-2.224313
H	10.195409	20.143222	0.561112
H	10.987194	20.251606	-1.799219
H	6.966163	17.495062	-0.652037
H	9.762235	19.003635	-3.601233
H	7.750839	17.622853	-3.004135
C	10.948471	15.734489	0.511125
C	11.117930	16.233233	-0.787149
C	10.284543	14.507302	0.731165
C	10.601929	15.524438	-1.883375
C	9.770042	13.802119	-0.377197
C	9.925731	14.312229	-1.675207
H	11.320874	16.310989	1.373079
H	11.622715	17.197413	-0.942066
H	9.231163	12.853871	-0.235094
H	10.712408	15.929903	-2.900213
H	9.508757	13.757788	-2.529526
C	12.529662	12.633470	1.795091
C	13.657167	11.856727	2.105038
C	11.600282	12.970211	2.801138
C	13.866176	11.402023	3.416799
C	11.826546	12.522782	4.121596
C	12.945793	11.733857	4.424713
H	12.366179	12.982215	0.764277
H	14.376798	11.600760	1.312034
H	11.119048	12.801567	4.919817
H	14.751056	10.792936	3.656618
H	13.105941	11.384672	5.456225

3

84

C	-2.465600	1.067468	-0.327079
C	-2.295955	-0.170847	-0.937214
C	-1.347293	1.771589	0.216280
H	-3.469145	1.508330	-0.249596
C	-0.050057	1.130949	0.110599
C	0.106538	-0.131765	-0.527373
C	-1.016742	-0.769648	-1.050103
H	-0.907452	-1.745865	-1.545635
H	-3.175476	-0.697237	-1.337562
C	-4.674056	8.783184	0.146407
C	-5.128077	7.501403	-0.209489
C	-3.297034	9.036135	0.232049
H	-5.397360	9.587455	0.350562
C	-2.373706	8.010423	-0.026603
H	-2.933477	10.039313	0.501258
C	-2.819597	6.722130	-0.374128
H	-1.293019	8.205873	0.029038
C	-4.207582	6.477983	-0.473431
H	-4.571785	5.485894	-0.781618
H	-6.207695	7.299970	-0.289983
C	-2.053507	3.498398	-2.805095
C	-2.126417	4.844530	-2.393705
C	-2.347271	3.145490	-4.130722
H	-1.757365	2.712602	-2.097444
C	-2.702546	4.131391	-5.064038
H	-2.288481	2.088491	-4.432920
C	-2.759419	5.476839	-4.664728
H	-2.931149	3.852695	-6.103914
C	-2.474142	5.833873	-3.338805
H	-3.029315	6.258381	-5.391309
H	-2.518123	6.890713	-3.036362

C	4.830470	10.183776	1.194988
C	5.439353	9.024018	1.697507
C	3.761919	10.074972	0.290749
H	5.185672	11.174967	1.514428
C	3.301431	8.812430	-0.107812
H	3.274540	10.980121	-0.101236
C	3.915478	7.642282	0.383764
H	2.445498	8.724407	-0.794692
C	4.987955	7.758128	1.292386
H	5.480603	6.862783	1.700572
H	6.273678	9.103393	2.411470
C	3.905061	5.415324	-2.896498
C	4.432644	5.653137	-1.614441
C	4.758200	5.106698	-3.967780
H	2.815826	5.476261	-3.045294
C	6.144344	5.036457	-3.766182
H	4.332630	4.915439	-4.964481
C	6.680554	5.296166	-2.492420
H	6.812646	4.786925	-4.604409
C	5.830425	5.610755	-1.423924
H	7.769493	5.258437	-2.332635
H	6.264288	5.835432	-0.436860
C	2.303668	1.536028	0.148631
C	2.587704	0.313844	-0.527735
C	3.322893	2.557854	0.330876
C	4.590383	2.331753	-0.294482
C	4.839331	1.145064	-0.969557
H	5.369002	3.104113	-0.244966
C	3.850609	0.131921	-1.082018
H	5.824870	0.989855	-1.433735
H	4.078534	-0.798761	-1.623864
P	-1.613460	5.357828	-0.694870
P	3.274255	6.018721	-0.217734
N	1.035321	1.854868	0.523571
N	-1.386059	2.997939	0.791381
N	2.964302	3.634990	1.055610
C	1.495380	-0.731435	-0.568471
H	1.612039	-1.385628	-1.455991
H	1.616682	-1.403175	0.317154
C	3.795775	4.818407	1.139252
H	4.881686	4.584692	1.114946
H	3.585440	5.346679	2.094885
C	-2.362722	3.988442	0.460770
H	-2.692037	4.555754	1.357954
H	-3.267677	3.577141	-0.032935
Bi	0.650115	3.790200	1.662641
W	0.815079	5.746882	-0.500197
C	1.075911	4.159243	-1.765721
C	0.555055	7.142001	-1.867627
C	0.661683	7.063836	1.042435
O	0.615080	7.776819	1.975098
O	1.237456	3.200631	-2.418774
O	0.379574	7.976614	-2.682873

Relaxed Surface Scan of 3

84

Coordinates from ORCA-job W-scan1 Relaxed Surface Scan Step 1

C	-2.442594	1.101020	-0.420964
C	-2.259826	-0.106214	-1.087286
C	-1.333924	1.781044	0.172240
H	-3.446359	1.541382	-0.344446
C	-0.033100	1.147489	0.063886
C	0.137268	-0.082383	-0.633828
C	-0.975298	-0.695519	-1.208258
H	-0.853971	-1.643633	-1.754233
H	-3.130459	-0.613468	-1.530512
C	-4.608711	8.823686	0.225057
C	-5.092841	7.534465	-0.061423
C	-3.225765	9.065287	0.231304
H	-5.313807	9.642138	0.437133
C	-2.325562	8.020694	-0.037341
H	-2.840404	10.073882	0.445011
C	-2.801771	6.726139	-0.317104
H	-1.242388	8.205049	-0.042653
C	-4.195555	6.492454	-0.336613
H	-4.584237	5.493942	-0.589911
H	-6.176778	7.342248	-0.077687
C	-1.994538	3.519731	-2.797103
C	-2.150826	4.850650	-2.357417
C	-2.289858	3.173079	-4.123801
H	-1.628382	2.744690	-2.111519
C	-2.726159	4.153070	-5.031231
H	-2.166720	2.128437	-4.448149
C	-2.864680	5.484459	-4.603326
H	-2.951754	3.881298	-6.073911
C	-2.580572	5.834168	-3.274171
H	-3.196722	6.260736	-5.309866
H	-2.686684	6.879121	-2.947727
C	4.740249	10.222865	1.077911
C	5.318209	9.089323	1.671944
C	3.725253	10.068294	0.117748
H	5.077274	11.229660	1.368547
C	3.286611	8.786613	-0.244605
H	3.263498	10.953127	-0.346339
C	3.869854	7.643343	0.340993
H	2.474295	8.662644	-0.976641
C	4.888612	7.803439	1.303251
H	5.353866	6.927740	1.780489
H	6.108297	9.204379	2.429933
C	3.927651	5.200360	-2.822617
C	4.429150	5.577604	-1.564270
C	4.807325	4.810596	-3.846046
H	2.841340	5.213329	-2.990750
C	6.192274	4.801773	-3.618496
H	4.404072	4.508195	-4.824364
C	6.701367	5.208435	-2.370726
H	6.881467	4.488414	-4.417731
C	5.824666	5.602395	-1.350520
H	7.788274	5.222763	-2.195748
H	6.230850	5.936925	-0.382894
C	2.318948	1.566206	0.135350
C	2.612382	0.380317	-0.600293
C	3.326302	2.593768	0.349055

C	4.593620	2.410605	-0.290709
C	4.851396	1.259484	-1.022609
H	5.363434	3.187377	-0.206525
C	3.871987	0.240978	-1.175579
H	5.835878	1.136742	-1.499052
H	4.104690	-0.659375	-1.764868
P	-1.624918	5.347754	-0.662480
P	3.249506	5.997116	-0.206917
N	1.045595	1.854009	0.527295
N	-1.386444	2.983546	0.794947
N	2.957130	3.645278	1.107528
C	1.530164	-0.675247	-0.680094
H	1.658500	-1.303154	-1.584495
H	1.648396	-1.368440	0.189070
C	3.766981	4.845985	1.191533
H	4.857090	4.631216	1.193546
H	3.524357	5.385513	2.132514
C	-2.369127	3.978821	0.490065
H	-2.677569	4.536253	1.400719
H	-3.283982	3.572330	0.011200
Bi	0.637929	3.776296	1.679923
W	0.800704	5.713843	-0.493066
C	1.050684	4.103643	-1.726839
C	0.541511	7.088934	-1.882394
C	0.663715	7.035896	1.048403
O	0.630789	7.737716	1.990400
O	1.209579	3.128104	-2.357663
O	0.365576	7.908696	-2.712994

>

84

Coordinates from ORCA-job W-scan1 Relaxed Surface Scan Step 2

C	-2.448453	1.063109	-0.330758
C	-2.284571	-0.203256	-0.881771
C	-1.330061	1.769234	0.215750
H	-3.443134	1.529509	-0.308481
C	-0.044229	1.090321	0.188407
C	0.105315	-0.201899	-0.390300
C	-1.014136	-0.835417	-0.927315
H	-0.911360	-1.830335	-1.386890
H	-3.160986	-0.727263	-1.293193
C	-4.812041	8.725005	0.075605
C	-5.215214	7.453478	-0.371435
C	-3.449344	8.997043	0.276856
H	-5.564359	9.507422	0.259743
C	-2.488984	7.999186	0.040808
H	-3.127458	9.993301	0.616448
C	-2.886209	6.723020	-0.399459
H	-1.418592	8.204332	0.184318
C	-4.257531	6.457843	-0.612738
H	-4.575897	5.474017	-0.991796
H	-6.282011	7.240141	-0.540537
C	-1.955209	3.524730	-2.791142
C	-2.047288	4.876060	-2.400745
C	-2.160900	3.160845	-4.131023
H	-1.705443	2.745099	-2.059304
C	-2.448354	4.141017	-5.094854
H	-2.086007	2.101348	-4.420050
C	-2.527362	5.491715	-4.712287
H	-2.605156	3.854362	-6.146107
C	-2.327728	5.860001	-3.373663
H	-2.745003	6.267333	-5.462657
H	-2.381904	6.919296	-3.081394
C	4.972164	10.132036	1.253684
C	5.592103	8.941416	1.667467
C	3.844232	10.078873	0.417089
H	5.366350	11.103639	1.588863
C	3.334408	8.840732	-0.000806
H	3.350264	11.008017	0.094352
C	3.957602	7.642029	0.403099
H	2.437331	8.790858	-0.636727
C	5.091109	7.700539	1.241002
H	5.592552	6.779446	1.574270
H	6.472137	8.977081	2.328033
C	3.730248	5.642233	-2.959468
C	4.315549	5.708261	-1.681432
C	4.521724	5.370712	-4.087548
H	2.647219	5.807861	-3.060148
C	5.903114	5.165312	-3.946591
H	4.051602	5.315605	-5.081135
C	6.497967	5.251600	-2.674558
H	6.522202	4.944734	-4.829751
C	5.709252	5.529180	-1.549077
H	7.583512	5.107338	-2.560897
H	6.190055	5.618913	-0.562187
C	2.315211	1.476281	0.222943
C	2.589479	0.219859	-0.393252
C	3.336409	2.511538	0.331986
C	4.595067	2.253009	-0.301940
C	4.832185	1.033257	-0.919738
H	5.369690	3.030612	-0.306738
C	3.841964	0.012198	-0.962659
H	5.808173	0.853867	-1.396016
H	4.061491	-0.942233	-1.465464
P	-1.624798	5.410374	-0.689529
P	3.240600	6.060295	-0.218335
N	1.047271	1.808949	0.595556
N	-1.348988	3.022754	0.719228
N	2.988695	3.622995	1.004224
C	1.488652	-0.821221	-0.362426
H	1.610040	-1.548553	-1.189839
H	1.591020	-1.413386	0.580269
C	3.822068	4.805434	1.066193
H	4.902712	4.573890	0.963129
H	3.677431	5.303512	2.050006
C	-2.350118	4.002767	0.416286
H	-2.692182	4.531299	1.332781
H	-3.245763	3.585419	-0.088830
Bi	0.683826	3.835289	1.558420
W	0.778779	5.877421	-0.455991
C	1.087469	4.296996	-1.722030
C	0.512192	7.310581	-1.786441
C	0.621418	7.135549	1.132135
O	0.580713	7.801611	2.100189
O	1.290970	3.326179	-2.345608
O	0.331520	8.158012	-2.587890

>

84

Coordinates from ORCA-job W-scan1 Relaxed Surface Scan Step 3

C	-2.412682	1.016592	-0.353871
C	-2.255086	-0.299822	-0.773716
C	-1.297143	1.751646	0.161808
H	-3.398249	1.499169	-0.411014
C	-0.024221	1.046243	0.238477
C	0.115179	-0.303899	-0.190387
C	-1.000018	-0.962770	-0.704726
H	-0.908033	-2.001071	-1.058387
H	-3.126819	-0.844028	-1.168691
C	-4.978244	8.625112	0.035650
C	-5.313929	7.375933	-0.517749
C	-3.638740	8.923107	0.333574
H	-5.765183	9.370864	0.227132
C	-2.633999	7.972742	0.086705
H	-3.370123	9.903024	0.756721
C	-2.964936	6.718779	-0.459402
H	-1.579685	8.197716	0.303407
C	-4.312001	6.427241	-0.768739
H	-4.574502	5.461771	-1.229356
H	-6.362049	7.144140	-0.762481
C	-1.947172	3.602881	-2.881398
C	-2.005865	4.954360	-2.486309
C	-2.106269	3.255483	-4.232615
H	-1.755125	2.809795	-2.145761
C	-2.318523	4.251280	-5.199256
H	-2.054422	2.196372	-4.527976
C	-2.367446	5.601918	-4.809980
H	-2.439876	3.976946	-6.258416
C	-2.209510	5.954209	-3.462036
H	-2.527290	6.389069	-5.562852
H	-2.236423	7.012983	-3.163083
C	5.160728	10.023779	1.353606
C	5.849149	8.807891	1.505401
C	3.904734	10.042299	0.724876
H	5.605153	10.958588	1.728378
C	3.333492	8.848964	0.256460
H	3.359869	10.990946	0.603949
C	4.019646	7.626951	0.402615
H	2.343170	8.849826	-0.224269
C	5.285258	7.614797	1.026943
H	5.845043	6.674589	1.143551
H	6.833554	8.788068	1.998031
C	3.608959	6.102320	-3.048941
C	4.209162	5.814112	-1.807062
C	4.328836	5.935282	-4.242216
H	2.567481	6.456387	-3.070470
C	5.655365	5.476258	-4.208041
H	3.844498	6.160385	-5.204527
C	6.265492	5.198366	-2.972735
H	6.217349	5.336541	-5.144231
C	5.548228	5.372915	-1.779692
H	7.307784	4.845663	-2.937143
H	6.050940	5.169475	-0.821887
C	2.346576	1.379870	0.347982
C	2.617836	0.052548	-0.093918
C	3.388697	2.401664	0.406249
C	4.687909	2.025329	-0.066494
C	4.926020	0.732904	-0.514225
H	5.496105	2.768374	-0.078993
C	3.903250	-0.256568	-0.529937
H	5.932342	0.466546	-0.872311
H	4.127134	-1.268756	-0.900241
P	-1.639909	5.473689	-0.758110
P	3.217631	6.103531	-0.268332
N	1.068762	1.779764	0.604533
N	-1.296092	3.040491	0.556566
N	3.020485	3.597435	0.897343
C	1.479548	-0.948677	-0.033732
H	1.622292	-1.751200	-0.785118
H	1.513768	-1.457636	0.960903
C	3.856813	4.774226	0.928554
H	4.924254	4.547931	0.734294
H	3.800965	5.247842	1.934137
C	-2.325799	4.001536	0.275704
H	-2.708228	4.471266	1.208850
H	-3.195591	3.574550	-0.265272
Bi	0.729232	3.880924	1.371215
W	0.743145	6.023565	-0.477849
C	1.083434	4.554398	-1.868279
C	0.487787	7.532032	-1.723645
C	0.546774	7.197447	1.170259
O	0.478820	7.822941	2.163565
O	1.306070	3.649091	-2.574998
O	0.310745	8.422326	-2.478377

>

84

Coordinates from ORCA-job W-scan1 Relaxed Surface Scan Step 4

C	-2.258162	0.944760	-0.489332
C	-2.074575	-0.403103	-0.780280
C	-1.174204	1.727378	0.023205
H	-3.239494	1.414237	-0.645075
C	0.096532	1.036438	0.225267
C	0.257415	-0.349261	-0.054987
C	-0.828275	-1.054245	-0.572786
H	-0.720890	-2.121514	-0.820040
H	-2.920547	-0.984455	-1.178420
C	-5.212084	8.418083	-0.041486
C	-5.457400	7.169787	-0.642402
C	-3.900007	8.790473	0.292673
H	-6.048456	9.105557	0.158396
C	-2.832264	7.914427	0.035484
H	-3.702458	9.770679	0.752613
C	-3.073417	6.661119	-0.557065
H	-1.797987	8.197835	0.279038
C	-4.392571	6.295426	-0.904244
H	-4.584446	5.331603	-1.401715
H	-6.483978	6.881116	-0.916176
C	-1.890870	3.705402	-3.061648
C	-1.960705	5.043609	-2.626982
C	-1.998012	3.402725	-4.429154
H	-1.725873	2.888155	-2.344940
C	-2.171720	4.429904	-5.369764
H	-1.935464	2.354070	-4.757942
C	-2.230347	5.768033	-4.939878
H	-2.252417	4.190367	-6.441095

C	-2.119942	6.075489	-3.576836
H	-2.357697	6.579265	-5.673128
H	-2.147976	7.124243	-3.242364
C	5.472860	9.910443	1.339249
C	6.183572	8.811211	0.823660
C	4.069100	9.887598	1.368147
H	6.017752	10.791641	1.711578
C	3.373058	8.765583	0.888312
H	3.509017	10.750003	1.760655
C	4.079710	7.660175	0.379382
H	2.273518	8.740711	0.890048
C	5.491062	7.691038	0.341476
H	6.048472	6.844284	-0.089263
H	7.283639	8.832282	0.788908
C	4.089235	6.928764	-2.762436
C	3.999604	5.867257	-1.836253
C	4.617494	6.705720	-4.041251
H	3.733326	7.931255	-2.478342
C	5.050827	5.420026	-4.412892
H	4.686536	7.540422	-4.755517
C	4.949327	4.359361	-3.499396
H	5.459751	5.244825	-5.419722
C	4.424417	4.578693	-2.214931
H	5.272137	3.346925	-3.786720
H	4.326635	3.727948	-1.525708
C	2.456407	1.367828	0.545379
C	2.755509	0.001476	0.284178
C	3.499616	2.383796	0.656789
C	4.849830	1.942753	0.476157
C	5.120512	0.607016	0.197373
H	5.672923	2.665341	0.566326
C	4.087621	-0.364101	0.094997
H	6.165920	0.291080	0.056957
H	4.341551	-1.410855	-0.132122
P	-1.660844	5.520015	-0.874077
P	3.150665	6.191395	-0.234886
N	1.163955	1.802240	0.593690
N	-1.194409	3.038985	0.328066
N	3.083591	3.640237	0.905649
C	1.600422	-0.984588	0.260597
H	1.812581	-1.815748	-0.442678
H	1.530367	-1.460924	1.269084
C	3.899039	4.826877	0.890046
H	4.937344	4.643148	0.544145
H	3.963610	5.286146	1.901953
C	-2.270471	3.958761	0.062993
H	-2.717873	4.340006	1.008311
H	-3.092050	3.512849	-0.534843
Bi	0.784209	3.940664	1.179922
W	0.687687	6.195842	-0.476216
C	1.067595	5.073258	-2.154894
C	0.603204	7.883226	-1.501250
C	0.314439	7.195204	1.257032
O	0.099744	7.753841	2.269988
O	1.284856	4.403602	-3.084630
O	0.555972	8.888432	-2.113766

>
84

Coordinates from ORCA-job W-scan1 Relaxed Surface Scan Step 5

C	-2.274256	0.871713	-0.403997
C	-2.111428	-0.501734	-0.571392
C	-1.168241	1.681197	-0.000116
H	-3.252362	1.340214	-0.582812
C	0.097443	0.994466	0.239680
C	0.239591	-0.411282	0.090664
C	-0.868813	-1.146741	-0.333225
H	-0.780407	-2.234087	-0.481041
H	-2.972736	-1.106464	-0.894889
C	-5.272715	8.325530	0.003555
C	-5.490606	7.091334	-0.635810
C	-3.968209	8.720335	0.341743
H	-6.124801	8.984651	0.230628
C	-2.880542	7.880633	0.049549
H	-3.792275	9.689798	0.832202
C	-3.094220	6.641192	-0.581550
H	-1.851748	8.181874	0.295420
C	-4.406049	6.252795	-0.931805
H	-4.577414	5.300303	-1.457679
H	-6.511426	6.785457	-0.912418
C	-1.795393	3.774422	-3.157399
C	-1.951581	5.098493	-2.700304
C	-1.899678	3.486482	-4.527976
H	-1.562840	2.962494	-2.452789
C	-2.156831	4.514149	-5.448968
H	-1.769781	2.450227	-4.875757
C	-2.302950	5.837721	-4.996404
H	-2.235007	4.286455	-6.523033
C	-2.195699	6.131261	-3.629752
H	-2.496990	6.649050	-5.714669
H	-2.294252	7.169686	-3.277054
C	5.540689	9.828729	1.432171
C	6.234623	8.739544	0.874000
C	4.136532	9.830454	1.452625
H	6.099375	10.682911	1.844843
C	3.423412	8.742510	0.922274
H	3.589613	10.685306	1.878625
C	4.113083	7.646831	0.370799
H	2.323726	8.736334	0.918266
C	5.524985	7.653267	0.341505
H	6.070443	6.814949	-0.119936
H	7.335256	8.741721	0.846483
C	4.159187	7.028051	-2.793230
C	3.996074	5.935053	-1.916189
C	4.675204	6.826492	-4.080929
H	3.870696	8.038485	-2.464177
C	5.022302	5.532494	-4.509053
H	4.801963	7.685160	-4.757996
C	4.847615	4.441161	-3.643695
H	5.420873	5.375125	-5.522931
C	4.335150	4.638072	-2.350995
H	5.103155	3.423567	-3.976665
H	4.174971	3.768485	-1.696885
C	2.464932	1.325042	0.551875
C	2.749664	-0.060838	0.422334
C	3.516492	2.335351	0.618026
C	4.864744	1.868713	0.540300

C	5.126219	0.508989	0.386389
H	5.692520	2.589277	0.601279
C	4.085255	-0.454961	0.322231
H	6.171512	0.170465	0.315885
H	4.333873	-1.519984	0.196107
P	-1.654158	5.548280	-0.939973
P	3.158579	6.221004	-0.301744
N	1.176726	1.777485	0.534137
N	-1.157060	3.019922	0.175718
N	3.100516	3.614307	0.736369
C	1.583320	-1.036933	0.431600
H	1.791994	-1.892292	-0.244248
H	1.510588	-1.482705	1.453266
C	3.920493	4.796132	0.749812
H	4.939804	4.620698	0.345764
H	4.038799	5.208275	1.777746
C	-2.241534	3.934907	-0.062678
H	-2.724219	4.263239	0.885846
H	-3.037139	3.503861	-0.706045
Bi	0.822543	3.944139	0.926383
W	0.686285	6.292069	-0.542410
C	1.048889	5.388394	-2.353197
C	0.585265	8.050853	-1.438058
C	0.314624	7.207283	1.238506
O	0.097190	7.746813	2.260049
O	1.252943	4.879357	-3.381333
O	0.529907	9.098103	-1.973258

>

84

Coordinates from ORCA-job W-scan1 Relaxed Surface Scan Step 6

C	-2.303847	0.809961	-0.280708
C	-2.165822	-0.578140	-0.310252
C	-1.169023	1.633423	-0.022821
H	-3.282342	1.277190	-0.461717
C	0.096883	0.954847	0.229151
C	0.217926	-0.458556	0.223553
C	-0.922005	-1.214673	-0.066020
H	-0.855256	-2.313141	-0.100076
H	-3.049270	-1.198595	-0.526407
C	-5.317159	8.227426	0.049109
C	-5.511342	7.017131	-0.641437
C	-4.020446	8.632852	0.404248
H	-6.181768	8.859710	0.303432
C	-2.916764	7.827922	0.076731
H	-3.863135	9.583817	0.935493
C	-3.106631	6.612316	-0.606392
H	-1.893971	8.138485	0.335758
C	-4.410984	6.212672	-0.971750
H	-4.564492	5.279374	-1.536000
H	-6.526138	6.703027	-0.930596
C	-1.661394	3.860688	-3.269538
C	-1.933465	5.154471	-2.779300
C	-1.754005	3.596818	-4.645249
H	-1.346948	3.061437	-2.581564
C	-2.113992	4.618753	-5.538438
H	-1.534770	2.585438	-5.020399
C	-2.375902	5.911855	-5.052421
H	-2.182376	4.410498	-6.617076
C	-2.282120	6.182100	-3.679502
H	-2.650844	6.718615	-5.749105
H	-2.473118	7.198011	-3.300507
C	5.594107	9.742870	1.492732
C	6.274348	8.664891	0.897255
C	4.190083	9.763422	1.510456
H	6.163516	10.573628	1.937286
C	3.463366	8.705591	0.939342
H	3.653924	10.609626	1.966275
C	4.139335	7.621238	0.349817
H	2.363637	8.714118	0.932936
C	5.551293	7.608340	0.324530
H	6.086842	6.779351	-0.164376
H	7.374950	8.652369	0.872560
C	4.239018	7.114796	-2.822650
C	3.989702	5.993680	-2.004455
C	4.743493	6.940596	-4.119327
H	4.028393	8.126026	-2.441456
C	4.993782	5.648089	-4.613287
H	4.937688	7.820715	-4.751370
C	4.733893	4.529620	-3.805296
H	5.383671	5.513399	-5.633753
C	4.231942	4.698084	-2.504913
H	4.915041	3.514416	-4.190323
H	4.002157	3.813388	-1.892201
C	2.472526	1.285460	0.535347
C	2.741274	-0.107382	0.548203
C	3.530784	2.287582	0.583616
C	4.872262	1.808903	0.644430
C	5.124357	0.436715	0.628191
H	5.702345	2.527694	0.697113
C	4.077298	-0.518759	0.576912
H	6.167008	0.084472	0.659403
H	4.319082	-1.592837	0.564470
P	-1.645664	5.564689	-1.008423
P	3.165406	6.236562	-0.376603
N	1.191082	1.749885	0.428764
N	-1.128725	2.986992	0.013888
N	3.120363	3.578301	0.562798
C	1.568783	-1.079196	0.554068
H	1.775215	-1.924104	-0.137503
H	1.503560	-1.545551	1.565866
C	3.938618	4.758817	0.608282
H	4.941357	4.599236	0.156498
H	4.097121	5.128138	1.647629
C	-2.217206	3.902198	-0.192836
H	-2.726423	4.181518	0.758173
H	-2.991499	3.493494	-0.877243
Bi	0.860327	3.924669	0.646118
W	0.684994	6.361894	-0.609181
C	1.029464	5.709680	-2.529579
C	0.536656	8.193350	-1.337734
C	0.327282	7.155881	1.232572
O	0.117246	7.653926	2.275510
O	1.221505	5.381083	-3.630187
O	0.450222	9.284280	-1.770355

>

84

Coordinates from ORCA-job W-scan1 Relaxed Surface Scan Step 7

C	-2.320628	0.775268	-0.161385
C	-2.205010	-0.613287	-0.056573
C	-1.161807	1.593602	-0.054967
H	-3.300554	1.243874	-0.331602
C	0.106473	0.920819	0.189295
C	0.209782	-0.486040	0.317311
C	-0.959095	-1.244466	0.177518
H	-0.909391	-2.341769	0.254432
H	-3.108806	-1.234173	-0.155278
C	-5.341045	8.134913	0.097729
C	-5.520109	6.952707	-0.643565
C	-4.049337	8.542218	0.468649
H	-6.213628	8.744016	0.379791
C	-2.935419	7.767436	0.105362
H	-3.903940	9.471450	1.040165
C	-3.110302	6.579547	-0.628835
H	-1.916742	8.080998	0.376504
C	-4.409755	6.177586	-1.008786
H	-4.551867	5.266529	-1.611006
H	-6.531022	6.637385	-0.944634
C	-1.587655	3.938922	-3.382297
C	-1.921273	5.203919	-2.855507
C	-1.671540	3.710044	-4.764655
H	-1.235168	3.137954	-2.714310
C	-2.083688	4.738279	-5.627933
H	-1.405566	2.721416	-5.169136
C	-2.407988	6.002254	-5.104938
H	-2.144474	4.557777	-6.711989
C	-2.324017	6.237962	-3.724832
H	-2.724187	6.813914	-5.778007
H	-2.564035	7.232063	-3.317047
C	5.625717	9.667366	1.537039
C	6.298492	8.609573	0.898579
C	4.221886	9.692675	1.563142
H	6.200771	10.478784	2.009138
C	3.487913	8.659864	0.956846
H	3.691530	10.523267	2.053113
C	4.156653	7.595397	0.324121
H	2.388188	8.672934	0.956810
C	5.568408	7.577313	0.291462
H	6.098241	6.764315	-0.229333
H	7.398862	8.593500	0.867817
C	4.293793	7.166890	-2.858368
C	3.983031	6.031686	-2.082517
C	4.780790	7.012749	-4.164618
H	4.144891	8.173476	-2.437973
C	4.953111	5.727923	-4.708611
H	5.022446	7.903492	-4.764594
C	4.633294	4.595999	-3.941246
H	5.329338	5.609938	-5.736222
C	4.147769	4.743504	-2.632469
H	4.755272	3.587559	-4.365385
H	3.870465	3.853226	-2.047269
C	2.488444	1.253970	0.502499
C	2.739479	-0.132144	0.650448
C	3.547024	2.252202	0.563801
C	4.871150	1.781207	0.784664
C	5.111669	0.410176	0.906764
H	5.698345	2.502336	0.852009
C	4.065250	-0.541628	0.839380
H	6.143703	0.060122	1.063144
H	4.295604	-1.613631	0.940397
P	-1.637223	5.569064	-1.074874
P	3.173080	6.242030	-0.444012
N	1.216375	1.717980	0.291810
N	-1.101024	2.948781	-0.146419
N	3.148224	3.542921	0.409983
C	1.572235	-1.110747	0.594249
H	1.782266	-1.889187	-0.173317
H	1.523324	-1.668080	1.557664
C	3.952506	4.729191	0.497281
H	4.952152	4.595649	0.028277
H	4.116824	5.066847	1.546891
C	-2.190577	3.870197	-0.306579
H	-2.698560	4.113950	0.655318
H	-2.965624	3.488378	-1.007022
Bi	0.905824	3.885406	0.348318
W	0.687859	6.399199	-0.674489
C	1.009314	5.952108	-2.657426
C	0.505829	8.281446	-1.250569
C	0.343272	7.068077	1.219496
O	0.138637	7.515114	2.285440
O	1.183363	5.763852	-3.792929
O	0.398472	9.402242	-1.590942

>

84

Coordinates from ORCA-job W-scan1 Relaxed Surface Scan Step 8

C	-2.332854	0.769011	-0.027442
C	-2.234381	-0.607597	0.200907
C	-1.157590	1.565670	-0.072760
H	-3.313866	1.243613	-0.173979
C	0.112443	0.891561	0.142801
C	0.202984	-0.499294	0.386218
C	-0.986430	-1.240875	0.406300
H	-0.947549	-2.326770	0.584679
H	-3.153419	-1.213223	0.224794
C	-5.337459	8.066135	0.142768
C	-5.513220	6.916760	-0.649075
C	-4.046831	8.461178	0.530509
H	-6.211799	8.659548	0.451491
C	-2.930595	7.707146	0.132719
H	-3.904120	9.365040	1.141931
C	-3.102477	6.551626	-0.652255
H	-1.913002	8.012712	0.416483
C	-4.400748	6.161534	-1.048067
H	-4.540106	5.276108	-1.687944
H	-6.523272	6.611345	-0.962877
C	-1.578264	3.982812	-3.479194
C	-1.921925	5.232981	-2.924157
C	-1.664537	3.783446	-4.866099
H	-1.220361	3.170106	-2.828167
C	-2.089286	4.826286	-5.705500
H	-1.391743	2.806102	-5.292876
C	-2.425193	6.074964	-5.153928
H	-2.151535	4.669021	-6.793069

C	-2.339050	6.281402	-3.769296
H	-2.751864	6.897807	-5.808117
H	-2.587698	7.263988	-3.339303
C	5.624711	9.603758	1.575052
C	6.299994	8.572527	0.897145
C	4.220885	9.620542	1.608561
H	6.197676	10.401385	2.072467
C	3.489440	8.605849	0.969737
H	3.688506	10.430629	2.129618
C	4.161030	7.567657	0.297412
H	2.389725	8.613782	0.974901
C	5.572647	7.557754	0.258071
H	6.104021	6.765521	-0.292256
H	7.400246	8.563400	0.860896
C	4.311914	7.210928	-2.900246
C	3.995999	6.059520	-2.150654
C	4.798094	7.084641	-4.209757
H	4.166842	8.208355	-2.457266
C	4.965178	5.811891	-4.782900
H	5.042951	7.987846	-4.789419
C	4.642069	4.663970	-4.040990
H	5.340668	5.715861	-5.813043
C	4.157032	4.783347	-2.729039
H	4.762472	3.664931	-4.487282
H	3.881245	3.881415	-2.160778
C	2.500667	1.225405	0.454132
C	2.730712	-0.146121	0.714971
C	3.553388	2.224413	0.542078
C	4.849610	1.773028	0.908296
C	5.072331	0.413550	1.151309
H	5.670403	2.499369	0.997027
C	4.031525	-0.539712	1.058463
H	6.084163	0.077760	1.425957
H	4.243630	-1.601507	1.258712
P	-1.628336	5.563467	-1.138992
P	3.182374	6.235167	-0.510616
N	1.239994	1.677887	0.144932
N	-1.083574	2.911984	-0.279812
N	3.172920	3.507561	0.278472
C	1.576944	-1.132101	0.573848
H	1.783279	-1.802146	-0.293849
H	1.556525	-1.807743	1.456654
C	3.955784	4.702962	0.411341
H	4.967273	4.596454	-0.040656
H	4.091707	5.021851	1.471148
C	-2.166689	3.847040	-0.389946
H	-2.646434	4.078616	0.589643
H	-2.964120	3.489229	-1.079087
Bi	0.952544	3.828223	0.041974
W	0.696289	6.403480	-0.747584
C	0.999766	6.124656	-2.763952
C	0.482638	8.321498	-1.172660
C	0.369533	6.931249	1.194932
O	0.175083	7.303317	2.290201
O	1.163468	6.033785	-3.912698
O	0.355288	9.463298	-1.423776

>

84

Coordinates from ORCA-job W-scan1 Relaxed Surface Scan Step 9

C	-2.329524	0.780370	0.060938
C	-2.239378	-0.583045	0.365378
C	-1.148085	1.553270	-0.086885
H	-3.310044	1.262746	-0.062240
C	0.120258	0.872391	0.099227
C	0.204932	-0.503629	0.413325
C	-0.991862	-1.223263	0.543518
H	-0.956957	-2.296361	0.787825
H	-3.164647	-1.169686	0.473326
C	-5.313472	8.026464	0.182631
C	-5.495322	6.908734	-0.651856
C	-4.020680	8.400728	0.583769
H	-6.184740	8.611815	0.514509
C	-2.908256	7.657724	0.156067
H	-3.873333	9.280122	1.228820
C	-3.086626	6.533520	-0.671919
H	-1.889182	7.948296	0.449858
C	-4.386819	6.163815	-1.080373
H	-4.530277	5.302865	-1.751939
H	-6.507069	6.619860	-0.975631
C	-1.625706	3.991154	-3.549103
C	-1.934534	5.242899	-2.977607
C	-1.728438	3.809241	-4.937640
H	-1.286349	3.160938	-2.910252
C	-2.136303	4.870601	-5.761865
H	-1.483475	2.830124	-5.377073
C	-2.439464	6.120358	-5.193683
H	-2.211798	4.726709	-6.850435
C	-2.335889	6.309499	-3.808011
H	-2.753415	6.957412	-5.835900
H	-2.557609	7.292928	-3.365195
C	5.607949	9.563185	1.600857
C	6.288877	8.563665	0.882475
C	4.204492	9.559127	1.651784
H	6.175962	10.352762	2.116429
C	3.479005	8.555628	0.989135
H	3.667646	10.344720	2.204750
C	4.156808	7.549031	0.275980
H	2.379525	8.549350	1.007550
C	5.567763	7.559408	0.220093
H	6.102408	6.791466	-0.360643
H	7.388605	8.571034	0.832983
C	4.308919	7.233799	-2.938066
C	4.016832	6.071513	-2.195015
C	4.803775	7.125814	-4.245711
H	4.137053	8.225351	-2.491300
C	5.003418	5.860138	-4.824389
H	5.029183	8.037438	-4.819989
C	4.705669	4.701335	-4.089050
H	5.385276	5.778106	-5.853390
C	4.212574	4.802512	-2.778190
H	4.853837	3.707558	-4.538762
H	3.961765	3.890757	-2.213976
C	2.510224	1.207675	0.415953
C	2.723865	-0.150348	0.746696
C	3.550815	2.212525	0.535799
C	4.819112	1.783079	1.007090

C	5.026772	0.435994	1.326379
H	5.630463	2.516635	1.120777
C	3.996400	-0.523736	1.203216
H	6.017045	0.117996	1.687159
H	4.192007	-1.574371	1.468428
P	-1.619105	5.554554	-1.193133
P	3.189098	6.226962	-0.560293
N	1.260422	1.643752	0.032151
N	-1.067186	2.888601	-0.368146
N	3.185892	3.485447	0.196637
C	1.582680	-1.140516	0.548534
H	1.788416	-1.738439	-0.371915
H	1.576786	-1.880233	1.376967
C	3.946079	4.690141	0.365692
H	4.976729	4.603639	-0.047054
H	4.033849	5.004706	1.432001
C	-2.141504	3.836485	-0.438899
H	-2.579015	4.075322	0.558718
H	-2.970439	3.491343	-1.097603
Bi	0.998423	3.768250	-0.241780
W	0.707280	6.391598	-0.814623
C	0.992272	6.260025	-2.847767
C	0.479048	8.331695	-1.099788
C	0.393257	6.778706	1.164169
O	0.205703	7.071512	2.283601
O	1.144546	6.252075	-4.001888
O	0.343069	9.488202	-1.264025

3-TS

C	-2.328293	0.779823	0.068879
C	-2.242342	-0.576963	0.398102
C	-1.145771	1.548507	-0.085880
H	-3.307448	1.260743	-0.068967
C	0.119375	0.870192	0.121038
C	0.199792	-0.499731	0.459346
C	-0.998023	-1.214864	0.594764
H	-0.965669	-2.283298	0.858448
H	-3.169023	-1.160057	0.511174
C	-5.318419	7.979902	0.222704
C	-5.503204	6.859277	-0.605094
C	-4.023054	8.371778	0.592995
H	-6.189711	8.553686	0.573651
C	-2.911054	7.643132	0.142268
H	-3.872762	9.254097	1.233154
C	-3.091145	6.515353	-0.679402
H	-1.890222	7.948733	0.414678
C	-4.395263	6.129403	-1.057493
H	-4.543808	5.265912	-1.724658
H	-6.517665	6.555647	-0.905543
C	-1.701951	4.004549	-3.610015
C	-1.976919	5.253677	-3.017257
C	-1.854923	3.834153	-4.994833
H	-1.351378	3.163550	-2.991412
C	-2.279427	4.904719	-5.796385
H	-1.636876	2.855589	-5.449071
C	-2.548033	6.152166	-5.209040
H	-2.395765	4.769444	-6.882303
C	-2.394669	6.329363	-3.827106
H	-2.876113	6.997323	-5.833093
H	-2.594580	7.311480	-3.371119
C	5.614915	9.523111	1.637477
C	6.297885	8.520706	0.927602
C	4.212025	9.535334	1.658099
H	6.181121	10.302203	2.170361
C	3.489071	8.544978	0.974640
H	3.672689	10.323583	2.204587
C	4.168085	7.534536	0.269323
H	2.389245	8.552591	0.971989
C	5.579462	7.530265	0.243645
H	6.118743	6.760559	-0.330400
H	7.398274	8.514536	0.901277
C	4.363592	7.263642	-2.943598
C	4.063641	6.090602	-2.221421
C	4.899819	7.177412	-4.235738
H	4.169279	8.248663	-2.491518
C	5.134743	5.922567	-4.821742
H	5.131759	8.098718	-4.791402
C	4.829839	4.752864	-4.109267
H	5.550843	5.857380	-5.838435
C	4.294958	4.832574	-2.813996
H	5.006050	3.766325	-4.564421
H	4.042070	3.909321	-2.269530
C	2.506804	1.205637	0.439734
C	2.716534	-0.146282	0.794546
C	3.549823	2.208335	0.540908
C	4.815684	1.783302	1.020735
C	5.019124	0.442755	1.364508
H	5.629404	2.515951	1.121520
C	3.986911	-0.514906	1.257994
H	6.008012	0.128581	1.731819
H	4.178678	-1.561105	1.542269
P	-1.622746	5.546479	-1.232297
P	3.204817	6.221331	-0.595640
N	1.260174	1.637050	0.046235
N	-1.062863	2.877491	-0.391834
N	3.190991	3.475410	0.177369
C	1.573686	-1.135205	0.616189
H	1.781411	-1.758093	-0.286753
H	1.561927	-1.854180	1.462549
C	3.955345	4.677307	0.329359
H	4.983489	4.583766	-0.088778
H	4.053628	4.999456	1.392727
C	-2.136379	3.821708	-0.480105
H	-2.586120	4.063495	0.511604
H	-2.958879	3.470825	-1.144268
Bi	1.004575	3.755347	-0.270936
W	0.714581	6.382855	-0.859416
C	0.999160	6.256263	-2.891591
C	0.486345	8.321976	-1.144483
C	0.398528	6.786263	1.115413
O	0.209006	7.097392	2.228987
O	1.150781	6.254113	-4.045713
O	0.350084	9.478493	-1.304526

4

170

Au	5.390510	12.445974	9.915442

Au	6.262749	6.805767	7.823897
Au	0.557476	8.732277	9.106963
Cl	4.364545	8.739757	10.525611
Cl	4.033731	9.957498	7.357915
Bi	6.801430	9.789140	8.834340
Bi	2.479990	11.164970	9.840770
Bi	3.104130	7.062020	8.163520
P	5.975450	11.823800	12.114840
P	4.954710	13.503400	7.849920
P	6.377910	5.689600	9.901290
P	6.417520	7.560700	5.593950
P	0.123020	9.329330	6.864580
P	0.545510	8.063530	11.371090
N	7.677770	9.992840	11.049460
N	9.060520	10.164200	8.746010
N	1.128430	12.807390	10.705830
N	2.306450	5.056100	7.381350
N	2.321150	12.990260	8.297880
C	9.692530	10.200150	7.523520
N	3.694150	5.302210	9.676690
N	7.524140	9.850850	6.557040
N	1.621650	10.482210	11.965480
N	1.886010	7.401630	6.133380
C	1.322270	6.298690	5.564170
C	4.592150	11.792120	13.317380
C	0.946520	13.976140	10.001370
C	1.578300	5.024430	6.213450
C	0.978210	6.308130	11.677310
C	6.795000	10.147540	12.174230
H	7.279060	10.029150	13.169060
H	5.954460	9.425470	12.103340
C	1.713340	9.108140	12.383650
H	1.502820	8.931590	13.461910
H	2.724750	8.711150	12.153330
C	-0.083050	11.124570	6.553390
C	1.480200	8.724170	5.737140
H	1.132110	8.812100	4.683930
H	2.318460	9.436730	5.889900
C	6.145540	13.146770	6.502760
C	4.691310	5.519610	10.691000
H	4.745990	4.728260	11.471310
H	4.522300	6.495990	11.189560
C	3.247400	13.098040	7.202400
H	2.977580	13.855050	6.432920
H	3.367700	12.115040	6.703040
C	9.005340	10.283320	11.155180
C	7.492140	6.435120	11.151850
C	8.863000	9.996870	6.347240
C	0.116190	11.101990	13.831640
H	0.271110	10.148900	14.354780
C	7.896540	6.828890	4.784510
C	0.772560	11.346370	12.590650
C	4.985920	15.328960	8.067490
C	11.095370	10.402680	7.399220
C	1.471860	15.295400	7.985710
H	1.958430	15.407640	7.007610
C	1.610920	14.078510	8.713760
C	6.654100	9.410480	5.500080
H	6.997520	9.668600	4.473700

H	5.634310	9.825260	5.644840
C	11.973820	10.654390	8.630690
C	0.532410	12.618950	11.932380
C	1.067670	3.814150	5.666220
C	2.536650	1.485930	8.354280
H	2.226680	0.487940	8.012550
C	2.589500	3.899220	8.071840
C	3.702610	2.847570	10.004380
H	4.281840	2.912820	10.935080
C	13.081820	9.569150	8.691270
H	12.633670	8.559910	8.781790
H	13.708310	9.588020	7.777840
H	13.747620	9.729740	9.562170
C	-0.912970	13.309660	13.759720
H	-1.563350	14.056530	14.237250
C	11.161970	10.603490	9.930490
C	11.668740	10.379230	6.121370
H	12.751130	10.534170	6.005840
C	4.992780	7.143510	4.518680
C	-1.420980	8.531150	6.268150
C	0.517070	6.299540	4.388500
H	0.298770	7.245070	3.874290
C	0.057240	16.222630	9.739310
H	-0.541760	17.065540	10.112940
C	-1.118440	8.310230	12.110870
C	-0.305790	13.600570	12.531430
C	2.176830	2.617380	7.611300
C	-0.534990	14.961780	11.864900
C	10.881350	10.160290	4.972030
C	6.994200	3.974890	9.654650
C	-2.057740	15.178900	11.660890
H	-2.478560	14.392890	11.003170
H	-2.599310	15.147330	12.626720
H	-2.260120	16.163160	11.194640
C	11.070030	10.783920	12.370730
C	0.294890	3.875370	4.499530
H	-0.107280	2.950120	4.062470
C	3.358890	4.035530	9.296410
C	7.212500	13.002390	12.792200
C	-0.706480	12.070700	14.400950
C	0.705280	16.341690	8.492550
C	11.794870	10.818750	11.161450
H	12.875250	11.020670	11.191560
C	1.358990	2.460320	6.323870
C	9.505720	9.969140	5.075500
H	8.912660	9.813090	4.164360
C	0.019450	5.106780	3.869810
C	2.165200	1.589490	5.322420
H	2.382670	0.590330	5.749240
H	1.599550	1.443750	4.380820
H	3.129430	2.075310	5.073260
C	9.763040	10.344930	9.916170
C	3.293600	1.600590	9.538520
C	0.166350	15.054740	10.504720
C	0.015470	1.763730	6.667410
H	-0.571300	2.378600	7.378190
H	-0.597780	1.607490	5.758140
H	0.189050	0.772850	7.131390
C	0.036940	16.066230	12.795190

H	-0.454030	16.040420	13.788340
H	1.125380	15.922630	12.945450
H	-0.120770	17.073350	12.360630
C	12.618330	12.060750	8.497630
H	13.227470	12.130820	7.574680
H	11.836460	12.844660	8.454520
H	13.278680	12.279500	9.360040
C	9.701540	10.525590	12.374840
H	9.161450	10.492970	13.330670
H	4.813130	15.846770	7.102600
H	5.968630	15.632380	8.479370
H	4.191240	15.616130	8.782780
H	5.828010	13.619470	5.551750
H	6.227150	12.048970	6.383410
H	7.143220	13.531910	6.791150
H	7.963690	7.145580	3.724270
H	7.838980	5.724000	4.838720
H	8.801020	7.170120	5.324440
H	6.284560	3.430560	9.002270
H	7.081990	3.442320	10.622980
H	7.985320	4.009670	9.160710
H	8.534940	6.384910	10.781750
H	7.418850	5.901470	12.120640
H	7.228250	7.503710	11.274900
H	3.793200	11.130040	12.925130
H	4.169240	12.811830	13.409250
H	4.932820	11.443070	14.312770
H	-1.642630	8.829000	5.223670
H	-2.263510	8.824460	6.924840
H	-1.293520	7.432390	6.314350
H	8.126450	12.952960	12.169070
H	7.471260	12.745420	13.839010
H	6.803770	14.031160	12.752640
H	-0.189410	11.335000	5.470270
H	0.790610	11.666260	6.972130
H	-0.986630	11.480060	7.086680
H	5.111880	7.581030	3.507200
H	4.063470	7.515050	4.997050
H	4.912190	6.041780	4.436290
H	0.197060	5.663130	11.228890
H	1.050250	6.095780	12.762900
H	1.938140	6.077200	11.170360
H	-1.868840	7.742130	11.526690
H	-1.368890	9.387790	12.068900
H	-1.135630	7.972690	13.166580
H	11.359420	10.146110	3.980690
H	11.593490	10.958130	13.323180
H	-0.590970	5.124340	2.954080
H	3.559860	0.694360	10.103590
H	0.600210	17.270620	7.911630
H	-1.196680	11.869670	15.365710

COF_2

F	-7.865790	1.103859	-0.000012
C	-6.745771	1.821083	-0.000153
F	-5.728540	0.964265	-0.000012
O	-6.668989	2.997673	-0.000364

$[COF_3]^-$ -

F	-7.843547	1.509454	0.841369
C	-6.725198	1.509468	-0.083413
F	-5.697268	0.911309	0.748168
O	-6.456989	2.558764	-0.640669
F	-7.077039	0.377675	-0.920735

BH_3

H	-7.821972	1.193262	0.000069
B	-6.752244	1.722119	-0.000425
H	-5.760377	1.058646	0.000069
H	-6.674497	2.912853	-0.000252

$[BH_3F]^-$ -

F	-7.880583	1.565776	0.853415
B	-6.726848	1.367658	-0.036650
H	-5.743366	0.990323	0.628633
H	-6.448930	2.438942	-0.608835
H	-7.000312	0.503971	-0.891843

2+F^-

C	-1.673744	1.137943	-1.119066
C	-1.137526	-0.046644	-1.655106
C	-0.858479	2.038325	-0.385659
H	-2.736731	1.373559	-1.275311
C	0.522907	1.677255	-0.172601
C	1.046496	0.477921	-0.701655
C	0.211828	-0.373499	-1.454452
H	0.628524	-1.307735	-1.865269
H	-1.789747	-0.725014	-2.228819
C	-7.308837	6.103479	-0.664420
C	-6.330532	6.891776	-1.291017
C	-6.914552	5.134117	0.272840
H	-8.374448	6.245978	-0.901827
C	-5.558227	4.958677	0.580897
H	-7.671243	4.511609	0.775860
C	-4.560898	5.731345	-0.060717
H	-5.267158	4.209715	1.334714
C	-4.970889	6.706943	-0.996020
H	-4.214367	7.322784	-1.505145
H	-6.626506	7.657104	-2.025996
C	-1.891680	5.961781	-2.251550
C	-1.887710	6.369441	-0.900797
C	-1.165898	6.678615	-3.213777
H	-2.464274	5.070394	-2.551733
C	-0.420339	7.809461	-2.840038
H	-1.162684	6.338438	-4.260680
C	-0.400742	8.220892	-1.498660
H	0.171967	8.353144	-3.591409
C	-1.132671	7.504871	-0.537527
H	0.211432	9.082752	-1.194096
H	-1.105507	7.812246	0.520557
C	8.005115	7.878741	-1.272839
C	7.447919	7.883893	0.015117
C	7.191603	7.535824	-2.367133
H	9.062167	8.146324	-1.425093
C	5.846720	7.194276	-2.174263
H	7.609763	7.536225	-3.386484
C	5.274044	7.167518	-0.877342
H	5.221733	6.939698	-3.043809
C	6.101493	7.539337	0.209783
H	5.690420	7.561152	1.230973
H	8.067807	8.158108	0.883668

C	1.746142	5.113001	-1.898733
C	3.118016	5.404289	-1.718974
C	1.335326	3.932364	-2.541034
H	0.990179	5.823821	-1.533266
C	2.291862	3.044848	-3.049238
H	0.263099	3.699511	-2.617843
C	3.660901	3.350795	-2.927183
H	1.969975	2.106099	-3.524688
C	4.070722	4.507628	-2.253532
H	4.419596	2.648860	-3.304910
H	5.143024	4.687528	-2.096459
C	2.700141	2.485031	0.432539
C	3.334671	1.322301	-0.059476
C	3.472736	3.651271	0.777661
C	4.879996	3.592740	0.625974
C	5.500394	2.435298	0.131161
H	5.487631	4.480637	0.855009
C	4.733697	1.311172	-0.217248
H	6.594963	2.417670	0.005802
H	5.220830	0.404229	-0.611643
P	-2.800783	5.481867	0.448504
P	3.474336	6.861246	-0.649149
N	1.320224	2.584029	0.504201
N	-1.258197	3.246829	0.125932
N	2.759256	4.752361	1.187685
C	2.478429	0.112157	-0.368235
H	2.929450	-0.478837	-1.195128
H	2.480578	-0.573612	0.517074
C	3.339839	6.035413	1.158387
H	4.368221	6.083331	1.589292
H	2.712154	6.756113	1.724700
C	-2.598860	3.681102	-0.080320
H	-3.356838	3.113736	0.523252
H	-2.941287	3.580665	-1.144221
Bi	0.422366	4.491160	1.225158
F	0.195194	3.799818	3.209842

3+F⁻

C	-2.425778	1.024871	-0.311219
C	-2.276217	-0.271456	-0.826503
C	-1.297580	1.759154	0.142501
H	-3.424773	1.484081	-0.265249
C	-0.009150	1.102644	0.082667
C	0.128667	-0.212470	-0.415146
C	-1.012793	-0.887848	-0.882595
H	-0.909018	-1.906680	-1.289180
H	-3.165144	-0.813105	-1.187549
C	-4.980629	8.656742	0.061754
C	-5.334570	7.366786	-0.370738
C	-3.628532	8.983388	0.247878
H	-5.762574	9.409529	0.248366
C	-2.631678	8.023024	0.010768
H	-3.343232	9.994075	0.578689
C	-2.977944	6.728630	-0.417878
H	-1.567441	8.266645	0.146263
C	-4.339486	6.409454	-0.613646
H	-4.619927	5.408264	-0.977262
H	-6.393747	7.107604	-0.525929
C	-2.059342	3.576967	-2.805449
C	-2.061188	4.932183	-2.419010
C	-2.268715	3.226004	-4.148647
H	-1.880738	2.779806	-2.069448
C	-2.476653	4.217863	-5.118676
H	-2.259785	2.161961	-4.431226
C	-2.466773	5.571290	-4.739939
H	-2.637716	3.938815	-6.171783
C	-2.256223	5.927811	-3.400916
H	-2.618391	6.358799	-5.494780
H	-2.231693	6.990101	-3.113935
C	5.120655	10.082862	1.256524
C	5.719633	8.868103	1.624572
C	3.962473	10.074479	0.461987
H	5.554745	11.037221	1.593341
C	3.402407	8.858862	0.044198
H	3.483623	11.022524	0.171748
C	4.002222	7.634306	0.401578
H	2.482595	8.842150	-0.561210
C	5.168094	7.650177	1.195448
H	5.656886	6.709319	1.489139
H	6.625069	8.865889	2.251673
C	3.660964	5.935392	-2.993180
C	4.244949	5.764838	-1.723486
C	4.417801	5.737281	-4.158301
H	2.602002	6.225597	-3.057947
C	5.767536	5.365611	-4.069592
H	3.941934	5.868178	-5.142367
C	6.361768	5.204442	-2.807056
H	6.358714	5.199616	-4.983696
C	5.606554	5.407999	-1.643771
H	7.421009	4.914261	-2.726549
H	6.092279	5.286444	-0.663567
C	2.371961	1.430811	0.208689
C	2.643296	0.138254	-0.295600
C	3.426737	2.400661	0.417363
C	4.743458	2.022235	0.043787
C	4.997334	0.751663	-0.488683
H	5.568756	2.740516	0.155914
C	3.961898	-0.188426	-0.653764
H	6.025206	0.488458	-0.785464
H	4.177851	-1.185124	-1.071053
P	-1.661901	5.455403	-0.691130
P	3.212085	6.067769	-0.203955
N	1.084765	1.854322	0.453350
N	-1.287845	3.039171	0.619454
N	3.027348	3.585803	0.960828
C	1.502306	-0.856626	-0.398276
H	1.634173	-1.500097	-1.295656
H	1.558303	-1.558529	0.471091
C	3.835803	4.760713	1.035801
H	4.918452	4.565731	0.878103
H	3.740782	5.256012	2.030129
C	-2.314383	4.001424	0.385959
H	-2.676326	4.488060	1.321554
H	-3.207733	3.584070	-0.128299
Bi	0.752870	3.791892	1.477458
W	0.741783	5.991779	-0.445219
C	1.059097	4.442503	-1.756264
C	0.606982	7.360709	-1.826247
C	0.558991	7.351332	1.056642
O	0.500161	8.125486	1.941031
O	1.259781	3.551128	-2.485298
O	0.504383	8.192413	-2.666586
F	0.675566	3.112070	3.503511

Computational Analysis of 2

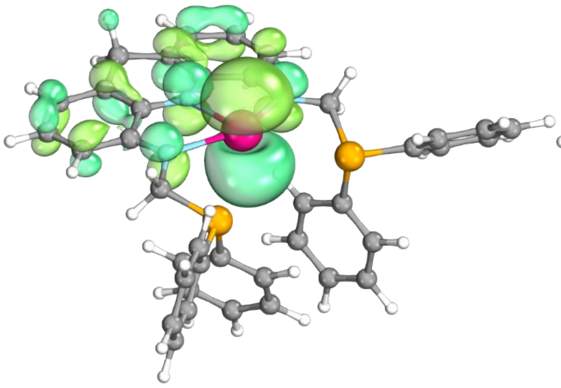

LUMO (1.78 eV)

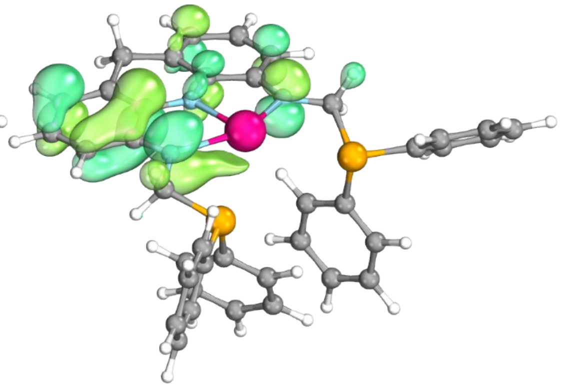

HOMO (-6.26 eV)

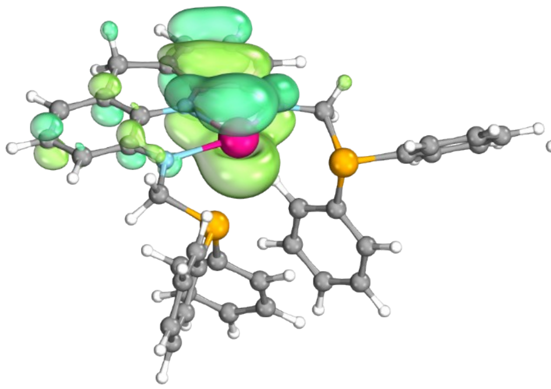

HOMO-1 (-6.30 eV)

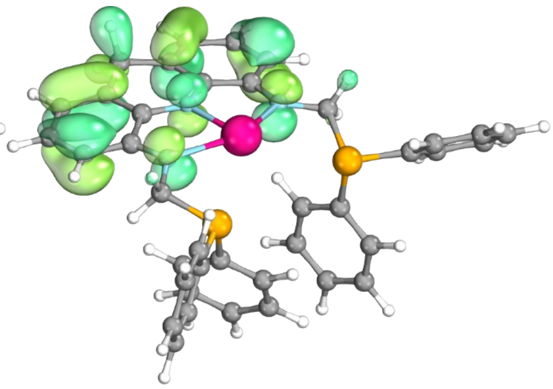

HOMO-2 (-7.12 eV)

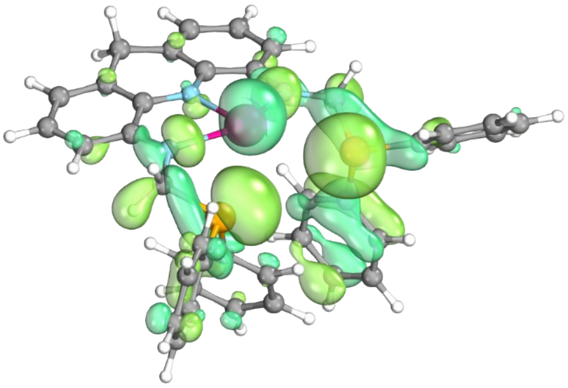

HOMO-3 (-7.43 eV)

Figure S43. Frontier molecular orbitals of **2**.

The ORCA output for the first two transition is given in the following. State 1 can be best described as a HOMO(169a)→LUMO(170a) excitation, while the second has dominant HOMO-1(168a)→LUMO(170a) character. Accumulation of electron density in the difference density plots is depicted in green and depletion in red.

```
STATE  1:  E=   0.087571 au      2.383 eV    19219.6 cm**-1
   168a -> 170a  :     0.129236 (c=  0.35949367)
   169a -> 170a  :     0.810314 (c= -0.90017442)
```

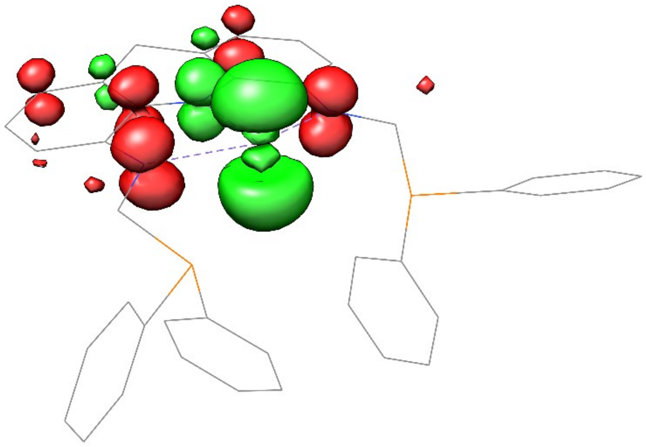

Figure S44. Difference density of state 1 of **2**.

```
STATE  2:  E=   0.099761 au      2.715 eV    21895.0 cm**-1
   166a -> 170a  :     0.044033 (c=  0.20984117)
   167a -> 170a  :     0.013098 (c= -0.11444465)
   168a -> 170a  :     0.721194 (c= -0.84923155)
   169a -> 170a  :     0.115918 (c= -0.34046694)
   169a -> 177a  :     0.014104 (c= -0.11875971)
```

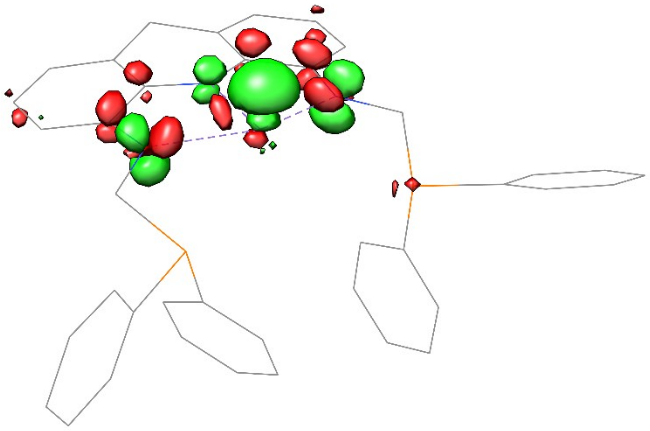

Figure S45. Difference density of state 2 of **2**.

To understand the Bi–N bond, we conducted additional CASSCF calculations. We selected an active space with two electrons in two orbitals (CAS(2/2)). These initial orbitals were derived from DFT calculations and represent the Bi-N π-bonding and π-antibonding orbitals. When using natural orbitals for the active space, the resulting CASSCF orbitals closely resemble the DFT orbitals, indicating a lack of significant multireference character. To determine the bond polarity, we transformed the active orbitals into localized orbitals. The resulting wavefunction consists of three resonance structures, with the second structure (**II**) having a higher weight than the third structure (**III**), suggesting a slight polarization of the Bi-N bond towards nitrogen. However, the dominant resonance structure (**I**) indicates a non-polarized, covalent 2c-2e π-bond between Bi and N. The high covalency makes it challenging to assign a formal oxidation state to Bi, but the presence of a Bi(III) center could be justified in extreme cases based on the higher weight of **II** compared to **III**.

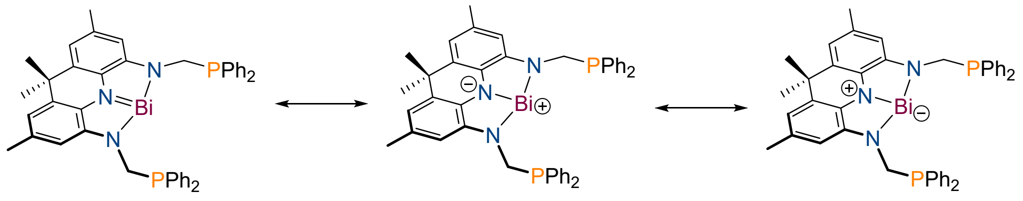

I, 75% **II**, 17% **III**, 8%

Figure S46. Resonance structures of **2** and their respective weights

Computational Analysis of 3

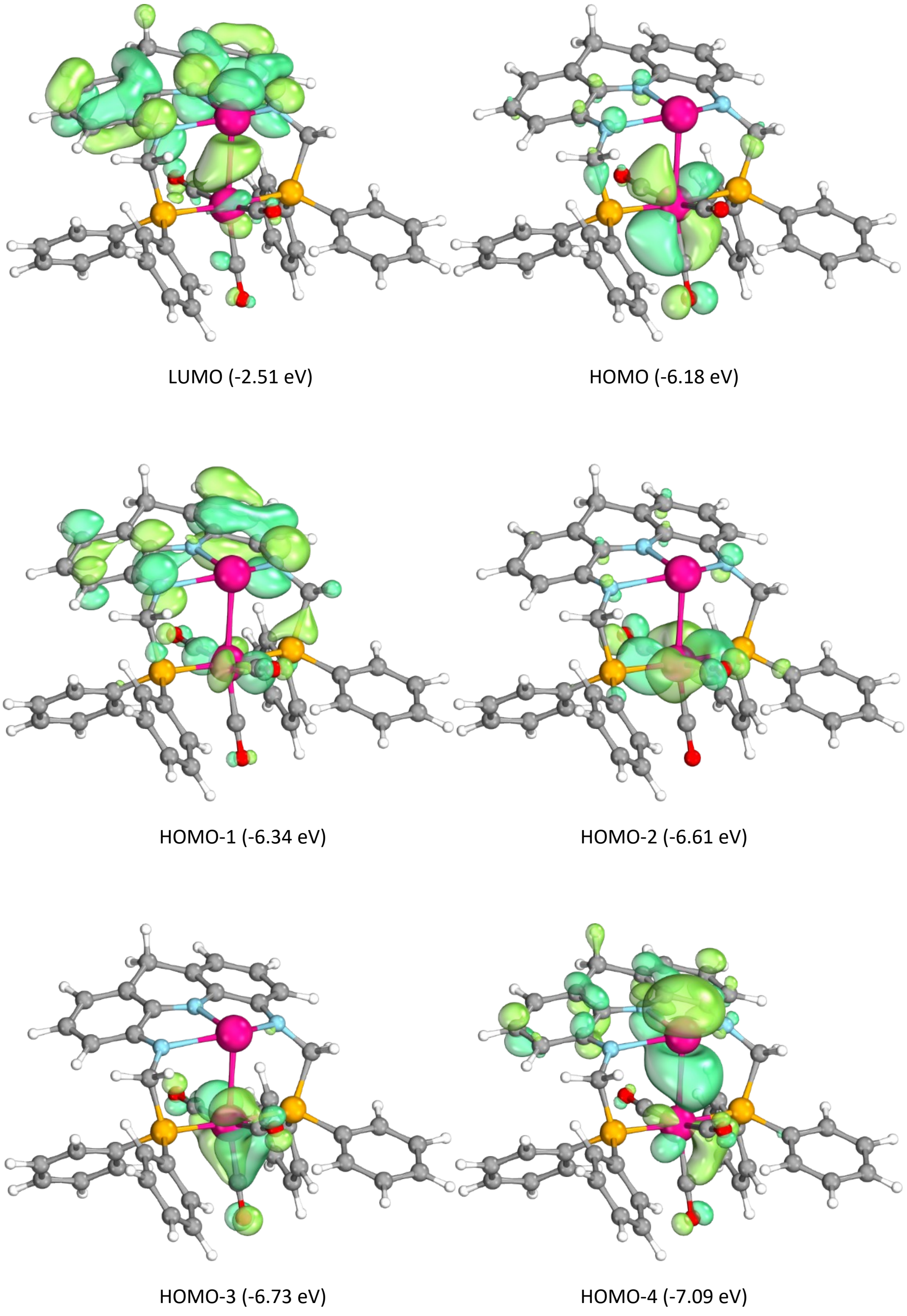

Figure S47. Frontier molecular orbitals of **3**.

The ORCA output for the two observed intense transitions are given in the following. State 1-3 are predicted to be of low intensity and were not further interpreted. State 4 can be described as an electronic transition of ligand and metal based orbitals into the LUMO(198a) that features major acridane π* character in an antibonding interaction with the Bi(6p) orbital. Accumulation of electron density in the difference density plots is depicted in green and depletion in red.

```
STATE  4:  E=   0.075107 au      2.044 eV    16484.0 cm**-1
   194a -> 198a  :     0.179441 (c=  0.42360426)
   195a -> 198a  :     0.214403 (c=  0.46303618)
   196a -> 198a  :     0.531331 (c= -0.72892465)
```

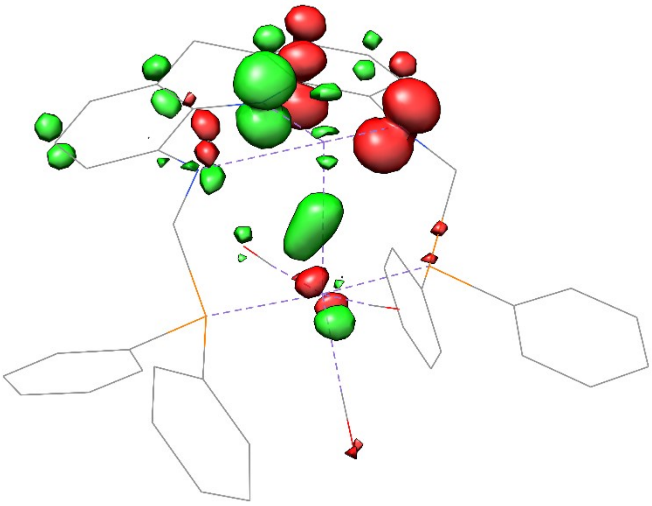

Figure S48. Difference density of state 4 of **3**.

State 5 features dominant charge transfer from the HOMO-4 (193a) into the LUMO showcasing the population of the Bi(6p) orbital arising from the redox-active nature of the PBiP pincer ligand.

```
STATE  5:  E=   0.089336 au      2.431 eV    19607.0 cm**-1
   192a -> 198a  :     0.021252 (c= -0.14578136)
   193a -> 198a  :     0.855611 (c= -0.92499223)
   197a -> 198a  :     0.025504 (c=  0.15969874)
```

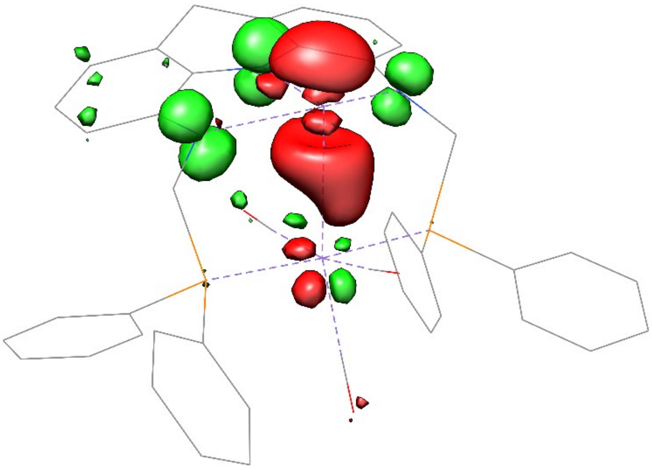

Figure S49. Difference density of state 5 of **3**.

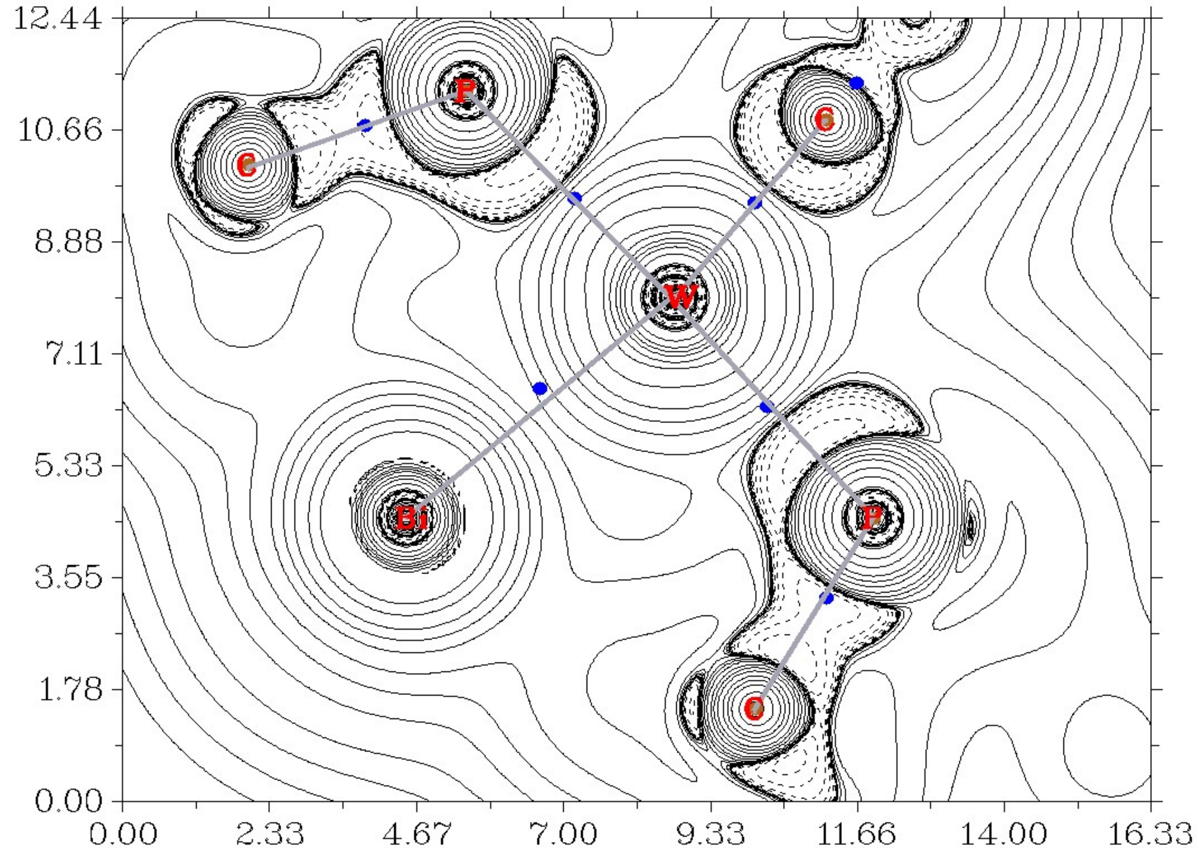

Figure S50. Laplacian plot of 3 in the Bi-W-P plane. Bond critical points are marked with blue dots.

Table S2. Characteristic BCP descriptors obtained from the QTAIM analysis of **2** and **3**. ρ(r): density at the BCP, $\nabla^2\rho(r)$ Laplacian of the density at the BCP, G(r) kinetic energy density at the BCP, V(r) potential energy density at the BCP, H(r) energy density at the BCP.

Compound	Bond	*ρ(r)*	*$\nabla^2\rho(r)$*	*G(r)*	*V(r)*	*H(r)*	*ε*
**2**	Bi-N	0.069	0.170	0.058	−0.074	−0.016	0.25
	Bi-N	0.070	0.174	0.060	−0.076	−0.016	0.25
	Bi-N (central)	0.086	0.208	0.078	−0.103	−0.026	0.26
**3**	Bi-N	0.071	0.163	0.058	−0.075	−0.017	0.26
	Bi-N	0.063	0.156	0.052	−0.065	−0.013	0.25
	Bi-N (central)	0.082	0.192	0.071	−0.094	−0.023	0.26
	W-Bi	0.044	0.052	0.024	−0.034	−0.011	0.26
	W-P	0.076	0.150	0.061	−0.085	−0.024	0.21
	W-P	0.0744	0.140	0.058	−0.080	−0.023	0.22

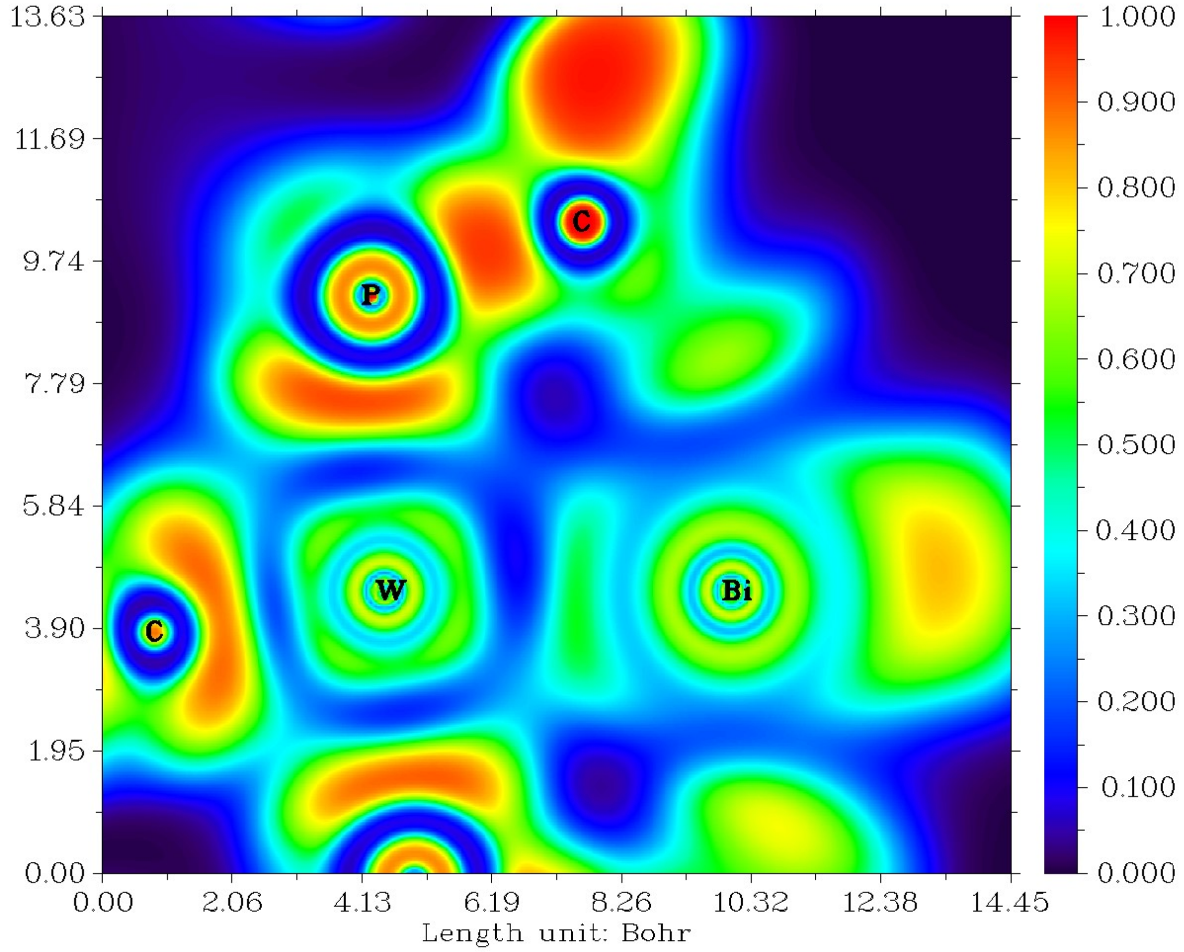

Figure S51. ELF plot of 3 in the Bi-W-P plane, showing accumulation of electron density between the W and Bi center.

Since a topology analysis of the Laplacian as well as the ELF of complex **3** indicate the presence of attractive interactions between Bi and W, we conducted EDA-NOCV calculations to get more insight into the orbital interactions. Here, the interaction between the $W(CO)_3$ fragment and the ligand fragment was studied. The six orbital pairs with the highest interaction energies are shown in Figure S46. Donor orbitals are colored in red and acceptor orbitals are shown in green. Orbital pairs 1, 4 and 5 describe the bonding between the P atoms and the W center (1: σ-donation, 4,5: π-backdonation). Orbital pairs 2 and 6 show donation of electron density from the W center to the Bi center. Orbital pair 3 shows that donation of electron density from Bi to W is however also a significant contributor to the Bi-W bonding. The dual acceptor and donor properties of the Bi center agree with a partial occupation of the p-Orbital in Bi, which allows it to simultaneously act as an electron donor and acceptor.

**1** 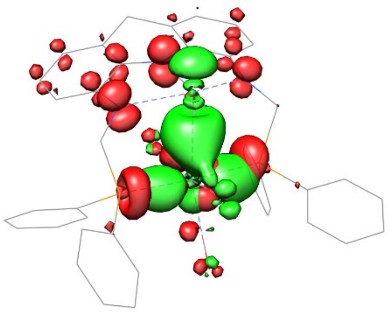	**2** 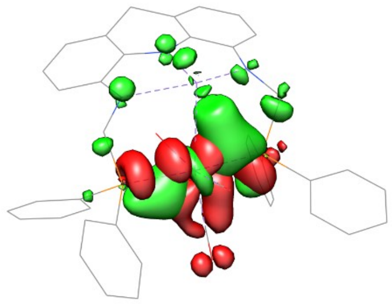
−51.6	−37.2
**3** 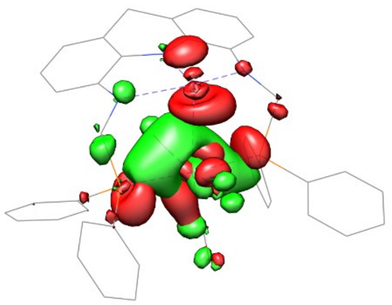	**4** 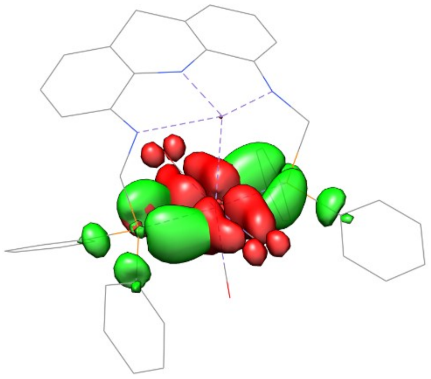
−29.9	−17.8
**5** 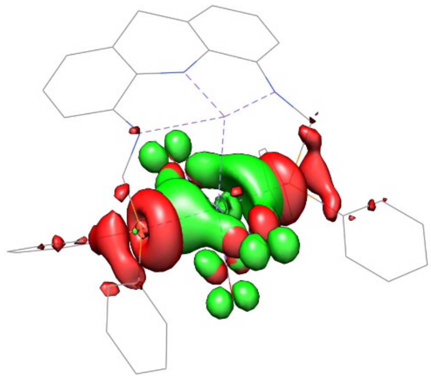	**6** 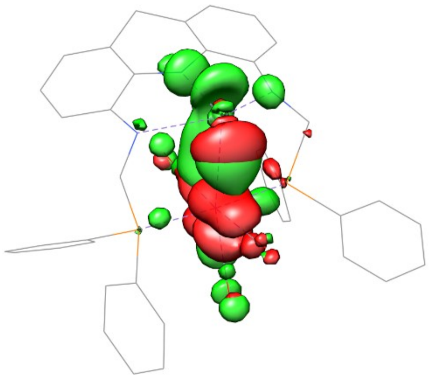
−12.8	−5.7

Figure S52. Donor and acceptor orbitals of the EDA-NOCV calculation of **3**. Orbital interaction energies are given in kcal/mol.

Computational Analysis of 4

The Mayer bond orders (see the main text) clearly indicate the presence of bonding orbital interactions between Bi and Cl and Bi and Au, respectively. To get a more detailed insight, EDA-NOCV calculations were performed. Here, the interaction between the Au_3Cl_2 fragment and the Bi_3 fragment of the cluster was studied. The seven orbital pairs with the highest interaction energies are shown in Figure S47. Donor orbitals are colored in red and acceptor orbitals are shown in green. Pairs 1 – 3 account for P–Au bonding. Orbital pairs 4 – 6 are indicative of the redox-active nature of the NNN-ligand and the donation from the Bi(6p) orbital towards the Au centers. Pairs 4, 6 and 8 show the transfer of electron density from the Bi-centers to the ligand backbone, but also Bi-Au bonding interactions. Pair 5 clearly show the transfer of electron density from the NNN-ligand into Au-Bi bonding orbitals. Pair 7 shows an orbital pair which accounts for multicenter bonding between the Bi and the Cl atoms. Finally, pair 8, undoubtledly shows a bonding interaction between Au and Bi, where the Bi centers bridge the Au centers via their p-orbitals.

**1** 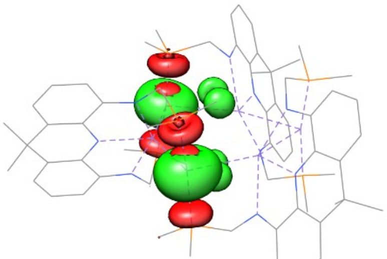	**2** 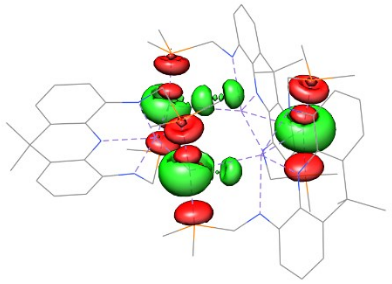
−99.0	−95.5
**3** 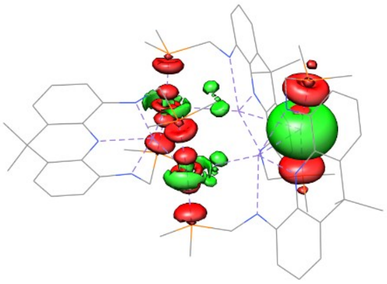	**4** 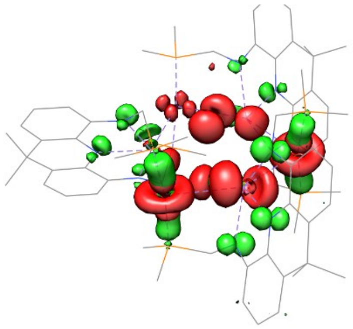
−88.7	−17.6
**5** 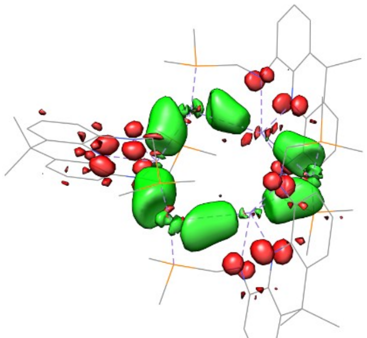	**6** 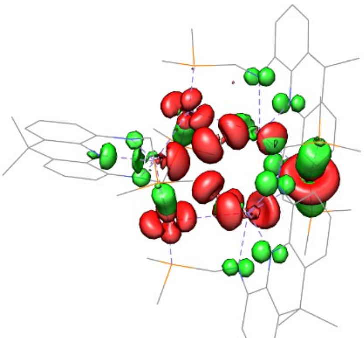
−12.5	−17.7
**7**	**8**

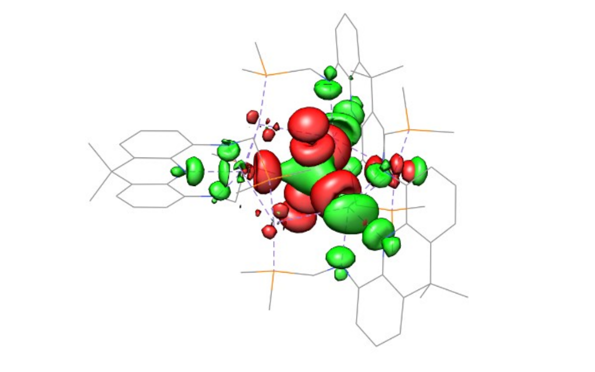	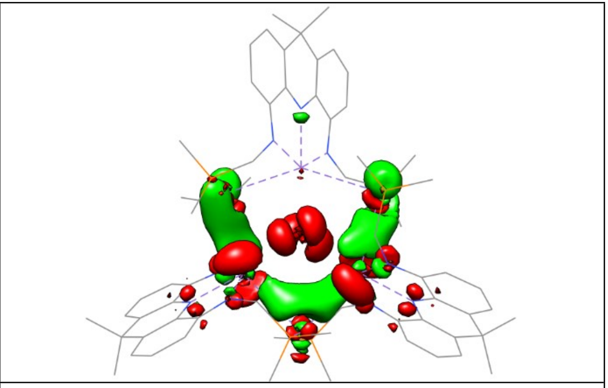
−16.6	−12.2

Figure S53. Donor and acceptor orbitals of the EDA calculation of **4**. Orbital interaction energies are given in kcal/mol.

References

[48] J. Underhill, E. S. Yang, T. Schmidt-Räntsch, W. K. Myers, J. M. Goicoechea and J. Abbenseth, *Chem. Eur. J.*, 2023, **29**, e202203266.

[57] U. Mayer, V. Gutmann and W. Gerger, *Monatsh. Chem.*, 1975, **106**, 1235–1257.

[59] J. Ramler and C. Lichtenberg, *Chem. Eur. J.*, 2020, **26**, 10250–10258.

[60] P. Erdmann, J. Leitner, J. Schwarz and L. Greb, *ChemPhysChem*, 2020, **21**, 987–994.

[S1] C. J. Carmalt, N. A. Compton, R. J. Errington and G. A. Fisher, *Inorg. Synth.*, 1996, **31**, 99–100

[S2] Bruker, *SAINT*, *V8.40B*, Bruker AXS Inc., Madison, Wisconsin, USA.

[S3] L. Krause, R. Herbst-Irmer, G. M. Sheldrick and D. Stalke, *J. Appl. Cryst.,* 2015, **48**, 3–10.

[S4] O. V. Dolomanov, L. J. Bourhis, R. J. Gildea, J. A. K. Howard and H. Puschmann, *J. Appl. Cryst.*, 2009, **42**, 339–341.

[S5] C. R. Groom, I. J. Bruno, M. P. Lightfoot and S. C. Ward, *Acta Cryst.*, 2016, **B72**, 171–179.

[S6] D. Kratzert, *FinalCif*, *V129*, https://dkratzert.de/finalcif.html.

[S7] A. G. Buzanich, M. Radtke, K. V. Yusenko, T. M. Stawski, A. Kulow, C. T. Cakir, B. Röder, C. Naese, R. Britzke, M. Sintschuck and F. Emmerling, *J. Chem. Phys.*, 2023, **158**, 244202.

[S8] B. Ravel and M. Newville, *J. Synchrotron Rad.*, 2005, **12**, 537–541.

[S9] A. A. Mistonov, A.P. Chumakov, R.P. Ermakov, L.D. Iskhakova, A.V. Zakharova, A.V. Chumakova, K.O. Kvashnina, *J. Alloys Compd.*, 2018, **753**, 646–654.

[S10] F. Neese, F. Wennmohs, U. Becker and C. Riplinger, *J. Chem. Phys.*, 2020, **152**, 224108.

[S11] R. A. Kendall, H. A. Früchtl, *Theor. Chem. Acc.*, 1997, **97**, 158–163.

[S12] F. Neese, F. Wennmohs, A. Hansen and U. Becker, *Chem. Phys.*, 2009, **356**, 98–109.

[S13] S. Grimme, S. Ehrlich and L. Goerigk, *J. Comput. Chem.*, 2011, **32**, 1456–1465.

[S14] S. Grimme, J. Antony, S. Ehrlich and H. Krieg, *J. Chem. Phys.*, 2010, **132**, 154104.

[S15] F. Weigend and R. Ahlrichs, *Phys. Chem. Chem. Phys.*, 2005, **7**, 3297–3305.

[S16] M. Cossi, N. Rega, G. Scalmani, V. Barone, *J. Comput. Chem.*, 2003, **24**, 669–681.

[S17] J. Tao, J. P. Perdew, V. N. Staroverov, G. E. Scuseria, *Phys. Rev. Lett.*, 2003, **91**, 146401.

[S18] T. Lu, F. Chen, *J. Comput. Chem.*, 2012, **33**, 580–592.

[S19] Y. Zhao and D. G. Truhlar, *J. Phys. Chem. A*, 2005, **109**, 5656–5667.
